# Maternal adverse effects of different antenatal magnesium sulphate regimens for improving maternal and infant outcomes: a systematic review

**DOI:** 10.1186/1471-2393-13-195

**Published:** 2013-10-21

**Authors:** Emily S Bain, Philippa F Middleton, Caroline A Crowther

**Affiliations:** 1 Australian Research Centre for Health of Women and Babies, Robinson Institute, Discipline of Obstetrics and Gynaecology, School of Paediatrics and Reproductive Health, The University of Adelaide, 72 King William Road, Adelaide, South Australia, Australia; 2 Liggins Institute, The University of Auckland, Auckland, New Zealand

**Keywords:** Magnesium sulphate, Magnesium sulfate, Antenatal, Adverse effect, Systematic review

## Abstract

**Background:**

Antenatal magnesium sulphate, widely used in obstetrics to improve maternal and infant outcomes, may be associated with adverse effects for the mother sufficient for treatment cessation. This systematic review aimed to quantify maternal adverse effects attributed to treatment, assess how adverse effects vary according to different regimens, and explore women’s experiences with this treatment.

**Methods:**

Bibliographic databases were searched from their inceptions to July 2012 for studies of any design that reported on maternal adverse effects associated with antenatal magnesium sulphate given to improve maternal or infant outcomes. Primary outcomes were life-threatening adverse effects of treatment (death, cardiac arrest, respiratory arrest). For randomised controlled trials, data were meta-analysed, and risk ratios (RR) pooled using fixed-effects or random-effects models. For non-randomised studies, data were tabulated by design, and presented as RR, odds ratios or percentages, and summarised narratively.

**Results:**

A total of 143 publications were included (21 randomised trials, 15 non-randomised comparative studies, 32 case series and 75 reports of individual cases), of mixed methodological quality. Compared with placebo or no treatment, magnesium sulphate was not associated with an increased risk of maternal death, cardiac arrest or respiratory arrest. Magnesium sulphate significantly increased the risk of 'any adverse effects’ overall (RR 4.62, 95% CI 2.42-8.83; 4 trials, 13,322 women), and treatment cessation due to adverse effects (RR 2.77; 95% CI 2.32-3.30; 5 trials, 13,666 women). Few subgroup differences were observed (between indications for use and treatment regimens). In one trial, a lower dose regimen (2 g/3 hours) compared with a higher dose regimen (5 g/4 hours) significantly reduced treatment cessation (RR 0.05; 95% CI 0.01-0.39, 126 women). Adverse effect estimates from studies of other designs largely supported data from randomised trials. Case reports supported an association between iatrogenic overdose of magnesium sulphate and life-threatening consequences.

**Conclusions:**

Appropriate administration of antenatal magnesium sulphate was not shown to be associated with serious maternal adverse effects, though an increase in 'minor’ adverse effects and treatment cessation was shown. Larger trials are needed to determine optimal regimens, achieving maximal effectiveness with minimal adverse effects, for each antenatal indication for use. Vigilance in the use of magnesium sulphate is essential for women’s safety.

## Background

Magnesium sulphate has a long history of use in obstetrics. It is supported as the first line treatment for women with eclampsia [1-3], and is the drug of choice for women with severe pre-eclampsia [4]; it has been widely used as a tocolytic, however, benefit for this indication remains unproven [5,6]. Most recently antenatal magnesium sulphate has been supported for neuroprotection of the fetus, and it is thus now recommended for women at risk of very preterm birth [7].

Although life-threatening maternal adverse effects of magnesium sulphate are considered extremely rare in obstetrics [8], severe consequences of magnesium toxicity including respiratory arrest, cardiac arrest and death have been detailed in case reports. The 'well recognised’ and more commonly reported maternal adverse effects of magnesium sulphate include flushing, increased warmth and sweating due to the peripheral vasodilatory effects of magnesium, and nausea, vomiting, headaches, muscle weakness, blurred vision, and intravenous (IV) or intramuscular (IM) site pain or discomfort [8]. Though such maternal adverse effects may be considered comparatively 'minor,’ they have been associated with the need for early cessation of this therapy, which has benefits when used for maternal and fetal neuroprotection [4,7].

While maternal adverse effects following antenatal magnesium sulphate administration are well known [4-7,9], the risk of individual events is unclear, and there has been a dearth of evidence regarding how such adverse effects vary by different regimens. Variation in aspects of the regimens such as the route of administration, dose, and duration, may help to explain differences in adverse effects of magnesium sulphate experienced among women receiving treatment. Although recent evidence suggests that on average, there are not large differences in the risk estimates of adverse effects from randomised controlled trials and observational studies [10], some uncertainty remains regarding the consistency of estimates provided by diverse study designs [11].

In view of the extremely widespread use of antenatal magnesium sulphate in obstetric practice, in this systematic review we aimed to quantify the extent of maternal adverse effects attributed to treatment, explore any variation in risk estimates between study designs, and to assess how such maternal adverse effects vary according to different regimens for administration and different indications for use. Implementation of this therapy may be strengthened, and the safety improved, if guidelines and recommendations for practice can be based on such knowledge. As it is known that maternal adverse effects may affect adherence and therapy cessation, we additionally aimed to explore and better understand women’s responses to their experiences with this therapy.

## Methods

### Search strategy

Additional file 1 provides the PRISMA checklist. A comprehensive search of the bibliographic databases, MEDLINE, Embase, CENTRAL (Cochrane Central Register of Controlled Trials) and TOXLINE, was undertaken from their respective inceptions to July 2012, using a combination of MeSH and free text terms [12]. The search strategies used are given in Additional file 2. No date or language restrictions were applied, however, because of logistical constraints, for non-English papers only those with an available partial/full translation were retrieved. Conference Proceeding Citation Index-Science, OpenSIGLE, ClinicalTrials.gov, Current Controlled Trials metaRegister of Controlled Trials, International Clinical Trials Registry, and Google were additionally searched using key word searches (including to identify any publically available incident reports from patient safety organisations). The reference lists of any eligible articles identified were checked for additional references. The blog search engine blogsearch.google.com and the search engine Google, limited to Discussions, were searched using key words ([“magnesium sulfate” OR “magnesium sulphate”] AND pregnancy) (sorted by relevance). Because of logistical constraints, it was pre-specified that sampling would cease once 20 relevant blogs and 10 relevant discussion forum threads were identified, with up to a total of five relevant threads from each original site sampled, if available.

### Inclusion criteria

#### Studies

We included intervention (randomised, cluster-randomised, quasi-randomised and non-randomised comparative studies) and observational studies (cohort, case-control, cross-sectional, case series and case reports). We included studies available as abstracts only, along with full-text publications. Personal blogs and discussion forum threads from pregnancy-related internet sites were included, along with incident reports from patient safety organisations.

#### Participants, interventions and comparisons

We included women given antenatal magnesium sulphate: for pre-eclampsia/eclampsia (including when it was continued/initiated in the immediate postpartum period); for tocolysis to women in preterm labour or who had had at least one episode of threatened preterm labour; for neuroprotection of the fetus, to women considered at risk of preterm birth (less than 37 weeks’ gestation), or at term, regardless of the regimen for administration (including iatrogenic overdoses). We excluded studies where women were given oral magnesium sulphate, and where magnesium sulphate was given as an adjuvant during anaesthesia, or where magnesium sulphate was given in combination with another agent for tocolysis. We included instances where magnesium sulphate was compared to no placebo, placebo or to a different magnesium sulphate regimen, and/or, where the study’s exposure was magnesium sulphate. We excluded studies where magnesium sulphate was compared to an alternative therapy.

#### Outcomes

We included studies that reported data on maternal adverse effects associated with magnesium sulphate. Primary outcomes were life-threatening adverse effects of treatment (death, cardiac arrest, respiratory arrest). Secondary outcomes included other maternal-reported or clinical maternal adverse effects attributed to treatment (e.g. warmth and flushing, arm discomfort), outcomes associated with interventions to reduce potential/actual adverse effects (e.g. use of calcium gluconate, discontinuation of treatment), and other outcomes of interest (including caesarean section, pulmonary oedema, and postpartum haemorrhage). We used the definitions as used by the study authors.

### Study selection

After screening all titles and abstracts, we obtained the full-text article for any study which appeared to meet the inclusion criteria based on the title and/or abstract, along with any reviews that may have provided relevant references. All full-text articles and abstracts were assessed for inclusion. Each stage was carried out by one reviewer (ESB) with the second reviewer (PFM) assessing a random sample (10% of the total). We resolved any discrepancies through discussion, or if required, we consulted the third reviewer (CAC).

### Data extraction and management

Once a study was included, data were extracted using a standardised form. Data extracted included information regarding study design, participants, the magnesium sulphate regimen(s), the control/comparison if applicable, maternal adverse effects reported and results relevant to the review, the risk of bias, confounding and relevance. For personal blogs and discussion forum threads, information regarding perceived purpose and/or benefits of treatment, and women’s experiences with treatment, particularly considering adverse effects were extracted. Extraction was carried out by one reviewer (ESB), with the second reviewer (PFM) independently extracting a random sample (10% of the total, and all included randomised controlled trials). We resolved any discrepancies through discussion, or if required, we consulted the third reviewer (CAC).

### Assessment of study quality/ risk of bias

Quality appraisal of intervention studies was undertaken utilising established guidelines provided in the Cochrane Handbook for Systematic Reviews of Interventions [13]. The quality assessment of observational studies was guided by recommendations from the Cochrane Handbook on assessing the quality of non-randomised studies [13] (which highlights that attention must be paid particularly to selection bias) and principles of the Newcastle-Ottawa Scale [14], where we judged the quality of each study on three main aspects: the selection of the study groups; the comparability of the groups; and the ascertainment of either the exposure of outcome of interest for case-control or cohort studies respectively; for case series we primarily considered selection of the study group. As it has been suggested that the quality of adverse effect detection and reporting is not always adequately assessed, it was also important to consider the methods used to detect adverse effects and how rigorous these methods were, along with an assessment of incomplete reporting [12,15].

### Data synthesis and analysis

The analysis and presentation of results were categorised by study design. Statistical analyses were performed using Review Manager, version 5.1 (The Cochrane Collaboration, Copenhagen, Denmark).

For intervention studies we presented quantitative data from individual studies where possible as risk ratios (RR) with 95% confidence intervals (CI) for dichotomous outcomes. For all outcomes, we carried out analyses as far as possible on an intention-to-treat basis. Pooled estimates (summary RR with 95% CI) were calculated using fixed-effect meta-analysis (Mantel-Haenszel method) where there was a sufficient quantity of data, with clinical homogeneity. Where we considered that there was clinical heterogeneity sufficient to expect the underlying effects differed between trials, or there was substantial statistical heterogeneity (where I^2^ was greater than 30% and either T^2^ was greater than zero, or there was a low P-value (less than 0.10) in the Chi^2^ test), summary estimates were calculated using random-effects meta-analysis.

Separate comparisons were performed for those studies assessing magnesium sulphate versus no treatment/placebo, and those comparing different magnesium sulphate regimens. For all review outcomes, we conducted subgroup analyses based on indication for use (i.e. given for pre-eclampsia/eclampsia; fetal neuroprotection; tocolysis), as this was considered likely to influence outcomes. Additional subgroup analyses were planned if sufficient data were available based on aspects of the magnesium sulphate regimen (i.e. route of administration; dose). We assessed subgroup differences by interaction tests available within Review Manager, and where applicable, we have quoted the Chi^2^ statistic and P-value, and the interaction test I^2^ value. We included only primary outcomes and the outcomes: discontinuation due to adverse effects, calcium gluconate use, and 'any adverse effects’ in subgroup analyses.

For observational studies (cohort, case-control, cross-sectional, case series) we presented effect estimates where possible as percentages, RR or odds ratios (OR) with 95% CIs, adjusted RR or OR if reported with 95% CIs, or P-values only, in tabular format based on study type; we used narrative synthesis to summarise the studies. Data from case reports were tabulated and subsequently grouped according to themes. For personal blogs and discussion forum threads, relevant text was tabulated (considering perceived purpose/benefits of magnesium sulphate and experience of magnesium sulphate therapy, before, during and after treatment), and thematic analysis techniques were used to identify and summarise emerging themes.

## Results

### Study selection

**Figure 1:**
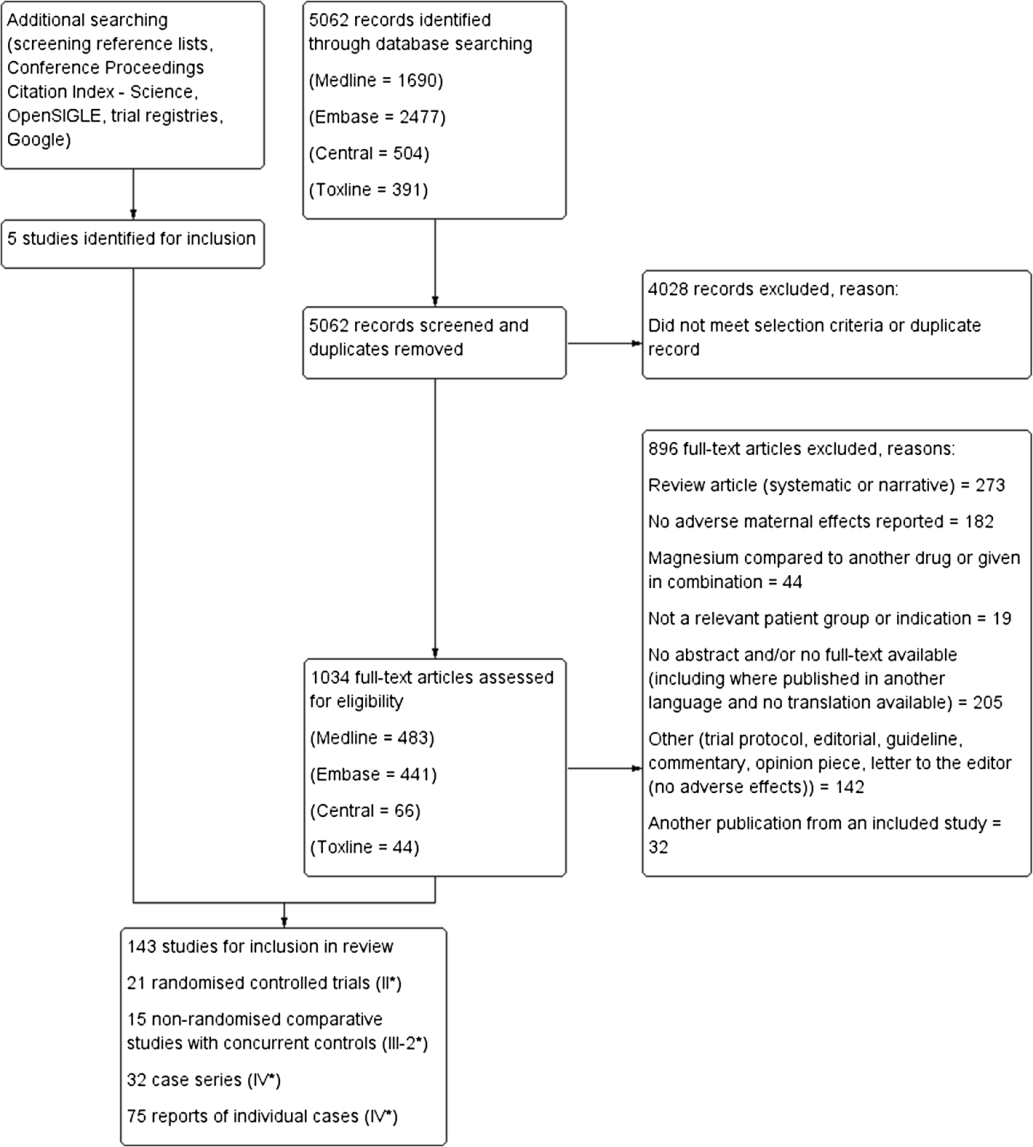
**Flow diagram of included studies.** *Numbers indicate level of evidence, according to Australian Government National Health and Medical Research Council (NHMRC) Evidence Hierarchy Available at: https://www.nhmrc.gov.au/_files_nhmrc/file/guidelines/developers/nhmrc_levels_grades_evidence_120423.pdf.

The results of the search strategy, including the sources of the studies, culling and final inclusion of studies are shown in Figure 1. The initial database searching identified 5,062 articles. Review of the abstracts/titles and exclusion of irrelevant/duplicate articles yielded 1,034 articles. Of these articles, we excluded 896 for the documented reasons. We therefore included 138 studies, along with an additional five studies identified through other searching; a total of 143 studies (see Additional file 3 for References to all included reports). In the case of multiple publications from the same study, we included the report with the most relevant data relating to adverse effects.

### Evidence from randomised controlled trials

**Figure 2:**
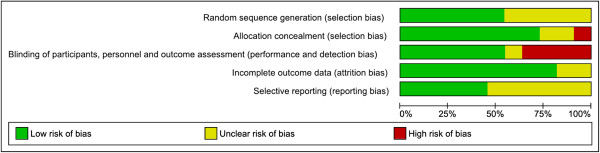
**Risk of bias for randomised controlled trials (Comparison 1).** Risk of bias graph showing review authors’ judgements about each risk of bias item presented as percentages across included studies from Comparison 1.

**Figure 3:**
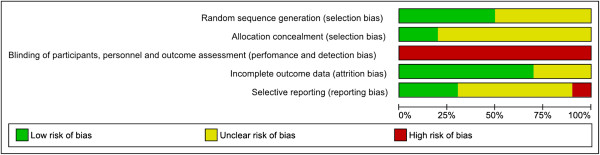
**Risk of bias for randomised controlled trials (Comparisons 2–6).** Risk of bias graph showing review authors’ judgements about each risk of bias item presented as percentages across included studies from Comparisons 2–6.

**Figure 4:**
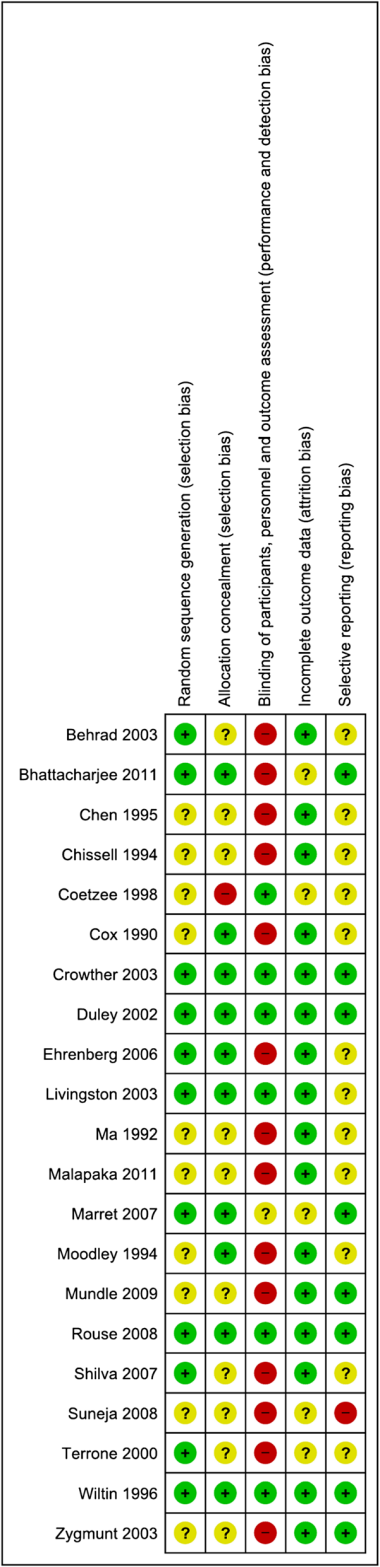
**Risk of bias for randomised controlled trials (Comparisons 1–6).** Risk of bias summary showing review authors’ judgements about each risk of bias item for included studies from Comparisons 1–6. Each risk of bias item is judged as at a low risk of bias, unclear risk of bias or high risk of bias.

Twenty one randomised trials (16,812 women) were included, the characteristics of which are detailed in Additional file 4, and the risk of bias assessment presented in Figures 2, 3 and 4. The trials assessed a variety of different treatment regimens with varying comparators, and are therefore assessed under six different comparisons:

1. Magnesium sulphate versus placebo or no treatment (11 trials).

2. Lower dose versus higher dose magnesium sulphate IM maintenance (2 trials).

3. Magnesium sulphate IV maintenance versus IM maintenance (3 trials).

4. Short versus standard (24 hour) postpartum magnesium sulphate maintenance (2 trials).

5. Lower dose versus higher dose magnesium sulphate IV maintenance (2 trials).

6. 'Ready-to-use’ magnesium sulphate solution versus a reference drug requiring dilution (1 trial).

Considering the risk of bias for trials in Comparison 1 (magnesium sulphate versus placebo or no treatment), sequence generation and allocation concealment were adequate in the majority of trials (6/11 and 8/11 trials respectively) (Figure 2). For other trials, it was unclear whether this was adequate, and for one trial, allocation concealment was not considered adequate. For six trials, blinding of personnel, women and outcome assessors was considered adequate, whilst for four trials this was not considered adequate, and for one trial this was unclear. The randomised trials in Comparisons 2-6 (different magnesium sulphate regimens) were considered at a comparatively higher risk of bias overall (Figure 3). For the majority of trials, it was unclear whether sequence generation and allocation concealment were adequate (5/10 and 8/10 trials respectively). Blinding of personnel and women was not possible in any of the trials; none of the trials reported that outcomes were assessed in a blinded manner.

### Comparison 1: magnesium sulphate versus placebo or no treatment

This comparison included 11 trials with 15,709 women [16-26]. In six trials, the indication for use of magnesium sulphate was the prevention or treatment of eclampsia; for three trials, the indication was fetal neuroprotection, and for two trials, the prevention of preterm birth (see Table 1 and Additional file 5 for effect estimates and forest plots for outcomes in Comparison 1).

**Table 1:** Adverse effect estimates from randomised controlled trials (Comparison 1)

**Outcome or subgroup**	**Studies**	**Participants**	**Method (I**^**2**^(%))*	**RR (95% CI)**
**Comparison 1: Magnesium sulphate versus placebo or no treatment**				
**1.1 Death**	5 [17,19,20,23,25]	14662	F (0)	0.53 (0.26, 1.09)
1.1.1 Treatment of pre-eclampsia/eclampsia	2 [17,20]	10795	F (0)	0.53 (0.26, 1.09)
1.1.2 Fetal neuroprotection	3 [19,23,25]	3867	F (NA)	No deaths
1.1.3 LD only	1 [23]	564	F (NA)	No deaths
1.1.4 LD and MD	4 [17,19,20,25]	14098	F (0)	0.53 (0.26, 1.09)
1.1.5 4 g IV LD and MD	3 [17,19,20]	11857	F (0)	0.53 (0.26, 1.09)
1.1.6 5–6 g IV LD and MD	1 [25]	2241	F (NA)	No deaths
1.1.7 1 g/hour IV MD	3 [17,19,20]	7264	F (0)	0.41 (0.12, 1.43)
1.1.8 2–3 g/hour IV MD	1 [25]	2241	F (NA)	No deaths
1.1.9 IM MD	1 [20]	4593	F (NA)	0.61 (0.25, 1.48)
**1.2 Cardiac arrest**	4 [19,20,23,25]	13977	F (NA)	0.80 (0.21, 2.98)
1.2.1 Treatment of pre-eclampsia/eclampsia	1 [20]	10110	F (NA)	0.80 (0.21, 2.98)
1.2.2 Fetal neuroprotection	3 [19,23,25]	3867	F (NA)	No cardiac arrests
1.2.3 LD only	1 [23]	564	F (NA)	No cardiac arrests
1.2.4 LD and MD	3 [19,20,25]	13413	F (NA)	0.80 (0.21, 2.98)
**1.3 Respiratory arrest**	4 [19,20,23,25]	13977	F (NA)	2.50 (0.49, 12.88)
1.3.1 Treatment of pre-eclampsia/eclampsia	1 [20]	10110	F (NA)	2.50 (0.49, 12.88)
1.3.2 Fetal neuroprotection	3 [19,23,25]	3867	F (NA)	No respiratory arrests
1.3.3 LD only	1 [23]	564	F (NA)	No respiratory arrests
1.3.4 LD and MD	3 [19,20,25]	13413	F (NA)	2.50 (0.49, 12.88)
**1.4 Discontinuation due to adverse effects**	5 [18-20,25,26]	13666	F (0)	**2.77 (2.32, 3.30)**
1.4.1 Treatment of pre-eclampsia/eclampsia	2 [20,26]	10245	F (0)	**2.69 (2.18, 3.31)**
1.4.2 Fetal neuroprotection	2 [19,25]	3265	F (0)	**2.81 (2.01, 3.93)**
1.4.3 Tocolysis	1 [18]	156	F (NA)	**17.88 (1.05, 304.57)**
1.4.4 LD and MD	5 [18-20,25,26]	13666	F (0)	**2.77 (2.32, 3.30)**
1.4.5 4 g IV LD and MD	3 [18-20]	11328	F (0)	**2.75 (2.28, 3.31)**
1.4.6 5–6 g IV LD and MD	2 [25,26]	2338	F (0)	**2.94 (1.69, 5.12)**
1.4.7 1 g/hour IV MD	1 [19]	1062	F (NA)	**2.74 (1.81, 4.15)**
1.4.8 2–3 g/hour IV MD	3 [18,25,26]	2494	F (0)	**3.38 (1.97, 5.78)**
**1.5 Given calcium gluconate**				
1.5.1 Treatment of pre-eclampsia/eclampsia	2 [17,20]	10795	F (0)	1.35 (0.63, 2.88)
1.5.2 4 g IV LD and MD	2 [17,20]	10795	F (0)	1.35 (0.63, 2.88)
**1.6 Intensive care unit admission**	2 [19,20]	11172	F (NA)	0.97 (0.72, 1.30)
1.6.1 Treatment of pre-eclampsia/eclampsia	1 [20]	10110	F (NA)	0.97 (0.72, 1.30)
1.6.2 Fetal neuroprotection	1 [19]	1062	F (NA)	No admissions
**1.7 Any side effects**	4 [19,20,22,25]	13322	R (98)	**4.62 (2.42, 8.83)**
1.7.1 Treatment of pre-eclampsia/eclampsia	1 [20]	9992	F (NA)	**5.26 (4.59, 6.03)**
1.7.2 Fetal neuroprotection	2 [19,25]	3265	R (98)	**3.82 (1.38, 10.59)**
1.7.3 Tocolysis	1 [22]	65	F (NA)	**26.71 (1.64, 435.03)**
1.7.4 LD and MD	4 [19,20,22,25]	13322	R (98)	**4.62 (2.42, 8.83)**
1.7.5 4 g IV LD and MD	2 [19,20]	11054	R (99)	**3.52 (1.49, 8.32)**
1.7.6 5–6 g IV LD and MD	2 [22,25]	2268	F (5)	**6.28 (5.36, 7.35)**
1.7.7 1 g /hour IV MD	2 [19,20]	6501	R (98)	**3.31 (1.59, 6.88)**
1.7.8 2–3 g/hour IV MD	2 [22,25]	2268	F (5)	**6.28 (5.36, 7.35)**
1.7.9 IM MD	1 [20]	4553	F (NA)	**5.84 (4.80, 7.09**)
**1.8 Respiratory depression/other respiratory problems**	5[17-20,25]	14098	F (29)	**1.41 (1.07, 1.86)**
1.8.1 Treatment of pre-eclampsia/eclampsia	2 [17,20]	10677	F (0)	**1.98 (1.24, 3.15)**
1.8.2 Fetal neuroprotection	2 [19,25]	3265	F (29)	1.12 (0.79, 1.59)
1.8.3 Tocolysis	1 [18]	156	F (NA)	3.16 (0.13, 76.30)
**1.9 Absent or reduced tendon reflexes**	3 [17,20,23]	11241	F (0)	1.01 (0.71, 1.44)
1.9.1 Treatment of pre-eclampsia/eclampsia	2 [17,20]	10677	F (0)	1.00 (0.70, 1.42)
1.9.2 Fetal neuroprotection	1 [23]	564	F (NA)	1.94 (0.18, 21.32)
**1.10 Respiratory depression and absent reflexes**				
1.10.1 Treatment of pre-eclampsia/eclampsia	3 [17,20,21]	10899	F (0)	5.96 (0.72, 49.40)
**1.11 Hypotension**	3 [18,19,23]	1782	F (0)	**1.52 (1.10, 2.11)**
1.11.1 Fetal neuroprotection	2 [19,23]	1626	F (0)	**1.51 (1.09, 2.09)**
1.11.2 Tocolysis	1 [18]	156	F (NA)	3.16 (0.13, 76.30)
**1.12 Tachycardia**				
1.12.1 Fetal neuroprotection	1 [19]	1062	F (NA)	**1.53 (1.03, 2.29)**
**1.13 Flushing and/or warmth**	5 [19,20,23,25,26]	13956	R (92)	**6.94 (4.19, 11.49)**
1.13.1 Treatment of pre-eclampsia/eclampsia	2 [20,26]	10127	R (91)	**6.39 (2.44, 16.74)**
1.13.2 Fetal neuroprotection	3 [19,23,25]	3829	R (94)	**7.55 (3.39, 16.85)**
**1.14 Nausea and/or vomiting**	4 [19,20,23,25]	13821	R (92)	**5.50 (2.29, 13.22)**
1.14.1 Treatment of pre-eclampsia/eclampsia	1 [20]	9992	F (NA)	**8.88 (5.46, 14.43)**
1.14.2 Fetal neuroprotection	3 [19,23,25]	3829	R (92)	**4.60 (1.54, 13.73)**
**1.15 Muscle weakness**	3 [16,18,20]	10212	F (0)	**15.81 (7.36, 33.96)**
1.15.1 Treatment of pre-eclampsia/eclampsia	2 [16,20]	10056	F (0)	**15.97 (7.23, 35.30)**
1.15.2 Tocolysis	1 [18]	156	F (NA)	13.68 (0.78, 238.67)
**1.16 Drowsiness or confusion**	3 [19,20,26]	11189	F (0)	**2.46 (1.83, 3.29)**
1.16.1 Treatment of pre-eclampsia/eclampsia	2 [20,26]	10127	F (0)	**2.26 (1.06, 4.85)**
1.16.2 Fetal neuroprotection	1 [19]	1062	F (NA)	**2.49 (1.82, 3.42)**
**1.17 Headache**	2 [20,23]	10556	F (0)	**2.21 (1.27, 3.86)**
1.17.1 Treatment of pre-eclampsia/eclampsia	1 [20]	9992	F (NA)	**2.12 (1.19, 3.76)**
1.17.2 Fetal neuroprotection	1 [23]	564	F (NA)	3.89 (0.44, 34.57)
**1.18 Thirst or mouth dryness**	2 [19,20]	11054	R (42)	**2.38 (1.59, 3.56)**
1.18.1 Treatment of pre-eclampsia/eclampsia	1 [20]	9992	F (NA)	**3.36 (1.72, 6.58)**
1.18.2 Fetal neuroprotection	1 [19]	1062	F (NA)	**2.11 (1.72, 2.59)**
**1.19 Dizziness**	2 [19,20]	11054	R (39)	**2.62 (1.63, 4.21)**
1.19.1 Treatment of pre-eclampsia/eclampsia	1 [20]	9992	F (NA)	**3.70 (1.84, 7.42)**
1.19.2 Fetal neuroprotection	1 [19]	1062	F (NA)	**2.21 (1.53, 3.19)**
**1.20 Sweating**				
1.20.1 Fetal neuroprotection	2 [19,25]	3265	R (95)	**6.37 (1.96, 20.65)**
**1.21 Itching and/or tingling**				
1.21.1 Treatment of pre-eclampsia/eclampsia	1 [20]	9992	F (NA)	**14.98 (1.98, 113.38)**
**1.22 Blurred vision**				
1.22.1 Fetal neuroprotection	1 [19]	1062	F (NA)	**2.34 (1.32, 4.14)**
**1.23 Slurred speech**				
1.23.1 Treatment of pre-eclampsia/eclampsia	1 [26]	135	F (NA)	3.04 (0.13, 73.42)
**1.24 Problems at the IV site or arm discomfort**	3 [19,20,25]	8704	R (92)	**6.34 (3.10, 12.98)**
1.24.1 Treatment of pre-eclampsia/eclampsia	1 [20]	5439	F (NA)	**3.05 (2.15, 4.32)**
1.24.2 Fetal neuroprotection	2 [19,25]	3265	F (NA)	**9.11 (7.18, 11.55)**
**1.25 Problems at the IM site**				
1.25.1 Treatment of pre-eclampsia/eclampsia	1 [20]	4553	F (NA)	**1.49 (1.25, 1.79)**
**1.26 Caesarean section**	10 [16-21,23-26]	14105	F (0)	**1.04 (1.00, 1.08)**
1.26.1 Treatment of pre-eclampsia/eclampsia	6 [16,17,20-22,26]	10096	F (0)	**1.05 (1.01, 1.10)**
1.26.2 Fetal neuroprotection	3 [19,23,25]	3853	F (19)	1.00 (0.93, 1.08)
1.26.3 Tocolysis	1 [18]	156	F (NA)	0.90 (0.45, 1.82)
**1.27 Postpartum haemorrhage**	4 [19,20,23,26]	10535	F (0)	0.94 (0.87, 1.04)
1.27.1 Treatment of pre-eclampsia/eclampsia	2 [20,26]	8909	R (43)	1.31 (0.39, 4.41)
1.27.2 Fetal neuroprotection	2 [19,23]	1626	F (0)	0.84 (0.61, 1.15)
**1.28 Pulmonary oedema**	4 [20,21,24,25]	12787	F (8)	1.12 (0.72, 1.74)
1.28.1 Treatment of pre-eclampsia/eclampsia	3 [20,21,24]	10560	F (0)	0.95 (0.60, 1.57)
1.28.2 Fetal neuroprotection	1 [25]	2227	F (NA)	2.80 (0.75, 10.53)

I^2^ statistics is a test of heterogeneity; where I^2^ was > 30% summary estimates were calculated using random-effects meta-analysis; the bold effect estimates indicate statistical significance.

Abbreviations: *CI* confidence interval, *F* fixed-effect, *g* gram, *IM* intramuscular, *IV* intravenous, *LD* loading dose, *MD* maintenance dose, *NA* not applicable, *R* random-effects, *RR* risk ratio.

#### Life-threatening adverse effects of treatment

No significant differences were seen between magnesium sulphate and placebo/no treatment for maternal death (RR 0.53; 95% CI 0.26 to 1.09; 5 trials, 14,662 women; Analysis 1.1), cardiac arrest (RR 0.80; 95% CI 0.21 to 2.98; 4 trials, 13,977 women; Analysis 1.2) or respiratory arrest (RR 2.50; 95% CI 0.49 to 12.88; 4 trials 13,977 women; Analysis 1.3).

#### Interventions to limit adverse effects

Women receiving magnesium sulphate experienced a significantly increased (almost three times) risk of discontinuing treatment due to associated adverse effects (RR 2.77; 95% CI 2.32 to 3.30; 5 trials 13,666 women; Analysis 1.4). There were no significant differences between groups in the outcomes calcium gluconate administration (RR 1.35; 95% CI 0.63 to 2.88; 2 trials, 10,795 women; Analysis 1.5) and intensive care unit admission (RR 0.97; 95% CI 0.72 to 1.30; 2 trials, 11,172 women; Analysis 1.6).

#### Adverse effects associated with treatment

Women receiving magnesium sulphate were almost five times more likely to experience 'any side effects’ in the four included trials (RR 4.62; 95% CI 2.42 to 8.83; 13,322 women; Analysis 1.7). Women receiving magnesium sulphate compared with women receiving no treatment/placebo experienced an approximately 50% increased risk of hypotension (RR 1.52; 95% CI 1.10 to 2.11; 3 trials, 1,782 women; Analysis 1.11) and tachycardia (RR 1.53; 95% CI 1.03 to 2.29; 1 trial, 1,062 women; Analysis 1.12). Compared with women receiving no treatment/placebo, women receiving magnesium sulphate experienced an approximately 50% increased risk of problems at the IM injection site (RR 1.49; 95% CI 1.25 to 1.79; 1 trial, 4,553 women; Analysis 1.25), and more than six times the risk of problems/discomfort at the IV site (RR 6.34; 95% CI 3.10 to 12.98; 3 trials, 8,704 women; Analysis 1.24).

Women receiving magnesium sulphate had an approximately 50% increased risk of respiratory depression (RR 1.41; 95% CI 1.07 to 1.86; 5 trials, 14,098 women; Analysis 1.8), more than two times the risk of drowsiness/confusion (RR 2.46; 95% CI 1.83 to 3.29; 3 trials, 11,189 women; Analysis 1.16), headache (RR 2.21; 95% CI 1.27 to 3.86; 2 trials, 10,556 women; Analysis 1.17), dizziness (RR 2.62; 95% CI 1.63 to 4.21; 2 trials, 11,054 women; Analysis 1.19), mouth dryness or thirst (RR 2.38; 95% CI 1.59 to 3.56; 2 trials, 11,054 women; Analysis 1.18) and blurred vision (RR 2.34; 95% CI 1.32 to 4.14; 1 trial, 1,062 women; Analysis 1.22), more than five times the risk of nausea and/or vomiting (RR 5.50; 95% CI 2.29 to 13.22; 4 trials; 13,821 women; Analysis 1.14), nearly seven times the risk of flushing and warmth (RR 6.94; 95% CI 4.19 to 11.49; 5 trials, 13,956 women; Analysis 1.13) and sweating (RR 6.37; 95% CI 1.96 to 20.65; 2 trials, 3,265 women; Analysis 1.20), nearly 15 times the risk of itching and tingling (RR 14.98; 95% CI 1.98 to 113.38; 1 trial, 9,992 women; Analysis 1.21), and more than 15 times the risk of muscle weakness (RR 15.81; 95% CI 7.36 to 33.96; 3 trials, 10,212 women; Analysis 1.15).

There were no significant differences between groups for the outcomes absent/reduced tendon reflexes (RR 1.01; 95% CI 0.71 to 1.44; 3 trials; 11,241 women; Analysis 1.9) and slurred speech (RR 3.04; 95% CI 0.13 to 73.42; 1 trial, 135 women; Analysis 1.23).

#### Other outcomes

For women receiving magnesium sulphate compared to no treatment/placebo, a small significant increased risk of caesarean section was shown (RR 1.04; 95% CI 1.00 to 1.08; 10 trials, 14,105 women; Analysis 1.26). No differences were seen between groups for the outcomes postpartum haemorrhage (RR 0.94; 95% CI 0.87 to 1.04; 4 trials, 10,535 women; Analysis 1.27) and pulmonary oedema (RR 1.12; 95% CI 0.72 to 1.74; 4 trials, 12,787 women; Analysis 1.28).

### Subgroup analysis by indication for use

When considering indication for use, the subgroup interaction tests for the majority of outcomes were non-significant, indicating no differential effects according to the different reasons for administration (see Table 1 for effect estimates for indication for use subgroups and Additional file 5 for forest plots). While substantial statistical heterogeneity (I^2^ > 90%) was observed for the outcomes 'any side effects’, flushing and/or warmth and nausea and/or vomiting, this could not be explained by considering the indication for use of treatment. In each case the test for subgroup differences was non-significant ('any side effects’: Chi^2^ = 1.68, P = 0.43, I^2^ = 0%; Analysis 1.7) (flushing and/or warmth: Chi^2^ = 0.07, P = 0.79, I^2^ = 0%; Analysis 1.13) (nausea and/or vomiting: Chi^2^ = 1.16, P = 0.28, I^2^ = 13.9%; Analysis 1.14).

For the outcome problems at the IV site and/or arm discomfort, the subgroup interaction test indicated a significant difference between indication for use subgroups, and a possible differential effect in favour of receiving treatment for pre-eclampsia, with women receiving treatment for fetal neuroprotection being more likely to experience arm discomfort (Chi^2^ = 25.80, P = < 0.00001, I^2^ = 96.1%; Analysis 1.24). It is possible, however, that the methods used to collect information on arm discomfort/problems at the IV site differed substantially between trials, and could help to explain this observed differential effect.

### Subgroup analysis by regimen for administration

To explore the effect of aspects of the regimen for administration of magnesium sulphate on adverse effects, the trials from Comparison 1 were grouped as pre-specified where possible according to their dosage and/or route of administration (loading dose only; loading plus maintenance dose; 4 g IV loading dose plus any maintenance; 5-6 g IV loading dose plus any maintenance; 1 g/hour IV maintenance; 2-3 g/hour IV maintenance; IM maintenance).

For the outcomes maternal death, cardiac arrest, respiratory arrest and use of calcium gluconate, no significant differences were shown between the magnesium sulphate and no magnesium sulphate groups for any of the subgroups, and the subgroup interaction tests for all outcomes indicated no significant differential effects across the treatment subgroups (see Table 1 for effect estimates for regimen subgroups and Additional file 5 for forest plots). The significantly increased risk of discontinuing treatment due to adverse effects and experiencing 'any side effects’ for the magnesium sulphate group was seen across all of the different regimen subgroups (see Analyses 1.4 and 1.7); for both outcomes, the subgroup interaction tests did not indicate differential effects according to the subgroups.

### Comparison 2: lower dose versus higher dose IM maintenance: prevention or treatment of eclampsia

This comparison included two trials with 176 women with both trials assessing magnesium sulphate for eclampsia, or 'imminent eclampsia’ (see Table 2 and Additional file 5 for effect estimates and forest plots for Comparison 2). One trial compared a lower dose 'Dhaka’ regimen from Bangladesh: 4 g IV and 6 g IM as a loading dose, and 2.5 g IM every four hours as maintenance, with a higher dose 'Bhalla’ regimen: 4 g IV and 8 g IM as a loading dose, and 4 g IM every four hours as maintenance [27]. The second trial compared a loading dose of 4 g IV, and 2 g IM every three hours as maintenance, with Pritchard’s regimen (a loading dose of 4 g IV and 10 g IM, and 5 g IM every four hours as maintenance) [28].

**Table 2:** Adverse effect estimates from randomised controlled trials (Comparisons 2–4)

**Outcome or subgroup**	**Studies**	**Participants**	**Method (I**^**2**^(%))*	**RR (95% CI)**
**Comparison 2: lower dose versus higher dose magnesium sulphate IM maintenance: treatment of pre-eclampsia/eclampsia**				
**2.1 Death due to 'toxicity’**				
2.1.1 4 g IV LD; 2 g/3 h IM MD versus Pritchard’s regimen^	1 [28]	126	F (NA)	0.25 (0.01, 6.05)
**2.2 Stopped due to 'toxicity’**				
2.2.1 4 g IV LD; 2 g/3 h IM MD versus Pritchard’s regimen^	1 [28]	126	F (NA)	**0.05 (0.01, 0.39)**
**2.3 Deferred or skipped doses**	2 [27,28]	176	F (0)	**0.36 (0.20, 0.63)**
2.3.1 4 g IV LD; 2 g/3 h IM MD versus Pritchard’s regimen^	1 [28]	126	F (NA)	**0.43 (0.23, 0.83)**
2.3.2 'Dhaka’ regimen* versus 'Bhalla’ regimen~	1 [27]	50	F (NA)	**0.23 (0.07, 0.71)**
**2.4 Given calcium gluconate**				
2.4.1 'Dhaka’ regimen* versus 'Bhalla’ regimen~	1 [27]	50	F (NA)	0.25 (0.60, 1.06)
**2.5 Respiratory depression**				
2.5.1 4 g IV LD; 2 g/3 h IM MD versus Pritchard’s regimen^	1 [28]	126	F (NA)	0.25 (0.01, 6.05)
**2.6 Absent tendon reflexes**	2 [27,28]	176	F (0)	**0.21 (0.10, 0.46)**
2.6.1 4 g IV LD; 2 g/3 h IM MD versus Pritchard’s regimen^	1 [28]	126	F (NA)	**0.20 (0.08, 0.50)**
2.6.2 'Dhaka’ regimen* versus 'Bhalla’ regimen~	1 [27]	50	F (NA)	0.25 (0.06, 1.06)
**2.7 Gluteal abscess (pain, phlebitis, inflammation)**				
2.7.1 4 g IV LD; 2 g/3 h IM MD versus Pritchard’s regimen^	1 [28]	126	F (NA)	No gluteal abscesses
**2.8 Postpartum haemorrhage**				
2.8.1 4 g IV LD; 2 g/3 h IM MD versus Pritchard’s regimen^	1 [28]	126	F (NA)	0.38 (0.03, 4.03)
**2.9 Pulmonary oedema**				
2.9.1 4 g IV LD; 2 g/3 h IM MD versus Pritchard’s regimen^	1 [28]	126	F (NA)	0.25 (0.01, 6.05)
**Comparison 3: magnesium sulphate IV maintenance versus IM maintenance: treatment of pre-eclampsia/eclampsia**				
**3.1 Death**				
3.1.1 4 g IV LD; 0.75 g/hour IV MD versus Pritchard’s regimen^	1 [29]	137	F (NA)	0.35 (0.04, 3.27)
**3.2 Discontinuation or modification of treatment**	2 [30,31]	317	F (0)	1.46 (0.83, 2.58)
3.2.1 6 g IV LD; 2 g/hour MD versus Pritchard’s regimen^	1 [30]	17	F (NA)	3.33 (0.15, 71.90)
3.2.2 'Springfusor pump’ IV versus 'Standard’ IM regimen	1 [31]	300	F (NA)	1.41 (0.79, 2.52)
**3.3 Clinical signs of toxicity**	2 [29,30]	154	R (38)	0.82 (0.05, 12.56)
3.3.1 4 g IV LD; 0.75 g/hour IV MD versus Pritchard’s regimen^	1 [29]	137	F (NA)	0.21 (0.01, 4.27)
3.3.2 6 g IV LD; 2 g/hour MD versus Pritchard’s regimen^	1 [30]	17	F (NA)	3.33 (0.15, 71.90)
**3.4 Pain level 'acceptable’**				
3.4.1 'Springfusor pump’ IV versus 'Standard’ IM regimen	1 [31]	300	F (NA)	**4.93 (3.59, 6.78)**
**3.5 Caesarean section**	2 [29,30]	154	F (0)	1.03 (0.78, 1.35)
3.5.1 4 g IV LD; 0.75 g/hour IV MD versus Pritchard’s regimen^	1 [29]	137	F (NA)	0.99 (0.75, 1.32)
3.5.2 6 g IV LD; 2 g/hour MD versus Pritchard’s regimen^	1 [30]	17	F (NA)	1.50 (0.47, 4.76)
**3.6 Postpartum haemorrhage**				
3.6.1 4 g IV LD; 0.75 g/hour IV MD versus Pritchard’s regimen^	1 [29]	137	F (NA)	0.35 (0.04, 3.27)
**Comparison 4: short versus standard (24 hours) postpartum magnesium maintenance therapy: treatment of pre-eclampsia**				
**4.1 Toxicity**	2 [32,33]	256	F (NA)	0.25 (0.06, 1.08)
4.1.1 Short (12 h) versus standard (24 h)	1 [32]	196	F (NA)	No toxicity
4.1.2 Short (based on clinical criteria) versus standard (24 h)	1 [33]	60	F (NA)	0.25 (0.06, 1.08)
**4.2 Side effects**				
4.2.1 Short (based on clinical criteria) versus standard (24 h)	1 [33]	60	F (NA)	0.17 (0.02, 1.30)
**4.3 'Intolerance’**				
4.3.1 Short (based on clinical criteria) versus standard (24 h)	1 [33]	196	F (NA)	No intolerance

^Pritchard’s regimen: 4 g IV and 10 g IM LD; 5 g IM MD/4 hours.

*Dhaka regimen: 4 g IV and 6 g IM LD; 2.5 g IM/4 hours.

~Bhalla regimen: 4 g IV and 8 g IM LD; 4 g IM/4 hours.

I^2^statistics is a test of heterogeneity; where I^2^ was > 30% summary estimates were calculated using random-effects meta-analysis; the bold effect estimates indicate statistical significance.

Abbreviations: *CI* confidence interval, *F* fixed-effect, *g* gram, *h* hour, *IM* intramuscular, *IV* intravenous, *LD* loading dose, *MD* maintenance dose, *NA* not applicable, *R* random-effects, *RR* risk ratio.

#### Life-threatening adverse effects of treatment

No significant difference between groups was shown for the risk of 'maternal death due to toxicity’ in one trial of 126 women (RR 0.25, 95% CI 0.01 to 6.05; Analysis 2.1). No other primary review outcomes were reported.

#### Interventions to limit adverse effects

Women allocated to the lower dose regimen were significantly less likely to have treatment stopped due to 'toxicity’ in one trial, an approximate 95% relative risk reduction (RR 0.05; 95% 0.01 to 0.39; 126 women; Analysis 2.2). Women allocated to the lower dose regimen were significantly less likely to have a maintenance dose deferred or skipped due to adverse effects, an approximate 64% relative risk reduction (RR 0.36; 95% CI 0.20 to 0.63; 2 trials, 176 women; Analysis 2.3). No clear difference was shown for the need for calcium gluconate in one trial (RR 0.25; 95% CI 0.06 to 1.06; 1 trial, 50 women; Analysis 2.4).

#### Adverse effects associated with treatment

Women allocated to the lower dose regimen were significantly less likely to have absent tendon reflexes during treatment, an approximate 79% relative risk reduction (RR 0.21; 95% CI 0.10 to 0.46; 2 trials, 176 women; Analysis 2.6). There were insufficient data for reliable conclusions about the differential effects on respiratory depression in one trial of 126 women (RR 0.25; 95% CI 0.01 to 6.05; Analysis 2.5). There were no cases of gluteal abscess in one trial of 126 women measuring this outcome (Analysis 2.7).

#### Other outcomes

No significant differences were shown between groups in one trial of 126 women for the outcomes postpartum haemorrhage (RR 0.38; 95% CI 0.03 to 4.03; Analysis 2.8) and pulmonary oedema (RR 0.25; 95% CI 0.01 to 6.05; Analysis 2.9).

### Comparison 3: IV maintenance versus IM maintenance: prevention or treatment of eclampsia

This comparison included three trials with 361 women (see Table 2 and Additional file 5 for effect estimates and forest plots for Comparison 3); all trials assessed magnesium sulphate for the prevention or treatment of eclampsia. Two trials compared Pritchard’s IM regimen (a loading dose of 4 g IV and 10 g IM (5 g in each buttock), and 5 g IM in alternative buttocks every four hours as maintenance) with either a loading dose of 6 g IV, and 2 g/hour IV maintenance [30], or a loading dose of 4 g IV, and 0.75 g/hour IV maintenance [29]. The third trial did not describe its regimens, and compared the use of an IV Springfusor pump with standard hospital practice (IV loading dose; IM maintenance) [31].

#### Life-threatening adverse effects of treatment

In one trial, no significant difference was seen for the outcome maternal death (RR 0.35, 95% CI 0.04 to 3.27; 1 trial, 137 women; Analysis 3.1). There were no data on the other primary outcomes.

#### Interventions to limit adverse effects

No clear difference was seen for the outcome discontinuation or modification of treatment due to adverse effects (RR 1.46, 95% CI 0.83 to 2.58; 2 trials, 317 women; Analysis 3.2).

#### Adverse effects associated with treatment

No significant difference was seen in two trials for the outcome 'clinical signs of toxicity’ (RR 0.82; 95% CI 0.05 to 12.56; 154 women; Analysis 3.3). In one trial of 300 women, women allocated to the IV regimen were almost five times more likely to report their pain level as 'acceptable,’ compared with women allocated to the IM regimen (RR 4.93; 95% CI 3.56 to 6.78; Analysis 3.4).

#### Other outcomes

There were insufficient data for reliable conclusions about the differential effects on caesarean section (RR 1.03; 95% CI 0.78 to 1.35; 2 trials, 154 women; Analysis 3.5) and postpartum haemorrhage (RR 0.35; 95% CI 0.04 to 3.27; 1 trial, 137 women; Analysis 3.6).

### Comparison 4: short versus standard (24 hour) postpartum maintenance therapy: prevention of eclampsia

This comparison included two trials with 260 women which assessed magnesium sulphate for the prevention of eclampsia (see Table 2 and Additional file 5 for effect estimates and forest plots for Comparison 4). The trials compared individualised (short) versus standard (24 hour) postpartum maintenance therapy [32,33]. One trial compared 2 g/hour IV maintenance for 12 hours versus for 24 hours [32], whilst the other trial compared individualised maintenance (based on clinical criteria) with 24 hours maintenance; the regimens were unclear [33].

#### Life-threatening adverse effects of treatment

There were no data on the primary outcomes.

#### Adverse effects associated with treatment

There were insufficient data for reliable conclusions about the differential effects on 'toxicity’ in two trials (RR 0.23; 95% CI 0.06 to 1.08; 256 women; Analysis 4.1), or 'side effects’ in one trial (RR 0.17; 95% CI 0.02 to 1.30; 60 women; Analysis 4.2). There were no cases of 'intolerance’ among women in either group in the one trial of 196 women reporting this outcome (Analysis 4.3).

### Comparison 5: lower dose versus higher dose IV maintenance: prevention of preterm birth

This comparison included two trials with 260 women (see Table 3 and Additional file 5 for effect estimates and forest plots for Comparison 5). Both trials assessed magnesium sulphate for the prevention of preterm labour, comparing a 4 g loading dose and 2 g/hour maintenance, with either a 6 g loading dose and ≥ 2 g/hour maintenance [34] or a 4 g loading dose and 5 g/hour maintenance [35].

**Table 3:** Adverse effect estimates from randomised controlled trials (Comparisons 5–6)

**Outcome or subgroup**	**Studies**	**Participants**	**Method (I**^**2**^(%))*	**RR (95% CI)**
**Comparison 5: lower dose versus higher dose magnesium sulphate IV maintenance: tocolysis**				
**5.1 Cessation due to adverse effects**	2 [34,35]	248	F (NA)	No cessation
**5.2 No side effects**	2 [34,35]	248	R (63)	1.55 (0.94, 2.58)
5.2.1 4 g LD; 2 g/h MD versus 6 g LD; ≥ 2 g/h MD	1 [34]	100	F (NA)	1.17 (0.71, 1.91)
5.2.2 4 g LD; 2 g/h MD versus 4 g LD; 5 g/h MD	1 [35]	148	R (NA)	**1.96 (1.35, 2.84)**
**5.3 Flushing**	2 [34,35]	248	R (60)	0.61 (0.33, 1.12)
5.3.1 4 g LD; 2 g/h MD versus 6 g LD; ≥ 2 g/h MD	1 [34]	100	F (NA)	0.87 (0.46, 1.63)
5.3.2 4 g LD; 2 g/h MD versus 4 g LD; 5 g/h MD	1 [35]	148	F (NA)	**0.46 (0.29, 0.73)**
**5.4 Nausea and vomiting**				
5.4.1 4 g LD; 2 g/h MD versus 6 g LD; ≥ 2 g/h MD	1 [34]	100	F (NA)	0.79 (0.45, 1.37)
**5.5 Headache**	2 [34,35]	248	F (0)	0.56 (0.30, 1.05)
5.5.1 4 g LD; 2 g/h MD versus 6 g LD; ≥ 2 g/h MD	1 [34]	100	F (NA)	0.80 (0.23, 2.81)
5.5.2 4 g LD; 2 g/h MD versus 4 g LD; 5 g/h MD	1 [35]	148	F (NA)	0.50 (0.24, 1.03)
**5.6 Caesarean**	2 [34,35]	248	F (0)	1.11 (0.73, 1.70)
5.6.1 4 g LD; 2 g/h MD versus 6 g LD; ≥ 2 g/h MD	1 [34]	100	F (NA)	1.31 (0.78, 2.21)
5.6.2 4 g LD; 2 g/h MD versus 4 g LD; 5 g/h MD	1 [35]	148	F (NA)	0.88 (0.43, 1.80)
**5.7 Pulmonary oedema**	2 [34,35]	260	F (NA)	0.21 (0.03, 1.76)
5.7.1 4 g LD; 2 g/h MD versus 6 g LD; ≥ 2 g/h MD	1 [34]	100	F (NA)	No oedema
5.7.2 4 g LD; 2 g/h MD versus 4 g LD; 5 g/h MD	1 [35]	160	F (NA)	0.21 (0.03, 1.76)
**Comparison 6: 'ready-to-use’ magnesium sulphate solution versus a reference drug requiring dilution: tocolysis**				
**6.1 Death**	1 [36]	46	F (NA)	No deaths
**6.2 'Serious’ adverse events**	1 [36]	46	F (NA)	No serious events
**6.3 Withdrawn from the study due to adverse effects**	1 [36]	46	F (NA)	0.67 (0.12, 3.62)
**6.4 Adverse events of 'severe intensity’**	1 [36]	46	F (NA)	0.67 (0.22, 2.05)
**6.5 1 or 2 injection site changes**	1 [36]	46	F (NA)	1.00 (0.28, 3.52)
**6.6 Poor general tolerability (physician assessed)**	1 [36]	43	F (NA)	3.14 (0.13, 72.96)
**6.7 Respiratory depression**	1 [36]	46	F (NA)	0.20 (0.10, 3.95)
**6.8 Warmth (mild, severe, very severe)**	1 [36]	41	F (NA)	0.84 (0.42, 1.69)
**6.9 Nausea and/or vomiting (mild, severe, very severe)**	1 [36]	41	F (NA)	0.536 (0.11, 2.56)
**6.10 Tiredness (mild, severe, very severe)**	1 [36]	41	F (NA)	1.18 (0.57, 2.45)
**6.11 Headache (mild, severe, very severe)**	1 [36]	41	F (NA)	0.92 (0.41, 2.06)
**6.12 Dry mouth (mild, severe, very severe)**	1 [36]	41	F (NA)	0.82 (0.38, 1.77)
**6.13 Dizziness (mild, severe, very severe)**	1 [36]	41	F (NA)	1.05 (0.30, 3.64)
**6.14 Sweating (mild, severe, very severe)**	1 [36]	41	F (NA)	1.31 (0.41, 4.20)
**6.15 Skin redness (mild, severe, very severe)**	1 [36]	41	F (NA)	1.75 (0.48, 6.38)
**6.16 Burning at injection site (mild, severe, very severe)**	1 [36]	41	F (NA)	0.42 (0.16, 1.12)
**6.17 Palpitations (mild, severe, very severe)**	1 [36]	41	F (NA)	1.58 (0.29, 8.46)
**6.18 Constipation (mild, severe, very severe)**	1 [36]	41	F (NA)	4.20 (0.51, 34.44)
**6.19 Dyspnoea (mild, severe, very severe)**	1 [36]	41	F (NA)	No dyspnoea
**6.20 Heart pain (mild, severe, very severe)**	1 [36]	41	F (NA)	0.35 (0.20, 8.10)
**6.21 Agitation (mild, severe, very severe)**	1 [36]	41	F (NA)	4.20 (0.51, 34.44)

I^2^statistics is a test of heterogeneity; where I^2^ was > 30% summary estimates were calculated using random-effects meta-analysis; the bold effect estimates indicate statistical significance.

Abbreviations: *CI* confidence interval, *F* fixed-effect, *g* gram, *h* hour, *IV* intravenous, *LD* loading dose, *MD* maintenance dose, *NA* not applicable, *R* random-effects, *RR* risk ratio.

#### Life-threatening adverse effects of treatment

The two trials did not report on the review’s primary outcomes.

#### Interventions to limit adverse effects

There was no cessation due to adverse effects in either trial (Analysis 5.1).

#### Adverse effects associated with treatment

No significant difference was shown for the outcome 'no side effects’ when data for the two trials were pooled (RR 1.55; 95% CI 0.94 to 2.84; 248 women; Analysis 5.2), however there was a substantial degree of statistical heterogeneity for this outcome (I^2^ = 63%), and the subgroup interaction test indicated a potential differential effect based on the comparison regimen (Chi^2^ = 2.73, P = 0.10, I^2^ = 63.4%). In one trial, women receiving the lower dose IV maintenance regimen (2 g/hour) were significantly more likely to experience 'no side effects’ than women receiving the higher dose maintenance regimen (5 g/hour) (RR 1.96; 95% CI 1.35 to 2.84; 148 women; Analysis 5.2.2). No significant difference was shown between the low and high dose IV maintenance groups for flushing (RR 0.61; 0.33 to 1.12; 2 trials, 248 women; Analysis 5.3), however moderate statistical heterogeneity was also observed for this outcome, which may be in part explained by the differing high dose comparison regimens.

There were insufficient data for reliable conclusions about the differential effects on the risk of nausea and vomiting (RR 0.79; 95% CI 0.45 to 1.37; 1 trial, 100 women; Analysis 5.4) and headache (RR 0.56; 95% CI 0.30 to 1.05; 2 trials. 248 women; Analysis 5.5).

#### Other outcomes

No significant differences were shown between groups for the outcomes caesarean section (RR 1.11; 95% CI 0.73 to 1.70; 2 trials, 248 women; Analysis 5.6) and pulmonary oedema (RR 0.21; 95% CI 0.03 to 1.76; 2 trials, 260 women; Analysis 5.7).

### Comparison 6: ready to use solution versus reference drug requiring dilution: prevention of preterm birth

This comparison included one trial of 46 women (see Table 3). The trial compared a pre-mixed 'ready-to-use’ solution of magnesium sulphate, with a reference drug, a commercially available infusion solution concentrate requiring dilution [36]. All women were given a 4 g IV loading dose followed by 1-2 g/hour IV maintenance.

#### Life-threatening adverse effects of treatment

There were no maternal deaths, or 'serious’ adverse events in either group (Analysis 6.1 and 6.2). There were no data on other primary outcomes.

#### Interventions to limit adverse effects

There were insufficient data for reliable conclusions about the differential effects on 'withdrawing from the study due to adverse effects’ (Analysis 6.3) and one or two injection site changes (Analysis 6.5).

#### Adverse effects associated with treatment

There were insufficient data for reliable conclusions about the differential effects on any of the adverse effects reported in the trial (adverse events of severe intensity, poor general tolerability, respiratory depression, warmth, nausea and/or vomiting, tiredness, headache, dry mouth, dizziness, sweating, skin redness, burning at injection site, palpitations, constipation, dyspnoea, heart pain, agitation) (Analyses 6.4 and 6.6 to 6.21).

#### Other outcomes

The trial did not assess other outcomes of interest.

### Evidence from non-randomised comparative studies with concurrent controls

**Figure 5:**
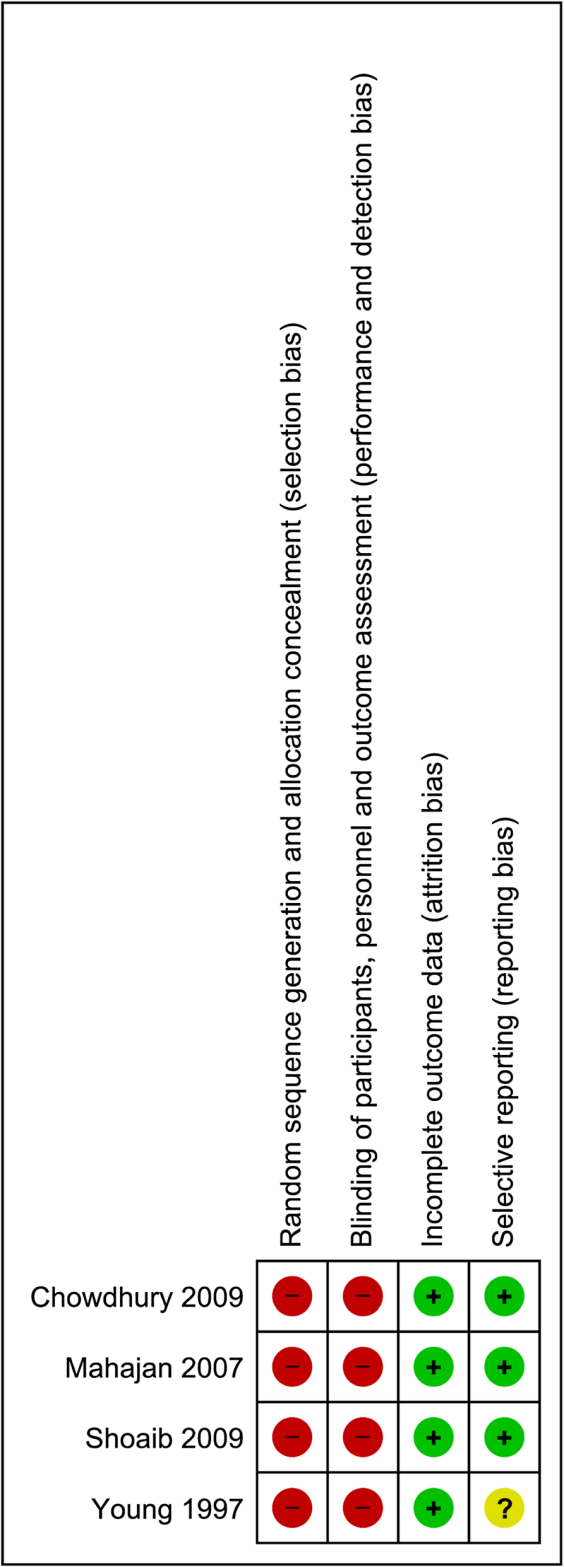
**Risk of bias for non-randomised controlled trials.** Risk of bias summary showing review authors’ judgements about each risk of bias item for included non-randomised controlled trials. Each risk of bias item is judged as at a low risk of bias, unclear risk of bias or high risk of bias.

**Figure 6:**
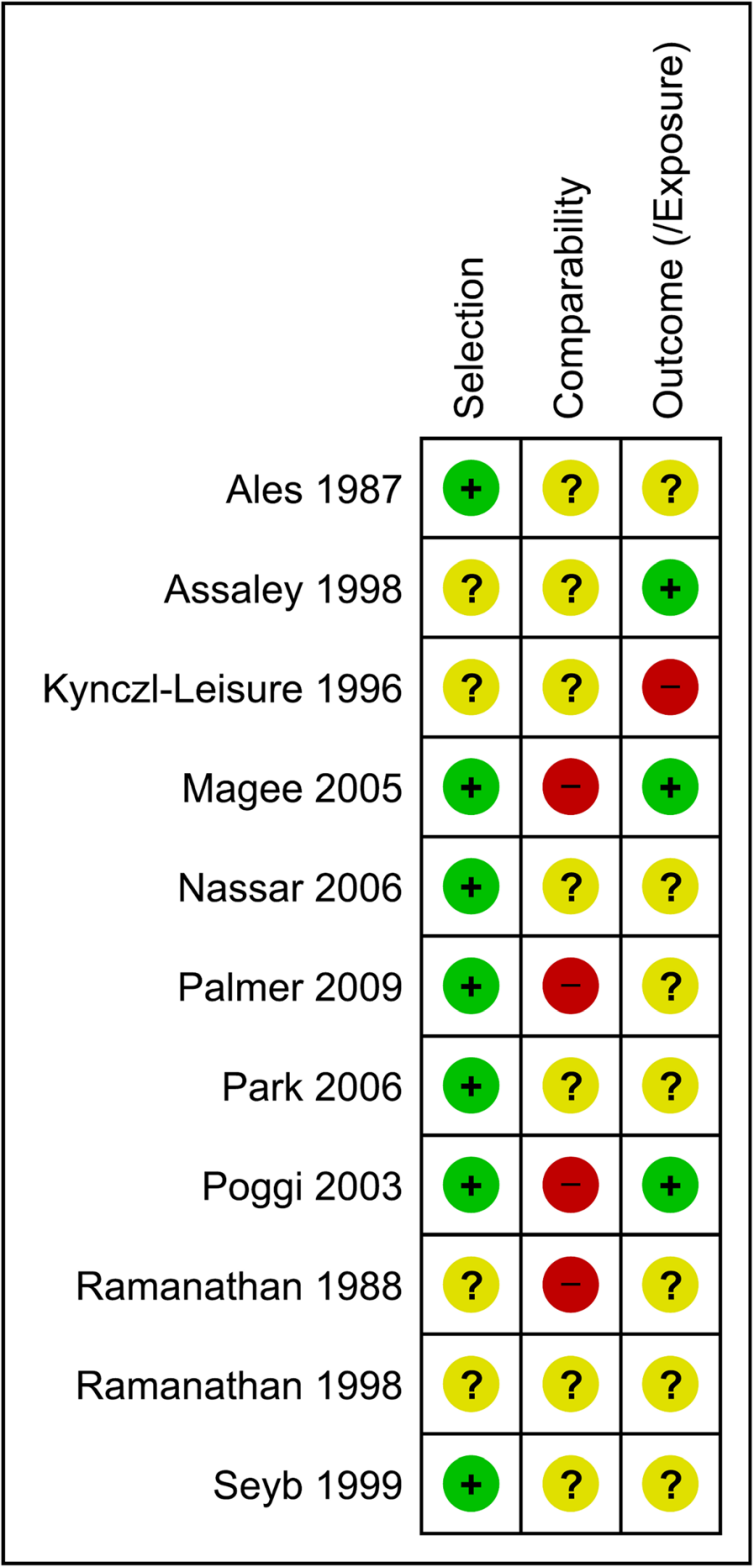
**Risk of bias for non-randomised comparative studies with concurrent controls~.** Risk of bias summary showing review authors’ judgements about each risk of bias item for included non-randomised comparative studies with concurrent controls. Each risk of bias item is judged as at a low risk of bias, unclear risk of bias or high risk of bias. ~This includes one historical control study.

Fourteen non-randomised comparative studies with concurrent controls were included (3,615 women) [37-51]: four non-randomised clinical trials (969 women) [46-49], four prospective before and after studies (78 women) [40-43], five retrospective cohort studies (2,502 women) [37-39,45,51] and one retrospective case-control study (66 women) [44]. Additionally, one historical control study was included (76 women) [50]. The detailed characteristics of the studies are presented in Additional file 4, with the risk of bias assessment presented in Figures 5 and 6. For the four non-randomised trials, sequence generation, allocation concealment and blinding were not considered adequate (Figure 5). In regards to other comparative studies, for the majority of studies, selection, according to principles of the Newcastle-Ottawa Scale [14], was considered adequate (7/11), whilst comparability was largely unclear or not considered adequate, and outcome or exposure assessment was largely unclear (Figure 6).

Results from these studies largely supported those from the randomised trials (see Tables 4 and 5), with no major maternal complications (including death and cardiac arrest) in the studies that reported these outcomes. For one retrospective study reporting on the cessation of treatment due to adverse effects [45], the percentage of women stopping treatment was similar to the percentages reported in the randomised trials (Table 5). Effect estimates from non-randomised studies for the risk of caesarean section for women receiving magnesium sulphate versus no magnesium sulphate, were notably higher than the pooled effect estimate from the randomised trials (Table 4). In two retrospective cohort studies, women receiving magnesium sulphate were significantly more likely to experience failed labour induction [39], and undergo caesarean section due to failure to progress [37] (Table 4).

**Table 4:** Adverse effect estimates from comparative studies with concurrent controls

**Study**	**Participants and comparison**	**Adverse effect**	**Estimates**	**P-value or effect estimate (95% CI)**
**Magnesium sulphate versus no magnesium sulphate**				
Ales 1987 [37]	178 women. MgSO4 for H (n = 64) v no MgSO4 (n = 114)	Caesarean	39.1 v 29.0%	**AOR 2.81 (1.99, 3.62)^**
		Caesarean (failure to progress)	72.0% v 42.4%	**OR 3.49 (1.15, 10.62)**
Seyb 1999 [38]	1561 women. MgSO4 for PE (n = 54) v no MgSO4 (n = 1507)	Caesarean	22.2% v 10.2%	**OR 2.53 (1.30, 4.91)**
		Caesarean		AOR 2.18 (1.04, 4.55)*
Park 2006 [39]	231 women. MgSO4 for PE (n = 29) v no MgSO4 (n = 202)	Failed induction of labour		**AOR 17.78 (1.62, 195.14)~**
Assaley 1998 [40]	18 women. MgSO4 for PE (n = 15) v no MgSO4 (n = 3)	Significant ↑ in bleeding time with MgSO4 (v no significant change with no MgSO4)		**P < 0.0043**
Kynczl-Leisure 1996 [41]	12 women. MgSO4 for PE (n = 9) v no MgSO4 (n = 3)	Significant ↑ in bleeding time with MgSO4 (v no significant change with no MgSO4)		**P < 0.01**
Ramanathan 1988 [42]	16 women. MgSO4 for PE (n = 10) v no MgSO4 (n = 6)	Significant ↓ in pulmonary function (FVC (L), FEV_1_(L), MVV (L)) with MgSO4 (v no significant change with no MgSO4)		**P < 0.005; P < 0.01; P < 0.02**
Ramanathan 1988 [43]	32 women. 1. Labour augmentation and MgSO4 for PE (n = 16) v 2. MgSO4 postpartum for PE (n = 6) v 3. Labour induction and no MgSO4 (n = 10)	Depression of neuromuscular transmission for Groups 1 and 2 (before MgSO4 to during MgSO4) v no changed for Group 3 (before and during induction, and postpartum)		
Poggi 2003 [44]	66 women. Pulmonary oedema (n = 15) v no pulmonary oedema (n = 51)	Case (pulmonary oedema) v control MgSO4 exposure (for PE or PTL)	93.3% v 62.7%	**P = 0.049**
**All women received magnesium sulphate: comparison based on antihypertensive agent received**				
Magee 2005 [45]	377 women who all received MgSO4 for PE. 1. Nifedipine (n = 162) v 2. Other antihypertensive (n = 32) v 3. No antihypertensive (n = 183)	Calcium gluconate given	0.5% v 3.1% v 0.0%	P = 0.30 (1v2); P = 0.47 (1v3)
		Infusion stopped due to adverse effects	1.2% v 3.1% v 4.9%	P = 0.42 (1v2); P = 0.05 (1v3)
		Infusion reduced due to adverse effects	8.0% v 3.1% v 7.7%	P = 0.47 (1v2); P = 0.90 (1v3)
		Neuromuscular weakness	53.1% v 53.1% v 44.8%	P = 0.99 (1v2); P = 0.13 (1v3)
		Absent deep tendon reflexes	5.6% v 6.3% v 3.8%	P = 0.12 (1v2); P = 0.22 (1v3)
		Weakness	15.4% v 28.1% v 10.9%	P = 0.99 (1v2); P = 0.26 (1v3)
		Respiratory depression	9.9% v 9.4% v 6.6%	P = 0.99 (1v2); P = 0.45 (1v3)
		Neuromuscular blockade	0.0% v 6.25% v 0.0%	**P = 0.03 (1v2);** P = NA (1v3)
		Maternal hypotension	41.4% v 31.3% v 53.0%	P = 0.33 (1v2); **P = 0.04 (1v3)**
		Nausea/vomiting	49.4% v 43.8% v 47.0%	P = 0.70 (1v2); P = 0.66 (1v3)
		Drowsiness/confusion	45.7% v 37.5% v 38.3%	P = 0.44 (1v2); P = 0.16 (1v3)
		Dizziness	28.4% v 25.0% v 20.8%	P = 0.83 (1v2); P = 0.10 (1v3)
		Flushing	22.2% v 15.6% v 20.8%	P = 0.48 (1v2); P = 0.74 (1v3)
		Thirst	20.4% v 21.9% v 7.1%	P = 0.81 (1v2); **P < 0.001 (1v3)**
		Respiratory problems	14.8% v 6.3% v 7.7%	P = 0.26 (1v2); **P = 0.03 (1v3)**
		Dyspnoea	8.6% v 0.0% v 4.9%	P = 0.13 (1v2); P = 0.17 (1v3)
		Pulmonary oedema	2.5% v 0.0% v 1.1%	P = 0.99 (1v2); P = 0.57 (1v3)
		Oxygen required	4.9% v 3.1% v 2.2%	P = 0.99 (1v2); P = 0.16 (1v3)
		Maternal tachycardia	22.2% v 18.8% v 14.2%	P = 0.82 (1v2); P = 0.05 (1v3)
		Itchy/tingling	14.8% v 18.8% v 15.3%	P = 0.60 (1v2); P = 0.90 (1v3)
		Tremulous	6.8% v 9.4% v 2.7%	P = 0.27 (1v3)
		Minor bleeding	4.9% v 6.3% v 0.0%	P = 0.67 (1v2); **P = 0.002 (1v3)**
		Chest pain	5.6% v 6.3% v 2.7%	P = 0.99 (1v2); P = 0.19 (1v3)

The bold effect estimates indicate statistical significance. ^Logistic regression was used to adjust for age, race, parity, physician status, obesity, gestational age, and mean arterial pressure during labour; * “Controlling for the significant confounding variables”; ~ “logistic regression analyses…adjusting for the potential confounding variables”.

Abbreviations: *AOR* adjusted odds ratio, *CI* confidence interval, *FEV*_1_ forced expiratory volume at 1 second, *FVC* forced vital capacity, *H* hypertension, *MgSO4* magnesium sulphate, *MVV* maximum voluntary ventilation, *NA* not applicable, *OR* odds ratio, *PE* pre-eclampsia, *PTL*: preterm labour, v: versus, ↑: increase, ↓: decrease.

**Table 5:** Adverse effect estimates from comparative studies with concurrent controls

**Study**	**Participants and comparison**	**Adverse effect**	**Estimates**	**Effect estimate (95% CI)**
**Magnesium sulphate versus no magnesium sulphate**				
Chowdhury 2000 [46]	630 women (E). Low dose IV (4 g IV LD over 2–3 mins; 5 g/8 h IV MD) (n = 150) v Pritchard’s IM regimen (4 g IV and 10 g IM LD; 5 g/4 h IM MD) (n = 480)	Major adverse effects; respiratory depression	0.0% v 0.0%	NA
		Absent knee jerks and oliguria; stopped dosing due to adverse effects	0.0% v 3.2%	RR 0.10 (0.01, 1.71)
		Pain at injection site	0.0% v 55.0%	**RR 0.01 (0.00, 0.10)**
Mahajan 2007 [47]	95 women (E). 1. (2 g IV and 4 g IM LD; 4 g IM/4 h) (n = 37) v 2. (2 g IV and 8 g IM LD; 4 g IM/4 h) (n = 58)	Respiratory depression	0.0% v 0.0%	NA
		Absent knee jerks and MD omitted	56.8% v 31.0%	**RR 1.83 (1.14, 2.94)**
Young 1977 [48]	144 women (PE or E). 1. (10 g IM LD; 2 g slow IV 'push’ with repeated doses every 1–2 h) (n = 97) v 2. (10 g IM LD; continuous IV 1 g/h) (n = 47)	Death	0.0% v 0.0%	NA
		Heat and flushing	92.8% v 0.0%	**RR 88.65 (5.62, 1397.80)**
		Respiratory effects (slowing respirations to complete apnoea)	79.4% v 0.0%	**RR 75.92 (4.81, 1198.55)**
Shoaib 2009 [49]	100 women (severe PE). LD only (4 g IV and 10 g IM LD) (n = 50) v Pritchard’s IM regimen (4 g IV and 10 g IM LD; 5 g/4 h IM) (n = 50)	Death; respiratory failure or distress; cardiac arrest	0.0% v 0.0%	NA
		Nausea and vomiting	10.0% v 34.0%	**RR 0.29 (0.12, 0.74)**
		Warmth and flushing	70.0% v 80.0%	RR 0.88 (0.70, 1.10)
		Dizziness	20.0% v 56.0%	**RR 0.36 (0.19, 0.65)**
		Irritation at the injection site	0.0% v 20.0%	**RR 0.05 (0.00, 0.79)**
		Caesarean	12.0% v 30.0%	**RR 0.40 (0.17, 0.95)**
Palmer 2009 [50]	76 women (PE). New protocol (20% solution, separate LD and MD bags) (n = 29) v Old protocol (2-8% solution, same LD and MD bag) (n = 47)	Phlebitis; signs or symptoms of toxicity	0.0% v 0.0%	NA
		Calcium gluconate (for hypocalcaemia)	3.5% v 4.3%	RR 0.81 (0.08, 8.54)
		Errors (failure to reset pump after LD)	0.0% v 4.3%	RR 0.32 (0.02, 6.44)
		Errors (change in drug order)	3.5% v 2.1%	RR 1.62 (0.11, 24.92)
Nassar 2006 [51]	155 women (PTL). 1. Treatment for > 48 hours (n = 78) v 2. Treatment for < 48 hours (n = 77)	≥ 1 adverse effect	30.8% v 15.6%	**OR 2.41 (1.10, 5.26)**
		Discontinuation due to adverse effects	6.4% v 0.0%	OR 11.60 (0.63, 213.47)
		Chest tightness	19.2% v 11.8%	OR 1.80 (0.74, 4.40)
		Visual disturbances	6.4% v 1.3%	OR 5.21 (0.59, 45.63)
		Vulvar oedema	1.3% v 0.0%	OR 3.00 (0.12, 74.79)
		Pulmonary oedema	6.4% v 2.6%	OR 2.57 (0.48, 13.66)
		Ileus	3.8% v 1.3%	OR 3.04 (0.31, 29.89)
		Osteopenia	2.6% v 0.0%	OR 5.07 (0.24, 107.25)
		Hypocalcaemia (< 8.5 mg/dl)	24.6% v 15.6%	OR 1.77 (0.74, 4.21)

The bold effect estimates indicate statistical significance.

Abbreviations: *CI* confidence interval, *E* eclampsia, *g* grams, *h* hours, *IM* intramuscular, *IV* intravenous, *LD* loading dose, *MgSO4* magnesium sulphate, *MD* maintenance dose, *mins* minutes, *OR* odds ratio, *PE* pre-eclampsia, *PTL* preterm labour, *RR* risk ratio.

No significant increase in neuromuscular weakness among women receiving nifedipine as their antihypertensive during magnesium sulphate therapy, compared with women receiving an alternative or no antihypertensive agent, was shown in one retrospective cohort [45] (Table 4). Significantly increased risks of thirst, respiratory problems and minor bleeding, were however observed among women receiving nifedipine, compared with no antihypertensive agent; a significantly increased risk of neuromuscular blockade was observed among women receiving an alternative antihypertensive, compared with nifedipine [45] (Table 4). Whilst two prospective before and after studies showed a significant increase in bleeding time for women receiving magnesium sulphate [40,41] (Table 4), this was not supported by an increased risk of postpartum haemorrhage in the randomised trials (Table 1). Similarly, whilst in one retrospective case control study, pregnant women with pulmonary oedema were significantly more likely to have received magnesium sulphate as compared with women without pulmonary oedema [44] (Table 4), the randomised trials did not support an increased risk of pulmonary oedema overall (Table 1).

In one retrospective study, women receiving magnesium sulphate for greater than 48 hours compared with for less than 48 hours had a significantly increased risk of experiencing more than one adverse effect [51] (Table 5). No significant differences were seen, however, in the risk of discontinuing therapy due to adverse effects, or for any other adverse effects [51] (Table 5). In one non-randomised trial, women allocated to a loading dose only, compared with women receiving Pritchard’s regimen (a loading dose of 4 g IV and 10 g IM, and 5 g IM every four hours as maintenance), were significantly less likely to experience nausea and vomiting, dizziness, irritation at the injection site, and undergo a caesarean section [49] (Table 5). Similar to the randomised trials, a significantly increased risk of pain was experienced among women receiving IM versus IV maintenance therapy [46] (Table 5). Supporting the findings from one randomised trial, no significant differences in adverse effects or medication errors were shown in the historical control study that assessed two different magnesium sulphate solutions using an identical dosage regimen [50] (Table 5).

### Evidence from case series

Thirty-two studies [52-83] (3,276 women), 20 prospective and 12 retrospective in nature, reporting maternal adverse effects were included; the characteristics of the studies and the quality assessment are presented in Additional file 4. Adverse effects have been presented in Table 6 as overall mean and median percentage estimates calculated from individual study results, with the range of percentages reported in the studies also presented. For the quality assessment of case series, we predominately considered participant selection, along with the collection/reporting of adverse effect information, as detailed in Additional file 4.

**Table 6:** Adverse effect estimates from comparative studies with concurrent controls

**Adverse effect**	**Mean (%) or effect**	**Median (%)**	**Range (%)**	**Women**	**Studies**
Death	0.14	0.00	0 to 0.41	285	Adewole 2000* [53]; Ekele 2005* [54]; Pritchard 1984* [55]
Cardiac arrest	0.00	0.00	NA	21	Adewole 2000* [53]
Respiratory arrest	0.41	0.42	0 to 0.82	983	Adewole 2000* [53]; Pritchard 1984* [55]; Raman 1995* [56]
Discontinuation due to adverse effects	9.53	9.52	1.75 to 20.78	532	Adewole 2000* [53]; Elliot 1983^ [57]; Girard 2005* [58]; Harding 1997* [59]; Thapa 2008* [60]
Given calcium gluconate	0.70	0.70	NA	717	Raman 1995* [56]
'Toxicity’	3.17	2.04	0.0 to 8.60	182	Dasari 2010* [61]; Donovan 1980* [62]; Mojadidi 1969* [63]; Tukur 2010* [64]
Need to adjust/skip dose due to adverse effects	15.26	15.26	5.26 to 25.26	114	Ekele 2005* [54]; Getaneh 2010*^ [65]
Respiratory depression	1.67	0.72	0 to 4.76	1363	Adewole 2000* [53]; Ahmed 2004* [66]; Begum 2001* [67]; Digre 1990^ [68]; Ekele 2005* [54]; Hales 1995^ [69]; Harding 1997* [59]; Mojadidi 1969* [63]; Pritchard 1984* [55]; Raman 1995* [56]; Sass 2007* [70]
Absent or reduced deep tendon reflexes	4.75	2.55	0 to 18.05	1789	Aali 2007* [52]; Begum 2001* [67]; Digre 1990^ [68]; Donovan 1980* [62]; Ekele 2005* [54]; Hales 1995^ [69]; Omu 2008* [71]; Pritchard 1984* [55]; Raman 1995* [56]; Sass 2007* [70]
Any adverse effects	13.39	14.29	6.76 to 19.11	826	Adewole 2000* [53]; Elliot 1983^ [57]; Omu 2008* [71]
'Minor side effects’	1.75	1.75	NA	57	Girard 2005* [58]
Hypotension	30.56	30.56	NA	72	Hales 1995^ [69]
Flushing or warmth	52.88	52.88	4.55 to 100	27	Cotton 1984* [72]; Harding 1997* [59]
Nausea and/or vomiting	47.37	38.46	3.66 to 100	373	Cotton 1984* [72]; Digre 1990^ [68]; Elliot 1983^ [57]
Generalised weakness	23.08	23.08	NA	13	Digre 1990^ [68]
Drowsiness or confusion	2.58	2.90	0.28 to 4.55	515	Elliot 1983^ [57]; Harding 1997* [59]; Sass 2007* [70]
Headache	2.90	0.72	0.28 to 7.69	506	Digre 1990^ [68]; Elliot 1983^ [57]; Sass 2007* [70]
Blurred vision	46.30	46.30	0.28 to 92.31	368	Digre 1990^ [68]; Elliot 1983^ [57]
Diplopia	30.77	30.77	NA	13	Digre 1990^ [68]
Photophobia	30.77	30.77	NA	13	Digre 1990^ [68]
Visual signs	76.92	76.92	NA	13	Digre 1990^ [68]
Abnormal visual acuity	38.46	38.46	NA	13	Digre 1990^ [68]
Impaired concentration-confusion	23.08	23.08	NA	13	Digre 1990^ [68]
Cardiac arrhythmias	23.08	23.08	NA	13	Digre 1990^ [68]
Chest pain (and/or need for ECG)	3.90	3.90	0.85 to 6.94	427	Elliot 1983^ [57]; Hales 1995^ [69]
Chest tightness	0.28	0.28	NA	355	Elliot 1983^ [57]
Delayed recovery from anaesthesia	0.14	0.14	NA	717	Raman 1995* [56]
Pulmonary oedema	1.25	1.25	1.13 to 1.36	649	Elliot 1983^ [57]; Yeast 1993*^ [73]
Caesarean	49.68	56.52	32.53 to 60.00	225	Aali 2007* [52]; Getaneh 2010*^ [65]; Pritchard 1984* [55]
Caesarean due to labour induction	48.91	51.85	33.33 to 61.54	109	Aali 2007* [52]; Getaneh 2010*^ [65]; Pritchard 1984* [55]
'Transient nausea, vomiting, headache, flushing and palpitations’	NA	NA	NA	15	Jirapinyo 1990^ [74]
'Magnesium toxicity suspected’	2/49 deaths due to hypertensive disorders of pregnancy were attributed to magnesium				Dasari 2010* [61]
Hospital errors in obstetric patients	146 hospital errors in obstetric patients (3^rd^ most common obstetric drug resulting in patient harm)				Kfuri 2008 ~ [75]
	10 class 2 errors (need for additional treatment/ hospitalisation)				Little 2001 ~ [76]
'Restrictive type of respiratory depression’	Sig ↓ in FVC (L)			18	Bilgin 1994* [77]
Decrease in respiratory function – 'generalised respiratory muscle weakness’	Sig ↓ MIP (cm H2O), MEP (cm H2O), FEV_1_ (L)			10	Herpolsheimer 1991* [78]
'Reduced attention and rapid information processing ability’	Sig ↓ in SSS, PASAT, VAF, DSF scores			15	Ghia 2000^ [79]
Increase in bleeding time ('clinical significance remains to be determined’)	Sig ↑			104	Fuentes 1995*^ [80], Guzin 2010* [81], Yazdani 2004^ [82]
	NS ↑			40	Moghadas 2007^ [83]

Values are presented as mean and median percentage estimates from case series, with the range of percentages reported.

*Women received MgSO4 for pre-eclampsia/eclampsia; ^women received MgSO4 as a tocolytic agent; ~unknown indication for use.

Abbreviations: *AE* adverse effect(s), *DSF* Digits Span Forward (Wechsler Adult Intelligence Scale), *FVC* forced vital capacity, *FEV*_*1*_ forced expiratory volume at 1 second, *L* litres, *MIP* maximal inspiratory pressure, *MEP* maximal expiratory pressure, *NS* not significant, *PASAT* Paced Auditory Serial Addition Tes, *Sig* significant, *SSS* Stanford Sleepiness Scale, *VAF* Verbal Associative Fluency Test, ↑: increase, ↓: reduction.

Adverse effect estimates reported in case series were largely similar to those reported in the randomised trials in Comparison 1 (see Additional file 6). Mean/median percentage estimates of serious outcomes, respiratory arrest, the use of calcium gluconate and discontinuation of therapy due to adverse effects, were slightly higher in the case series. The estimates for the outcome 'any maternal adverse effects’ reported in case series were however notably lower than those in the randomised trials. The case series estimates for nausea and/or vomiting, blurred vision and hypotension were higher than estimates from the randomised trials.

### Evidence from case reports

Seventy-five studies describing a total of 137 case reports of maternal adverse effects were included [84-158] (see Table 7; the more detailed characteristics of cases are presented in Additional file 7).

**Table 7:** Adverse effect from case reports

**Common theme and/or associated adverse effects**	**Studies**
**Iatrogenic overdose (16 studies)**	
Death	Anon 1990 [84]; Cohen 1992 [85]; Richards 1985 [86]
Death or persistent vegetative state	Simpson 2004 (7 cases) [87]
Cardiopulmonary arrest	McCubbin 1981 [88]; McDonnell 2009 [89]; Morisaki 2000 [90]; Rabinerson 1994 [91]; Swartjes 1992 [92]
Cardiac arrest	Cohen 1992 [85]
Respiratory arrest	Bohman 1990 [93]; Cao 1999 [94]; McKenna 2006 [95]; Wax 1995 [96]
“Life-threatening situation”	Bruhwiler 1994 [97]
Coma	Hayashi 2003 [98]
Ventilatory impairment; failure to rouse from general anaesthesia	McDonnell 2010 [99]
Need for additional monitoring	Buettner 2010 (2 cases) [100]
Variety (not death or remaining in a persistent vegetative state)	Simpson 2004 (45 cases) [87]
**Rapid administration (1 study)**	
Cardiac arrest	Richards 1985 [86]
**Unintended epidural or intrathecal administration (4 studies)**	
Bilateral periumbilical pain	Dror 1987 [101]
Inadequate pain relief	Goodman 2006 (2 cases) [102]
Paralysis of lower extremities	Lejuste 1985 [103]; Lewis-Younger 2004 [104]
**Increased risk of adverse effects – neuromuscular junction disorders, myopathies and neuropathologies (8 studies)**	
Weakness and/or temporary paralysis	Bashuk 1990 [105]; Bruner 1990 [106]; Catanzarite 2008 [107]
Muscle pain and damage	Hosono 2001 [108]
Acute respiratory insufficiently; ventilatory failure; respiratory depression	Cohen 1976 [109]; Mueksch 2007 [110]; Robins 2007 [111]
“Magnesium toxicity”	Moriarty 2008 [112]
**Increased risk of adverse effects – renal failure (3 studies)**	
Decreased or absent deep tendon reflexes; prolonged QT interval	Archer 2010 [113]
Muscle weakness	Chan 2008 [114]
Progressive quadriparesis	Nethravathi 2007 [115]
**Drug interactions – agents used in general anaesthesia (10 studies)**	
Cardiac arrest	Saitoh 1994 [116]
Respiratory arrest	Baraka 1984 [117]
Failure to achieve adequate ventilation	Nguyen 2001 [118]
Numb; difficultly moving upper extremities	Fay 1996 [119]
Prolonged neuromuscular blockade	Funai 2010 [120]; Hino 1997 [121]; Kwan 1996 [122]; Sinatra 1985 [123]; Yoshida 2006 (2 cases) [124]; Sloan 2001 [125]
**Drug interactions – other agents (6 studies)**	
Neuromuscular blockade – muscle weakness or paralysis	Ben-Ami 1994 [126]; Snyder 1989 [127]; Wu 2010 [128]
Extreme bradycardia	Pittman 2000 [129]
Severe hypotension	Scardo 1997 [130]; Waisman 1998 (2 cases) [131]
**Unusual/unexpected adverse effects (11 studies)**	
Bilateral, progressive labial swelling (need for caesarean)	Awwad 1994 [132]
Worsened clinical picture of appendicitis and cholecystitis	Basaran 2007 [133]
Impaired lactogenesis	Haldeman 1993 [134]
Severe paralytic ileus	Hill 1985 [135]
Marked osteoporotic change (hips, knees, ankles)	Hung 2005 [136]
Breast engorgement and galactorrhea	Lurie 2002 [137]
Development of central pontine myelinolysis	Riggs 2000 [138]
Urinary tract stone (magnesium ammonium phosphate)	Sameshima 1997 [139]
Hyperkalaemia and hyponatremia (hyporeninemic hypoaldosteronism)	Spital 1991 [140]
Left retinal detachment; partial right detachment	Roberts 1998 [141]
Extensive urticarial rash	Thorp 1989 (2 cases) [142]
**Other adverse effects (16 studies)**	
Severe hypotension	Bourgeois 1986 (2 cases) [143]; Rodis 1987 [144]
Hypothermia	Cardosi 1998 [145]; Rodis 1987 [144]
Bradycardia (39-44/minute)	Hennessy 1999 [146]
Asymptomatic atrial fibrillation (100-150/minute)	Oettinger 1993 [147]
Absent deep tendon reflexes	Pritchard 1979 [148]
Marked weakness; difficulty breathing	Pritchard 1979 [148]
Sleepiness/fatigue; depressed/absent deep tendon reflexes	Herschel 2001 [149]; Tang 2010 [150]
Chest pain; inverted T waves (ECG) (transient subendocardial ischemia)	Sherer 1992 [151]
Pulmonary oedema	Elliot 1979 [157]; Worrell 1992 [158]
Bilateral hand contractures; tetany (serum hypocalcaemia)	Koontz 2004 (2 cases) [152]
Diplopia; malaise; paresthesia; hoarseness; tetany (serum hypocalcaemia)	Mayan 1999 (2 cases) [153]
Hypotension; cyanosis; tetany (serum hypocalcaemia)	Monif 1972 [154]
Chest tightness and pain; prolonged QT interval (serum hypocalcaemia)	Nassar 2007 [155]
Delirium with myoclonus (serum hypocalcaemia)	Ganzenvoort 2002 [156]

Iatrogenic overdoses (16 reports) [84-100] and rapid administration of magnesium sulphate (one report) [86] were associated with a range of serious adverse events, such as respiratory arrest, cardiac arrest, cardiopulmonary arrest and death. Of note was a detailed account of 52 errors associated with magnesium sulphate administration; including seven cases resulting in death, or women remaining in a persistent vegetative state [87]. Additional errors, relating to the epidural or intrathecal administration of magnesium sulphate were associated with pain, inadequate pain relief, or temporary paralysis (four reports) [101-104].

Women at an increased risk of adverse effects were described (11 reports) (105-115), including women with renal failure (three reports) [113-115], and women with neuromuscular junction disorders, myopathies, and neuropathologies (eight reports) [105-112]. For the neurological cases, magnesium sulphate administration, according to recommended regimens, was associated with muscle pain, weakness or temporary paralysis, and associated respiratory problems. The interaction of magnesium sulphate with agents used in general anaesthesia (10 reports) [116-125] and with antihypertensive agents including nifedipine (six reports) [126-131] was associated with varying adverse effects, most commonly muscular weakness or paralysis, and altered respiratory function – associated with prolonged neuromuscular blockade.

A variety of unusual maternal adverse effects, not previously attributed to magnesium sulphate, were described (11 reports) [132-142], including bilateral progressive labial swelling [132], marked osteoporotic change [136], and urinary tract stone formation [139]; in such cases women had received prolonged magnesium sulphate tocolysis therapy. Additional adverse effects, such as hypotension and fatigue as reported in randomised trials, were detailed (16 reports) [143-158]. Of note were five reports where adverse effects such as delirium [156], diplopia [153], tetany and paraesthesia [152-154] associated with serum hypocalcaemia, were described.

### Evidence from patient safety organisations

Whilst the majority of patient safety organisations did not provide free access to their adverse event or equivalent databases, information regarding medication errors and maternal adverse effects associated with antenatal magnesium sulphate was available from the Institute for Safe Medication Practices USA. The Institute provided accessible information in their electronic medication safety newsletters. Three cases of serious iatrogenic overdose were reported in the 12 February 1997 issue [159]. Whilst the women survived, one suffered a respiratory arrest, and one, temporary paralysis of her extremities, ICU admission and the need for prolonged ventilation. Additional cases were described in the 30 June 1999, 15 June 2005, 20 October 2005, and 3 June 2010 issues.

The February 2010 issue of the Patient Safety Newsletter 'Sharing Lessons Leaned’ produced by the Office of Safety and Quality in Health Care, Western Australia, reported 12 incidents involving magnesium sulphate overdose from October 2001 to October 2009, identified through searches of state-wide relevant databases [160]; the consequent maternal adverse effects were not however discussed.

### Women’s experiences – from personal blogs and discussion forum threads

(See Additional file 8 for blog and discussion forum thread sources).

#### Before treatment

Women considered information regarding others’ experiences with magnesium sulphate important, with a particular desire for knowledge regarding adverse effects and their severity; a number of women expressed an explicit wish for “the truth, no matter how bad or scary.” Women expressed expectations of treatment benefits, relating to the different indications for use, and also concern and a sense of fear regarding the possible adverse effects of treatment; one woman exclaimed “What?!! NO!!! ANYTHING BUT MAG!!!”

#### During treatment

Whilst variations in experiences existed, women described a variety of adverse effects, the predominant being a sensation of heat: “MY GOD, THE HEAT! I felt so hot and it came from within.” Other commonly described adverse effects were similar to those frequently reported adverse effects in randomised trials – muscle weakness, lethargy, blurred vision, nausea or vomiting, headaches, confusion, arm discomfort, thirst, feeling sweaty and dizzy. Less common adverse effects women attributed to magnesium sulphate treatment included a failed induction of labour and consequent need for a caesarean section, the delayed onset of lactation, and the development of pulmonary oedema. A number of women described their request or the need for treatment cessation due to adverse effects, with two women highlighting their experience of being “accidentally overdosed.” Many women portrayed their experience as terrifying, revealing a sense of anguish.

#### After treatment

Women expressed relief following treatment cessation, and great concerns regarding the possibility of treatment in future pregnancies: “I pray I never have to do it again.” A number of women felt information regarding the possible adverse effects of treatment was miscommunicated and misleading. Although many women recalled adverse effects of severe intensity, they were generally very thankful, and there was agreement among commenting women that the potential perceived benefits of treatment outweighed discomforts experienced, with magnesium sulphate being described as “a necessary evil.”

## Discussion

It is widely acknowledged that the use of antenatal magnesium sulphate, for the prevention of eclampsia in women with pre-eclampsia and the treatment of women with eclampsia [1-4,9], to delay or prevent preterm birth [5,6], and for neuroprotection of the fetus when given to women at risk of preterm birth [7], may be associated with adverse effects for the mother. The risk of individual adverse effects, and how such adverse effects vary according to different regimens, has however not been clear. Determining this is of great importance given the significant number of women who may be eligible for treatment with magnesium sulphate during pregnancy – with approximately 2-8% of pregnancies complicated by pre-eclampsia [161], and an estimated 1-2% of births being very preterm (before 32 weeks’ gestation) [162].

### Summary of main results

We were able to include 21 randomised trials in this systematic review; 11 comparing magnesium sulphate with placebo or no treatment, and 10 comparing different magnesium sulphate regimens. Whilst 'good’ data on well recognised and easily detectable adverse effects may be potentially available from randomised trials, the often small numbers of participants, who may differ from individuals given the treatment in everyday practice, or the short periods of time that the participants are studied for, can reduce the possibility of unpredictable, rare or delayed adverse effects being observed and reported [12,163,164]. For such reasons, it was considered important to include other study designs. We included 15 non-randomised comparative studies with concurrent controls, 32 case series, and 75 papers describing individual case reports.

Evidence from the randomised trials assessing magnesium sulphate versus placebo or no treatment confirmed the expected higher rates of 'minor’ maternal adverse effects among women receiving treatment, without an increase in major complications (death, cardiac arrest, respiratory arrest). The four randomised trials reporting on 'any adverse effects’ of treatment, showed an absolute risk of 38% (2,521/6,642) for women exposed to antenatal magnesium sulphate compared with 8.5% (567/6,680) for women unexposed. Five randomised trials reported on the need for cessation of treatment due to maternal adverse effects, with significantly more women receiving magnesium sulphate having their therapy stopped compared with women not receiving magnesium sulphate (6.6% versus 2.4%). The most frequently reported adverse effects included warmth or flushing, sweating, and arm discomfort or problems at the IV site.

When considering indication for use and regimens for administration, very few differences were observed between the pre-specified subgroups. Considering indication for use, no significant subgroup interactions were identified, expect for when considering arm discomfort, which suggested a possible increase for women receiving treatment for fetal neuroprotection (compared with women receiving treatment for pre-eclampsia). When considering regimen for administration for the trials comparing magnesium sulphate with placebo or no treatment, the subgroup interaction tests did not indicate differential effects by treatment subgroups. For the majority of outcomes, the subgroups contained very few trials (many contained only one), making comparisons between subgroups difficult.

The 10 randomised trials comparing different magnesium sulphate regimens were mostly too small to provide reliable evidence about the comparative effects of different regimens on maternal adverse effects. Whilst this review did not formally assess efficacy, none of the trials demonstrated significantly decreased effectiveness with the lower dose regimens. One trial reporting on the cessation of treatment due to adverse effects showed significantly more women receiving higher dose IM maintenance having their therapy ceased as compared with women receiving lower dose IM maintenance (25.9% versus 1.4%). This was supported by one small non-randomised trial, reporting significantly higher rates of adverse effects for women receiving Pritchard’s IM regimen versus a loading dose only. One randomised trial showed significantly more women receiving magnesium sulphate via an IV pump reporting 'acceptable’ pain as compared with women receiving magnesium sulphate via the IM route (100% versus 20%). This was supported by a non-randomised trial that showed a significantly higher risk of pain among women receiving magnesium sulphate via the IM route versus via an IV infusion.

The data from the 32 case series largely supported that from the randomised trials. Whilst serious outcome estimates were broadly similar, differences between procedures for collecting information on adverse events between studies may help to explain the variations observed between estimates. Higher estimates for 'any adverse effects’ in randomised trials may have been influenced by the use of more rigorous methods for data collection, such as the use of trial-specific check-lists [19]. The notably higher estimates in case series for blurred vision and nausea and/or vomiting similarly may have been influenced by the use of specific questioning and interviewing in regards to adverse effects [68], and incomplete reporting, such as the use of generic statements [72].

As the establishment of a causal relationship between a treatment and a subsequent adverse effect through individual cases is difficult, data presented in case reports should be interpreted with a degree of caution. That said, the significant harm that may result from accidental overdose of magnesium sulphate is unquestionable. The 16 reports, including Simpson’s account of 52 errors associated with administration [87], highlight the potentially life-threatening consequences of magnesium sulphate overdose.

Factors contributing to iatrogenic overdoses were identified [87]. These included: the inaccurate or inadequate mixing of an IV magnesium sulphate solution, leading to a higher concentration of solution than intended or an undesirable concentration gradient between the infusion bag and tubing; the use of infusion bags containing a high total dose of magnesium sulphate, and the consequent danger associated with infusion rate programming error; the use of the same infusion bag for administration of the loading and maintenance infusions, and the failure to reduce the infusion rate following the loading dose; the removal of an IV line from an IV pump temporarily, and the accidental free-flow of solution; the handover of clinical responsibilities in busy units, and the transfer of women between units. Fortunately in most reported cases, the error was recognised before permanent adverse outcomes occurred. Monitoring of maternal status (before, during and after administration of magnesium sulphate), to allow signs and symptoms of toxicity to be recognised, and for potential consequences of errors to be mitigated in a timely manner, is imperative. Common recommendations for monitoring include: regular assessment and documentation of vital signs (pulse, blood pressure, respiratory rate), level of consciousness, patellar reflexes and urinary output; and the provision of 1:1 nursing care during loading administration (and 1:2-3 care during maintenance or where 1:1 care is not possible) [87].

Case reports also highlighted the potential for an increased risk of adverse effect occurrence among particular groups of women – those with renal insufficiency or failure, and those with neuromuscular disorders such as myasthenia gravis, Friedreich’s ataxia, myotonic dystrophy, and other rare myopathies, such as mitochondrial myopathy. The potential for prolonged/enhanced neuromuscular blockade when magnesium sulphate is used in conjunction with general anaesthetic agents and antihypertensive agents including nifedipine was also shown. Whilst this potential interaction between magnesium sulphate and nifedipine was highlighted, reassuringly no increased neuromuscular blockade was shown in the retrospective cohort study that compared the use of nifedipine versus other/no antihypertensive during magnesium sulphate treatment (Table 4). Though an excess of respiratory problems and minor bleeding was shown among women receiving nifedipine, compared with no antihypertensive, the condition of women receiving nifedipine was considered comparatively more severe (more severe hypertension, more frequent pre-eclampsia symptoms), which was suggested to account for this difference [45].

Adverse effects not previously reported in randomised trials that were discussed in case reports included marked bilateral labial swelling, severe paralytic ileus, marked osteoporotic change, and urinary tract stone formation [132,135,136,139]. In each case, magnesium sulphate had been used as a tocolytic agent for a prolonged period (nine, three, 101 and 21 days respectively). The relevant Cochrane reviews have shown magnesium sulphate to be ineffective at delaying or preventing preterm birth [5,6]. Acknowledging this and the maternal adverse effects of magnesium sulphate, from minor to unpleasant to potentially fatal, its use as a tocolytic appears inappropriate, as has been strongly stated by Grimes and Nanda [165]. Furthermore, the Food and Drug Administration in the United States recently advised health care professionals against the use of magnesium sulphate as a tocolytic for more than five to seven days due to associated harm to developing fetal bones (the use of the drug for tocolysis is 'off-label’ (not approved by the Food and Drug Administration)) [166]. In view of the extremely widespread use of antenatal magnesium sulphate in obstetric practice, the potential neonatal and infant adverse effects of antenatally administered magnesium sulphate additionally require comprehensive evaluation, such as by systematic review.

### Overall completeness, applicability and quality of the evidence

This review was based on a comprehensive search strategy, and whilst no language restrictions were applied, inclusion was restricted to those studies written in English or for which a translation was readily available, and to those studies published in the databases that were searched, which may have limited available studies. Additionally, there were a number of articles that were excluded as we were unable to obtain the abstract and/or full-text. The included studies were however conducted in both high-income and low-income countries, and are therefore may considered widely applicable to the treatment of pregnant women.

The main limitations include the small number of studies with relatively small sample sizes comparing different antenatal magnesium sulphate regimens, and the missing data for several important outcomes in almost all trials. Additionally, a great number of trials could not be included in this review, as they did not provide any information regarding maternal adverse effects of treatment. This supports previous reports, that many trials do not report harms, or do so in a fragmented or suboptimal way [167].

The studies included in the review were of mixed quality and we emphasise the need to consider the risk of bias as outlined in Figures 2, 3, 4, 5 and 6, and the more detailed quality assessment in Additional file 4, which includes consideration of the different procedures used to collect information on adverse effects and reporting, when interpreting results.

### Potential biases in the review process

We recognise the potential for bias associated with the second reviewer reviewing a random sample of the search records (10% of the total), and independently extracting data from a random sample of included studies (10% of the total, and all 21 randomised controlled trials). We attempted to minimise bias however in a number of ways, for example by using a comprehensive search strategy. Although this was extensive, it is possible that some studies conducted in low- and middle-income countries may not have been identified, if they were not published, or published in journals not indexed in the bibliographic databases searched.

### Agreements and disagreements with other reviews

We are not aware of any other reviews specifically assessing maternal adverse effects of antenatal magnesium sulphate, and comparing the adverse effects of different regimens for the various indications for use. The findings of this review are broadly consistent with results from relevant Cochrane reviews, comparing magnesium sulphate with alternative drugs for women with eclampsia [1-3], evaluating magnesium sulphate for women with pre-eclampsia [4], assessing magnesium sulphate for preventing preterm birth in and after threatened preterm labour [5,6], and evaluating magnesium sulphate for neuroprotection of the fetus for women at risk of preterm birth [7].

These Cochrane reviews similarly concluded no increased risk of major maternal complications (death, cardiac arrest, respiratory arrest) for women receiving magnesium sulphate according to those regimens used in the trials [4,5,7], increased risks of many comparatively minor adverse effects [4,6,7], and an increased risk of women ceasing treatment due to adverse effects [7]. One Cochrane review that assessed magnesium sulphate for women with pre-eclampsia had previously shown a small increased risk of caesarean section for women receiving magnesium sulphate, compared with placebo/no treatment [4], as was suggested in this review.

The findings of this review largely support the methodological conclusions from the systematic review by Golder et al. [10] – that there are often limited differences in the risk estimates of adverse effects derived from randomised trials and observational studies. Further, they highlight the importance of including a broad range of study designs to ensure that the most complete picture of potential harms associated with an intervention can be drawn [10,163,167].

## Conclusions

### Implications for practice

By considering adverse effects alone, this review was not designed to guide the choice of magnesium sulphate regimen for the different antenatal indications for use. Healthcare providers must therefore weigh the potential risks of magnesium sulphate for the mother against the benefits, for each known beneficial indication separately.

Vigilance in the use of magnesium sulphate is required to promote and ensure safety for women. Certainly, errors associated with the administration of magnesium sulphate represent a significant and perhaps unappreciated risk of harm. An important step in improving the safety for women is knowledge and recognition of the risk of error; this review, along with articles such as that published by Simpson [87], may help in increasing awareness. Individual hospitals should consider common precursors, or contributing factors, to errors associated with antenatal magnesium sulphate occurring in their obstetric units, and ensure safety procedures are in place to help prevent such accidents.

It is important, where ever possible, that women receive a full explanation, not only of why antenatal magnesium sulphate is in their case needed, but also of the nature of symptoms that may be experienced during treatment. An understanding of these benefits, the possible adverse effects, and the vigilant care and monitoring that will be received during treatment, may help to decrease or relieve anxiety during treatment that may otherwise be caused by unexpected adverse effects. Health professionals should seek to make their approach to information provision open and honest.

### Implications for research

Fewer maternal adverse effects may be a benefit of lower dose magnesium sulphate regimens, and whilst this review did not formally assess effectiveness, no trial included in this review showed compromised efficacy with such lower dose regimens. Further trials comparing different regimens are however required, to determine the optimal regimens that achieve the desired clinical effectiveness with minimal maternal adverse effects and cessation, for each of the known beneficial indications for use. Such trials must be of a high quality, and of sufficient sample size to assess the comparative effects on relevant maternal and/or infant efficacy and safety related outcomes. For all future randomised trials, consideration of the extension for harms of the CONSORT statement [15] including during the study design phase, is crucial to ensure the better collection and reporting of adverse effects [167].

A number of important questions remain regarding maternal adverse effects of magnesium sulphate. Common reasons for ceasing treatment, and how serious adverse effects and treatment cessation vary according to total dose of magnesium sulphate received, require evaluation. Such questions may be addressed in an individual patient data meta-analysis. Additional well-designed and sufficiently powered trials or observational studies are required to address the need to reduce errors associated with magnesium sulphate administration. Such studies may, for example, consider comparing the use of a pre-mixed solution with a concentrate requiring dilution, or the use of separate infusion bags for the loading and maintenance dose infusions, compared with the use of the same infusion bag with the need to reprogram infusion rates.

## Abbreviations

CI: Confidence interval; ICU: Intensive care unit; IM: Intramuscular; IV: Intravenous; OR: Odds ratio; RR: Risk ratio.

## Competing interests

CAC was the principal investigator for the Australasian Collaborative Trial of Magnesium Sulphate. Two review authors (CAC and PFM) were co-authors of the Cochrane review 'Magnesium sulphate for women at risk of preterm birth for neuroprotection of the fetus’. The three review authors (CAC, ESB and PFM) were authors of the Cochrane review 'Different magnesium sulphate regimens for neuroprotection of the fetus’. CAC was an author of the Cochrane reviews 'Magnesium sulphate for preventing preterm birth in threatened preterm labour’ and 'Magnesium maintenance therapy for preventing preterm birth after threatened preterm labour’.

## Authors’ contributions

ESB, CAC and PFM designed the study. ESB and PFM undertook searches, extracted and analysed data. ESB wrote the first draft of the paper. All authors supported the interpretation of results, provided comments on subsequent drafts and approved the final version.

## Pre-publication history

The pre-publication history for this paper can be accessed here:

http://www.biomedcentral.com/1471-2393/13/195/prepub

## Supplementary Material

Additional file 1 PRISMA checklist. Click here for file

Additional file 2 Search strategies. Click here for file

Additional file 3 References to included studies. Click here for file

Additional file 4 Characteristics of included studies. Click here for file

Additional file 5 Forest plots of Comparisons 1–5. Click here for file

Additional file 6 Comparison of case series and randomised trial adverse effect estimates. Click here for file

Additional file 7 Detail of adverse effects from case reports. Click here for file

Additional file 8 Blog and discussion forum thread sources. Click here for file
