# Supplementary material for: Maternal adverse effects of different antenatal magnesium sulphate regimens for improving maternal and infant outcomes: a systematic review
Source: BMC Pregnancy Childbirth. 2013 Oct 21;13:195. doi: 10.1186/1471-2393-13-195 (PMC4015216; doi:10.1186/1471-2393-13-195)
Supplement: Additional file 2 — Search strategies. [file 1471-2393-13-195-S2.pdf]

Search strategies.

Ovid/**MEDLINE** (1948 - week 4 July 2012)
Ovid/**EMBASE** (1980 – week 4 July 2012)

Date searched: 10/07/12

The following search strategy retrieved 4082 records (EMBASE: 2477, MEDLINE: 1690)

1. exp Magnesium Sulfate/
2. magnesium adj sulfate.mp.
3. magnesium adj sulphate.mp.
4. MgSO4.mp.
5. 1 or 2 or 3 or 4
6. exp Pregnancy/
7. exp Pregnancy Complications/
8. exp Prenatal Care/
9. exp Perinatal Care/
10. pregnan$.mp.
11. (labor or laboring).mp.
12. labour$.mp.
13. antepart$.mp.
14. prenatal$.mp.
15. antenatal$.mp.
16. perinatal$.mp.
17. intranatal$.mp.
18. obstetric$.mp.
19. intrapart$.mp.
20. preterm.mp.
21. tocoly$.mp.
22. maintenance adj therapy.mp.
23. preeclamp$.mp.
24. pre adj eclamp$.mp.
25. eclamp$.mp.
26. neuroprotection.mp.
27. cerebral adj palsy.mp.
28. or/6-27
29. 5 and 28
30. exp Animals/
31. exp Humans/
32. 30 not 31
33. 29 not 32
34. remove duplicates from 33

Cochrane Library (CENTRAL) (inception - July 2012)

Date searched: 11/07/12

The following search strategy retrieved 504 records

1 MeSH descriptor Magnesium Sulfate explode all trees
2 magnesium next sulfate in All Fields in all products
3 magnesium next sulphate in All Fields in all products
4 MgSO4 in All Fields in all products
5 (#1 OR #2 OR #3 OR #4)
6 MeSH descriptor Pregnancy explode all trees
7 MeSH descriptor Pregnancy Complications explode all trees
8 MeSH descriptor Perinatal Care explode all trees
9 MeSH descriptor Prenatal Care explode all trees
10 pregnan* in All Fields in all product
11 birth or childbirth in All Fields in all products
12 labor or laboring in All Fields in all products
13 labour* in All Fields in all products
14 antepart* in All Fields in all products
15 prenatal* in All Fields in all products
16 antenatal* in All Fields in all products
17 perinatal* in All Fields in all products
18 intrapart* in All Fields in all products
19 intranatal* in All Fields in all products
20 obstetric* in All Fields in all products
21 preterm in All Fields in all products
22 matern* in All Fields in all product
23 tocoly* in All Fields in all products
24 preeclamp* in All Fields in all products
25 pre next eclamp* in All Fields in all products
26 eclamp* in All Fields in all products
27 maintenance next therapy in All Fields in all products
28 neuroprotection in All Fields in all products
29 cerebral next palsy in All Fields in all products
30 (#6 OR #7 OR #8 OR #9 OR #10 OR #11 OR #12 OR #13 OR #14 OR #15 OR #16 OR #17 OR #18 OR #19 OR #20 OR #21 OR #22 OR #23 OR #24 OR #25 OR #26 OR #27 OR #28 OR # 29)
31 (#5 AND #30)

TOXLINE (1965 - July 2012)

Date searched: 11/07/12

The following search strategy retrieved 391 records

("magnesium sulphate" OR "magnesium sulphate" OR MgSO4) AND (pregnan* OR labor OR labouring OR labour* OR antepart* OR prenatal* OR antenatal* OR perinatal* OR intranatal* OR obstetric* OR preterm OR matern* OR tocol* OR preeclamp* OR "pre-eclamp*" OR "pre eclamp*" OR eclamp* OR "maintenance therapy" OR neuroprotection OR "cerebral palsy")
