# Supplementary material for: Maternal adverse effects of different antenatal magnesium sulphate regimens for improving maternal and infant outcomes: a systematic review
Source: BMC Pregnancy Childbirth. 2013 Oct 21;13:195. doi: 10.1186/1471-2393-13-195 (PMC4015216; doi:10.1186/1471-2393-13-195)
Supplement: Additional file 3 — References to included studies. [file 1471-2393-13-195-S3.pdf]

References to included studies.

1. Chen FP, Chang SD, Chu KK (1995) Expectant management in severe preeclampsia: does magnesium sulfate prevent the development of eclampsia? Acta Obstet Gynecol Scand 74: 181-185.
2. Coetzee EJ, Dommisse J, Anthony J (1998) A randomised controlled trial of intravenous magnesium sulphate versus placebo in the management of women with severe pre-eclampsia. Br J Obstet Gynaecol 105: 300-303.
3. Cox SM, Sherman ML, Leveno KJ (1990) Randomized investigation of magnesium sulfate for prevention of preterm birth. Am J Obstet Gynecol 163: 767-772.
4. Crowther CA, Hiller JE, Doyle LW, Haslam RR, Australasian Collaborative Trial of Magnesium Sulphate Collaborative Group (2003) Effect of magnesium sulfate given for neuroprotection before preterm birth: a randomized controlled trial. JAMA 290: 2669-2676.
5. The Magpie Trial Collaborative Group (2002) Do women with pre-eclampsia, and their babies, benefit from magnesium sulphate? The Magpie Trial: A randomised placebo-controlled trial. Lancet 359: 1877-1890.
6. Livingston JC, Livingston LW, Ramsey R, Mabie BC, Sibai BM (2003) Magnesium sulfate in women with mild preeclampsia: a randomized controlled trial. Obstet Gynecol 101: 217-220.
7. Ma L (1992) [Magnesium sulfate in prevention of preterm labor]. Zhong hua yi xue za zhi 72: 158-161.
8. Marret S, Marpeau L, Zupan-Simunek V, Eurin D, Leveque C, et al. (2007) Magnesium sulfate given before very-preterm birth to protect infant brain: the randomized controlled PREMAG trial. Br J Obstet Gynaecol 114: 310-318.
9. Moodley J, Moodley VV (1994) Prophylactic anticonvulsant therapy in hypertensive crises of pregnancy the need for a large, randomized trial. Hypertens Pregnancy: 245-252.
10. Rouse DJ, Hirtz DG, Thom E, Varner MW, Spong CY, et al. (2008) A randomized, controlled trial of magnesium sulfate for the prevention of cerebral palsy. N Engl J Med 359: 895-905.
11. Witlin AG, Friedman SA, Sibai BM (1997) The effect of magnesium sulfate therapy on the duration of labor in women with mild preeclampsia at term: a randomized, double-blind, placebo-controlled trial. Am J Obstet Gynecol 176: 623-627.
12. Malapaka SVN, Ballal PK (2011) Low-dose magnesium sulfate versus Pritchard regimen for the treatment of eclampsia imminent eclampsia. Int J Gynaecol Obstet: In press.
13. Shilva, Saha SC, Kalra J, Prasad R (2007) Safety and efficacy of low-dose MgSO4 in the treatment of eclampsia. Int J Gynaecol Obstet 97: 150-151.
14. Behrad V, Moossavifar N, Mojtahedzadeh M, Esmaili H, Moghtadeii P (2003) A prospective, randomized, controlled trial of high and low doses of magnesium sulfate for acute tocolysis. Acta Medica Iranica: 126-131.
15. Terrone DA, Rinehart BK, Kimmel ES, May WL, Larmon JE, et al. (2000) A prospective, randomized, controlled trial of high and low maintenance doses of magnesium sulfate for acute tocolysis. Am J Obstet Gynecol 182: 1477-1482.
16. Bhattacharjee N, Saha SP, Ganguly RP, Patra KK, Dhali B, et al. (2011) A randomised comparative study between low-dose intravenous magnesium sulphate and standard intramuscular regimen for treatment of eclampsia. J Obstet Gynaecol 31: 298-303.
17. Chissell S, Botha JH, Moodley J, McFadyen L (1994) Intravenous and intramuscular magnesium sulphate regimens in severe pre-eclampsia. S Afr Med J 84: 607-610.
18. Mundle S, Regi A, Biswas B, Bracken H, Easterling T, et al. (2009) Preeclampsia in low-resource settings: a randomized trial of IV MgSO4 via flow controlled pump. Int J Gynaecol Obstet 107: S278.
19. Zygmunt M, Heilmann L, Berg C, Wallwiener D, Grischke E, et al. (2003) Local and systemic tolerability of magnesium sulphate for tocolysis. Eur J Obstet Gynecol Reprod Biol 107: 168-175.
20. Ehrenberg HM, Mercer BM (2006) Abbreviated postpartum magnesium sulfate therapy for women with mild preeclampsia: a randomized controlled trial. Obstet Gynecol 108: 833-838
21. Suneja A, Sinha S, Vaid N, Ahuja S (2008) A prospective randomized controlled trial to individualize the duration of post partum magnesium sulfate therapy. Hypertens Pregnancy 27: 504.

22. Chowdhury JR, Chaudhuri S, Bhattacharyya N, Biswas PK, Panpalia M (2009) Comparison of intramuscular magnesium sulfate with low dose intravenous magnesium sulfate regimen for treatment of eclampsia. J Obstet Gynaecol Res 35: 119-125.
23. Mahajan NN, Thomas A, Soni RN, Gaikwad NL, Jain SM (2009) 'Padhar regime' - a low-dose magnesium sulphate treatment for eclampsia. Gynecol Obstet Invest 67: 20-24.
24. Shoaib T, Khan S, Javed I, Bhutta SZ (2009) Loading dose of magnesium sulphate versus standard regime for prophylaxis of pre-eclampsia. J Coll Physicians Surg Pak 19: 30-33.
25. Young BK, Weinstein HM (1977) Effects of magnesium sulfate on toxemic patients in labor. Obstet Gynecol 49: 681-685.
26. Ales KL, Charlson ME (1987) Prognosis of hypertension first documented during labor. Am J Perinatol 4: 317-323.
27. Assaley J, Baron JM, Cibils LA (1998) Effects of magnesium sulfate infusion upon clotting parameters in patients with pre-eclampsia. J Perinat Med 26: 115-119.
28. Kynczl-Leisure M, Cibils LA (1996) Increased bleeding time after magnesium sulfate infusion. Am J Obstet Gynecol 175: 1293-1294.
29. Magee LA, Miremadi S, Li J, Cheng C, Ensom MHH, et al. (2005) Therapy with both magnesium sulfate and nifedipine does not increase the risk of serious magnesium-related maternal side effects in women with preeclampsia. Am J Obstet Gynecol 193: 153-163.
30. Nassar AH, Sakhel K, Maarouf H, Naassan GR, Usta IM (2006) Adverse maternal and neonatal outcome of prolonged course of magnesium sulfate tocolysis. Acta Obstet Gynecol Scand 85: 1099-1103.
31. Palmer L, Newby BD (2009) Development of a simplified protocol for administration of 20% magnesium sulphate for prophylaxis and treatment of eclampsia. Can J Hosp Pharm 62: 490-495.
32. Park KH, Cho YK, Lee CM, Choi H, Kim BR, et al. (2006) Effect of preeclampsia, magnesium sulfate prophylaxis, and maternal weight on labor induction: a retrospective analysis. Gynecol Obstet Invest 61: 40-44.
33. Poggi SH, Barr S, Cannum R, Collea JV, Landy HJ, et al. (2003) Risk factors for pulmonary edema in triplet pregnancies. J Perinatol 23: 462-465.
34. Ramanathan J, Sibai BM, Duggirala V, Maduska AL (1988) Pulmonary function in preeclamptic women receiving MgSO4. J Reprod Med 33: 432-435.
35. Ramanathan J, Sibai BM, Pillai R, Angel JJ (1988) Neuromuscular transmission studies in preeclamptic women receiving magnesium sulfate. Am J Obstet Gynecol 158: 40-46.
36. Seyb ST, Berka RJ, Socol ML, Dooley SL (1999) Risk of cesarean delivery with elective induction of labor at term in nulliparous women. Obstet Gynecol 94: 600-607.
37. Aali BS, Khazaeli P, Ghasemi F (2007) Ionized and total magnesium concentration in patients with severe preeclampsia-eclampsia undergoing magnesium sulfate therapy. J Obstet Gynaecol Res 33: 138-143.
38. Adewole IF, Oladokun A, Okewole AI, Omigbodun AO, Afolabi A, et al. (2000) Magnesium sulphate for treatment of eclampsia: the Nigerian experience. Afr J Med Med Sci 29: 239-241.
39. Ahmed R (2004) Magnesium sulphate as an anticonvulsant in the management of eclampsia. J Coll Physicians Surg Pak 14: 605-607.
40. Begum R, Begum A, Johanson R, Ali MN, Akhter S (2001) A low dose ("Dhaka") magnesium sulphate regime for eclampsia. Acta Obstet Gynecol Scand 80: 998-1002.
41. Bilgin T, Cengiz C, Ozan H (1994) Alterations in respiratory functions during magnesium sulfate infusion in severe preeclampsia and eclampsia. Int J Gynaecol Obstet 45: 59-60.
42. Cotton DB, Gonik B, Dorman KF (1984) Cardiovascular alterations in severe pregnancy-induced hypertension: acute effects of intravenous magnesium sulfate. Am J Obstet Gynecol 148: 162-165.
43. Dasari P, Habeebullah S (2010) Maternal mortality due to hypertensive disorders of pregnancy in a tertiary care center in Southern India. Int J Gynaecol Obstet 110: 271-273.
44. Digre KB, Varner MW, Schiffman JS (1990) Neuroophthalmologic effects of intravenous magnesium sulfate. Am J Obstet Gynecol 163: 1848-1852.
45. Donovan EF, Tsang RC, Steichen JJ, Strub RJ, Chen IW, et al. (1980) Neonatal hypermagnesemia: effect on parathyroid hormone and calcium homeostasis. J Pediatr 96: 305-310.

46. Ekele BA, Badung SLH (2005) Is serum magnesium estimate necessary in patients with eclampsia on magnesium sulphate? Afr J Reprod Health 9: 128-132.
47. Elliott JP (1983) Magnesium sulfate as a tocolytic agent. Am J Obstet Gynecol 147: 277-284.
48. Fuentes A, Rojas A, Porter KB, Saviello G, O'Brien WF (1995) The effect of magnesium sulfate on bleeding time in pregnancy. Am J Obstet Gynecol 173: 1246-1249.
49. Getaneh W, Kumbi S (2010) Use of magnesium sulfate in pre-eclampsia and eclampsia in teaching hospitals in Addis Ababa: a practice audit. Ethiop Med J 48: 157-164.
50. Ghia N, Spong CY, Starbuck VN, Scialli AR, Ghidini A (2000) Magnesium sulfate therapy affects attention and working memory in patients undergoing preterm labor. Am J Obstet Gynecol 183: 940-944.
51. Girard B, Beucher G, Muris C, Simonet T, Dreyfus M (2005) [Magnesium sulphate and severe preeclampsia: its use in current practice]. J Gynecol Obstet Biol Reprod 34: 17-22.
52. Guzin K, Goynumer G, Gokdagli F, Turkgeldi E, Gunduz G, et al. (2010) The effect of magnesium sulfate treatment on blood biochemistry and bleeding time in patients with severe preeclampsia. J Matern Fetal Neonatal Med 23: 399-402.
53. Hales KA, Matthews JP, Rayburn WF, Atkinson BD (1995) Intravenous magnesium sulfate for premature labor: comparison between twin and singleton gestations. Am J Perinatol 12: 7-10.
54. Harding K, Erskine K, Howell P (1997) Audit of the impact of magnesium sulphate on the management of severe pre-eclampsia in a London university hospital. Eur J Anaesthesiol 14 (5): 527-528.
55. Herpolsheimer A, Brady K, Yancey MK, Pandian M, Duff P (1991) Pulmonary function of preeclamptic women receiving intravenous magnesium sulfate seizure prophylaxis. Obstet Gynecol 78: 241-244.
56. Jirapinyo M, Thuvasethakul P, Leelaphiwat S (1990) Prospective study on premature labor with magnesium sulfate. Asia Oceania J Obstet Gynaecol 16: 91-96.
57. Kfuri TA, Morlock L, Hicks RW, Shore AD (2008) Medication errors in obstetrics. Clin Perinatol 35: 101-117.
58. Little JA, Velazquez MB, Rayburn WF (2003) Reported medication errors in obstetric inpatients in 1 hospital. J Reprod Med 48: 818-820.
59. Moghadas FRS, Motevalian M, Rezai Z, Ehteshamipour E (2007) The effect of magnesium sulfate therapy on bleeding time in women with threatened preterm labor. Iranian Journal of Pharmacology and Therapeutics 6: 55-57.
60. Mojadidi Q, Thompson RJ (1973) Five years' experience with eclampsia. South Med J 66: 414-416.
61. Omu AE, Al-Harmi J, Vedi HL, Mlechkova L, Sayed AF, et al. (2008) Magnesium sulphate therapy in women with pre-eclampsia and eclampsia in Kuwait. Med Princ Pract 17: 227-232.
62. Pritchard JA, Cunningham FG, Pritchard SA (1984) The Parkland Memorial Hospital protocol for treatment of eclampsia: evaluation of 245 cases. Am J Obstet Gynecol 148: 951-963.
63. Raman NV, Rao CA (1995) Magnesium sulfate as an anticonvulsant in eclampsia. Int J Gynaecol Obstet 49: 289-298.
64. Sass N, Mesquita M, Kenji G, Goncalves F, Arauj F, et al. (2007) Do women with preeclampsia benefit from an exclusively intravenous magnesium sulphate regimen? Is 2.0 grams/hour effective? (abstract). Hypertens Pregnancy 4: S591.
65. Thapa K, Jha R (2008) Magnesium sulphate: a life saving drug. J Nepal Med Assoc 47: 104-108.
66. Tukur J, Muhammad Z (2010) Management of eclampsia at AKTH: before and after magnesium sulphate. Niger J Med 19: 104-107.
67. Yazdani M, Jahvani FA, Tabei SZ (2004) Effects of magnesium sulfate on bleeding time in premature labor. Iranian Journal of Medical Sciences 29 (4): 172-174.
68. Yeast JD, Halberstadt C, Meyer BA, Cohen GR, Thorp JA (1993) The risk of pulmonary edema and colloid osmotic pressure changes during magnesium sulfate infusion. Am J Obstet Gynecol 169: 1566-1571.
69. Anon (1990) Magnesium overdose kills pregnant R.Ph.; her twins saved. Drug Top 134: 11-12.
70. Cohen MR, Davis NM (1992) Free flow associated with electronic infusion devices: underestimated danger. Hosp Pharm 27: 384-390.
71. Richards A, Stather-Dunn L, Moodley J (1985) Cardiopulmonary arrest after the administration of magnesium sulphate. A case report. S Afr Med J 67: 145.

72. Simpson KR, Knox GE (2004) Obstetrical accidents involving intravenous magnesium sulfate: recommendations to promote patient safety. MCN Am J Matern Child Nurs 29: 161-169.
73. McCubbin JM, Sibai BM, Ardella TN, Anderson GD (1981) Cardiopulmonary arrest due to acute maternal hypermagnesaemia. Lancet 1: 1058.
74. McDonnell NJ (2009) Cardiopulmonary arrest in pregnancy: two case reports of successful outcomes in association with perimortem Caesarean delivery. British Journal of Anaesthesia 103: 406-409.
75. Morisaki H, Yamamoto S, Morita Y, Kotake Y, Ochiai R, et al. (2000) Hypermagnesemia-induced cardiopulmonary arrest before induction of anesthesia for emergency cesarean section. J Clin Anesth 12: 224-226.
76. Rabinerson D, Gruber A, Kaplan B, Royburt M, Ovadia J (1994) Accidental cardiopulmonary arrest following magnesium sulphate overdose. Eur J Obstet Gynecol Reprod Biol 55: 149-150.
77. Swartjes JM, Schutte MF, Bleker OP (1992) Management of eclampsia: cardiopulmonary arrest resulting from magnesium sulfate overdose. Eur J Obstet Gynecol Reprod Biol 47: 73-75.
78. Bohman VR, Cotton DB (1990) Supralethal magnesemia with patient survival. Obstet Gynecol 76: 984-986.
79. Cao Z, Bideau R, Valdes R, Jr., Elin RJ (1999) Acute hypermagnesemia and respiratory arrest following infusion of MgSO4 for tocolysis. Clin Chim Acta 285: 191-193.
80. McKenna D (2006) That's our drip counter! Lancet 367: 1873.
81. Wax JR, Segna RA, Vandersloot JA (1995) Magnesium toxicity and resuscitation--an unusual cause of postcesarean evisceration. Int J Gynaecol Obstet 48: 213-214.
82. Bruhwiler H, Hafliger M, Luscher KP (1994) [Severe accidental magnesium poisoning in a twins pregnancy in the 32nd week of pregnancy]. Geburtshilfe und Frauenheilkunde 54: 184-186.
83. Hayashi K, Oshiro M, Takara I, Iha H, Sugahara K (2003) [Coma caused by hypermagnesemia in a pregnant woman complicated with HELLP syndrome]. Masui 52: 783-785.
84. McDonnell NJ, Muchatuta NA, Paech MJ (2010) Acute magnesium toxicity in an obstetric patient undergoing general anaesthesia for caesarean delivery. Int J Obstet Anesth 19: 226-231.
85. Buettner AU (2011) Two cases of inadvertent magnesium sulphate overdose. Int J Obstet Anesth 20: 92-93.
86. Dror A, Henriksen E (1987) Accidental epidural magnesium sulfate injection. Anesth Analg 66: 1020-1021.
87. Goodman EJ, Haas AJ, Kantor GS (2006) Inadvertent administration of magnesium sulfate through the epidural catheter: report and analysis of a drug error. Int J Obstet Anesth 15: 63-67.
88. Lejuste MJ (1985) Inadvertant intrathecal administration of magnesium sulfate. S Afr Med J 68: 367-368.
89. Lewis-Younger C, Speranza V, Gaar G (2004) Temporary paralysis resulting from medical error. J Toxicol Clin Toxicol 42: 732.
90. Bashuk RG, Krendel DA (1990) Myasthenia gravis presenting as weakness after magnesium administration. Muscle Nerve 13: 708-712.
91. Bruner JP, Yeast JD (1990) Pregnancy associated with Friedreich ataxia. Obstet Gynecol 76: 976-977.
92. Catanzarite V, Gambling D, Bird LM, Honold J, Perkins E (2008) Respiratory compromise after MgSO4 therapy for preterm labor in a woman with myotonic dystrophy: a case report. J Reprod Med 53: 220-222.
93. Hosono T, Suzuki M, Chiba Y (2001) Contraindication of magnesium sulfate in a pregnancy complicated with late-onset diabetes mellitus and sensory deafness due to mitochondrial myopathy. J Matern Fetal Med 10: 355-356.
94. Cohen BA, London RS, Goldstein PJ (1976) Myasthenia gravis and preeclampsia. Obstet Gynecol 48: 35S-37S.
95. Mueksch JN, Stevens WA (2007) Undiagnosed myasthenia gravis masquerading as eclampsia. Int J Obstet Anesth 16: 379-382.
96. Robins K, Lyons G (2007) Opioid-related narcosis in a woman with myopathy receiving magnesium. Int J Obstet Anesth 16: 367-369.

97. Moriarty KT, McFarland R, Whittaker R, Burch J, Turnbull HE, et al. (2008) Pre-eclampsia and magnesium toxicity with therapeutic plasma level in a woman with m.3243A>G melas mutation. J Obstet Gynaecol 28: 349.
98. Archer TL, Heitmeyer JD (2010) Perioperative hemodynamics obtained by pulse contour analysis facilitated the management of a patient with chronic hypertension, renal insufficiency, and superimposed preeclampsia during cesarean delivery. J Clin Anesth 22: 274-279.
99. Chan SM, Lu CC, Ho ST, Liaw WJ, Cherng CH, et al. (2008) Eclampsia following cesarean section with HELLP syndrome and multiple organ failure. Acta Anaesthesiol Taiwan 46: 46-48.
100. Nethravathi M, Panicker JN, Taly AB, Arunodaya GR, Sinha S (2007) Therapeutic magnesium for eclampsia: an unusual cause for antepartum flaccid paralysis. J Postgrad Med 53: 79-80.
101. Saitoh K, Motegi R, Hirabayashi Y, Shimizu R (1994) [Cardiac arrests probably induced by hypermagnesemia during anesthesia for caesarean section]. Masui 43: 388-391.
102. Baraka A, Dajani A, Jabagi S (1984) Magnesium-induced respiratory arrest in a parturient recovering from general anesthesia-a case report. Middle East J Anesthesiol 7: 437-440.
103. Nguyen TTT, Koenders MEF, Wierda JMKH (2001) [Drug interaction in a pregnancy complicated by pre-eclampsia and cesarean section]. Nederlands Tijdschrift voor Anesthesiologie 14 (1): 21-25.
104. Fay TN, Bogod D (1996) Magnesium sulphate: the time for reckoning. Br J Obstet Gynaecol 103: 852.
105. Funai Y, Ikeda Y, Takahashi R, Asada A (2010) [Prolonged apnea from rocuronium in a patient with hypermagnesemia after cesarean section: a case report]. Masui 59: 721-723.
106. Hino H, Kaneko I, Miyazawa A, Aoki T, Ishizuka B, et al. (1997) [Prolonged neuromuscular blockade with vecuronium in patient with triple pregnancy treated with magnesium sulfate]. Masui 46: 266-270.
107. Kwan WF, Lee C, Chen BJ (1996) A noninvasive method in the differential diagnosis of vecuronium-induced and magnesium-induced protracted neuromuscular block in a severely preeclamptic patient. J Clin Anesth 8: 392-397.
108. Sinatra RS, Philip BK, Naulty JS, Ostheimer GW (1985) Prolonged neuromuscular blockade with vecuronium in a patient treated with magnesium sulfate. Anesth Analg 64: 1220-1222.
109. Yoshida A, Itoh Y, Nagaya K, Takino K, Sugawara J-I, et al. (2006) Prolonged relaxant effects of vecuronium in patients with deliberate hypermagnesemia: time for caution in cesarean section. J Anesth 20: 33-35.
110. Sloan PA, Rasul M (2002) Prolongation of rapacuronium neuromuscular blockade by clindamycin and magnesium. Anesth Analg 94: 123-124.
111. Ben-Ami M, Giladi Y, Shalev E (1994) The combination of magnesium sulphate and nifedipine: a cause of neuromuscular blockade. Br J Obstet Gynaecol 101: 262-263.
112. Snyder SW, Cardwell MS (1989) Neuromuscular blockade with magnesium sulfate and nifedipine. Am J Obstet Gynecol 161: 35-36.
113. Wu Q-A, Ye Y-Q (2010) Neuromuscular blockade after therapy with magnesium sulfate and amlodipine. Eur J Obstet Gynecol Reprod Biol 149: 225.
114. Pittman JA (2000) Magnesium sulphate for pre-eclampsia and a sudden bradycardia. Br J Anaesth 85: 327-328.
115. Scardo JA, Brost BC, Sola E, Newman RB, Tate SB (1997) Severe hypotension with nifedipine-magnesium in severe preeclampsia: A hemodynamic observation. J Matern Fetal Investig 7: 152-154.
116. Waisman GD, Mayorga LM, Camera MI, Vignolo CA, Martinotti A (1988) Magnesium plus nifedipine: potentiation of hypotensive effect in preeclampsia? Am J Obstet Gynecol 159: 308-309.
117. Awwad JT, Khalil AM, Aswad NK, Suidan FJ, Karam KS (1994) Labial edema in pregnancy. A case report. J Reprod Med 39: 921-922.
118. Basaran A, Bozdag G, Aksu AT, Deren O (2007) Twin pregnancy complicated with acute appendicitis and cholecystitis in the same gestational period. Arch Gynecol Obstet 276: 291-293.
119. Haldeman W (1993) Can magnesium sulfate therapy impact lactogenesis? J Hum Lact 9: 249-252.

120. Hill WC, Gill PJ, Katz M (1985) Maternal paralytic ileus as a complication of magnesium sulfate tocolysis. Am J Perinatol 2: 47-48.
121. Hung J-W, Tsai M-Y, Yang B-Y, Chen J-F (2005) Maternal osteoporosis after prolonged magnesium sulfate tocolysis therapy: a case report. Arch Phys Med Rehabil 86: 146-149.
122. Lurie S, Rotmensch S, Feldman N, Glezerman M (2002) Breast engorgement and galactorrhea during magnesium sulfate treatment of preterm labor. Am J Perinatol 19: 239-240.
123. Riggs JE, Hogg JP (2000) Central pontine myelinolysis: association with parenteral magnesium administration. Mil Med 165: 494-495.
124. Sameshima H, Higo T, Kodama Y, Ikenoue T (1997) Magnesium tocolysis as the cause of urinary calculus during pregnancy. J Matern Fetal Med 6: 296-297.
125. Spital A, Greenwell R (1991) Severe hyperkalemia during magnesium sulfate therapy in two pregnant drug abusers. South Med J 84: 919-921.
126. Roberts D, Haslett E, Hickey-Dwyer M, McCormack J (1998) Eclampsia complicated by bilateral retinal detachments and abnormal eye movements. Lancet 351: 803-804.
127. Thorp JM, Jr., Katz VL, Campbell D, Cefalo RC (1989) Hypersensitivity to magnesium sulfate. Am J Obstet Gynecol 161: 889-890.
128. Bourgeois FJ, Thiagarajah S, Harbert GM, Jr., DiFazio C (1986) Profound hypotension complicating magnesium therapy. Am J Obstet Gynecol 154: 919-920.
129. Rodis JF, Vintzileos AM, Campbell WA, Deaton JL, Nochimson DJ (1987) Maternal hypothermia: an unusual complication of magnesium sulfate therapy. Am J Obstet Gynecol 156: 435-436.
130. Cardosi RJ, Chez RA (1998) Magnesium sulfate, maternal hypothermia, and fetal bradycardia with loss of heart rate variability. Obstet Gynecol 92: 691-693.
131. Hennessy A, Hill I (1999) A case of maternal bradycardia at therapeutic doses of magnesium sulphate in preeclampsia. Aust N Z J Obstet Gynaecol 39: 256-257.
132. Oettinger M, Perlitz Y (1993) Asymptomatic paroxysmal atrial fibrillation during intravenous magnesium sulfate treatment in preeclampsia. Gynecol Obstet Invest 36: 244-246.
133. Pritchard JA (1979) The use of magnesium sulfate in preeclampsia-eclampsia. J Reprod Med 23 (3): 107-114.
134. Herschel M, Mittendorf R (2001) Tocolytic magnesium sulfate toxicity and unexpected neonatal death. J Perinatol 21: 261-262.
135. Tang F, Xiao B, Xiong Q, Yang M (2010) A case report: magnesium intoxication occurring in the process of total serum magnesium decrease. J Obstet Gynaecol Res 36: 174-177.
136. Sherer D, Cialone P, Abramowicz J, Woods J (1992) Transient symptomatic subendocardial ischemia suring magnesium sulfate tocolytic therapy. Am J Obstet Gynecol 166: 33-35.
137. Koontz SL, Friedman SA, Schwartz ML (2004) Symptomatic hypocalcemia after tocolytic therapy with magnesium sulfate and nifedipine. Am J Obstet Gynecol 190: 1773-1776.
138. Mayan H, Hourvitz A, Schiff E, Farfel Z (1999) Symptomatic hypocalcaemia in hypermagnesaemia-induced hypoparathyroidism, during magnesium tocolytic therapy - Possible involvement of the calcium-sensing receptor. Nephrol Dial Transplant 14 (7): 1764-1766.
139. Monif GR, Savory J (1972) Iatrogenic maternal hypocalcemia following magnesium sulfate therapy. JAMA 219: 1469-1470.
140. Nassar AH, Salti I, Makarem NN, Usta IM (2007) Marked hypocalcemia after tocolytic magnesium sulphate therapy. Am J Perinatol 24: 481-482.
141. Ganzevoort JW, Hoogerwaard EM, van der Post JAM (2002) [Hypocalcemic delirium due to magnesium sulphate therapy in a pregnant woman with pre-eclampsia]. Ned Tijdschr Geneeskd 146: 1453-1456.
142. Elliott JP, O'Keeffe DF, Greenberg P, Freeman RK (1979) Pulmonary edema associated with magnesium sulfate and betamethasone administration. Am J Obstet Gynecol 134: 717-719.
143. Worrell JA, Brunner JP, O'Donnell DM, Carroll FE (1992) Pulmonary edema associated with tocolytic therapy. AJR Am J Roentgenol 158 (6): 1356-1357.
