# Supplementary material for: Maternal adverse effects of different antenatal magnesium sulphate regimens for improving maternal and infant outcomes: a systematic review
Source: BMC Pregnancy Childbirth. 2013 Oct 21;13:195. doi: 10.1186/1471-2393-13-195 (PMC4015216; doi:10.1186/1471-2393-13-195)
Supplement: Additional file 4 — Characteristics of included studies. [file 1471-2393-13-195-S4.pdf]

Characteristics of included studies.

Reference	Participants	Interventions (magnesium sulphate regimen(s) and control if applicable)	Adverse effects~	Quality assessment/risk of bias
**Randomised controlled trials**				
**Magnesium sulphate versus placebo or no treatment**				
Chen 1995 [1]<br>TAIWAN, 1 hospital<br>1989-1992	64 women. Inclusions: BP ≥ 150/100 mmHg, plus ≥ one of 11 listed features of severe PE. Exclusions: intrauterine death, chronic HTN or eclampsia.	Intervention group (n=34): 4 g IV LD over 10 mins; 1 g/hour MD until 24 hours after delivery. Control group (n=30): no anticonvulsant.	Generalised weakness; caesarean	Sequence generation and allocation concealment: unclear risk of bias (not detailed). Blinding: high risk of bias (not described - unlikely in view of intervention). Incomplete outcome data/losses: low risk of bias (no losses to follow-up). Selective reporting: unclear risk of bias (outcomes not clearly pre-specified; incomplete AE reporting - detailed in discussion).
Coetzee 1998 [2]<br>SOUTH AFRICA, 1 hospital<br>1982-1995	822 women. Inclusions: severe PE, decision to terminate pregnancy. Exclusions: < 16 years; received MgSO4 /other anticonvulsant.	Intervention group (n=345): 4 g IV LD over 20 mins; 1 g/hour MD until 24 hours after delivery. Control group (n=340): placebo solution by identical regimen.	Death; given calcium gluconate; absent tendon reflexes; respiratory depression; caesarean	Sequence generation: unclear risk of bias (not detailed). Allocation concealment: high risk of bias (by sealed opaque envelopes (cards marked A or B), cards not envelopes numbered consecutively; envelopes distributed in batches of 20, with equal A and B). Blinding: low risk of bias (personnel and women blinded). Incomplete outcome data/losses: unclear risk of bias (123 envelopes/data sheets could not be found; 14 additional women excluded; 83% of women included in analyses). Selective reporting: unclear risk of bias (outcomes not clearly pre-specified).
Cox 1990 [3]<br>USA, 1 hospital<br>1987-1989	156 women. Inclusions: PTL; 24-34 weeks GA; intact fetal membranes. Exclusions: ROM or maternal/fetal complications requiring delivery.	Intervention group (n=76): 4 g IV LD; 2 g/hour MD, increased to 3 g/hour if uterine contractions persisted after 1 hour; for 24 hours. Control group (n=80): saline solution (80 ml/hour for 24 hours).	Cessation of therapy due AE; respiratory depression; systemic hypotension; generalised muscle weakness; caesarean	Sequence generation: unclear (random number table). Allocation concealment: low risk of bias (consecutive numbered, sealed, envelopes). Blinding: high risk of bias (not detailed; Mg concentration measurement for intervention group suggests no blinding). Incomplete outcome data/losses: low risk of bias (outcomes reported for all neonates/mothers). Selective reporting: unclear risk of bias (outcomes not clearly pre-specified).
Crowther 2003 [4]<br>AUSTRALIA AND NEW ZEALAND, 16 hospitals<br>1996-2000	1062 women. Inclusions: < 30 weeks GA, singleton or higher order pregnancy, delivery expected < 24 hours. Exclusions: 2nd stage of labour, received MgSO4 in this pregnancy, contraindications to MgSO4.	Intervention group (n=535): 4 g IV LD over 20 mins; 1 g/hour MD until birth or for 24 hours, which ever occurred first. Control group (n=527): equal volume of placebo solution (isotonic sodium chloride solution 9%).	Death; cardiac arrest; respiratory arrest; cessation of infusion due to AE; respiratory depression; hypotension; tachycardia; any AE; warmth over body; nausea; sleepiness; mouth dryness; dizziness; sweating; blurred vision; arm discomfort; caesarean; PPH; ICU admission	Sequence generation: low risk of bias (central computer-generated randomisation sequence). Allocation concealment: low risk of bias (central telephone randomisation). Blinding: low risk of bias (women, caregivers and outcome assessors blinded). Incomplete outcome data/losses: low risk of bias (hospitalisation outcomes given for all women/infants). Selective reporting: low risk of bias (outcome pre-specified; no indication of selective reporting).
Duley 2002 [5]<br>33 countries (Africa, the Americas, Asia-Pacific, Europe), 175 hospitals<br>1998-2001	10141 women. Inclusions: uncertainty about whether to use MgSO4, before birth or 24 hours postpartum, DBP ≥ 90 mmHg, SBP ≥ 140 mmHg on more than 2 occasions, ≥ 1+ proteinuria, singleton or multiple pregnancy. Exclusions: hypersensitivity to Mg, hepatic coma with risk of renal failure, MG.	Intervention group (n=5055): 4 g IV LD over 10-15 mins; then 1 g/hour IV or 10 g IM (5 g each buttock) with the initial LD, followed by 5 g IM MD every 4 hours; for 24 hours. Control group (n=5055): a placebo by identical regimen.	Death; cardiac arrest; respiratory arrest; cessation of therapy due to AE; given calcium gluconate; respiratory depression; absent/reduced reflexes; hypotension, palpitations or tachycardia; any AE; flushing; nausea and or vomiting; muscle weakness; drowsiness/confusion; headache; thirst; dizziness;	Sequence generation: low risk of bias (central computer-generated randomisation sequence). Allocation concealment: low risk of bias (central pharmacy randomisation or consecutively numbered, sealed treatment packs). Blinding: low risk of bias (women, caregivers and outcome assessors blinded). Incomplete outcome data/losses: low risk of bias (5 women were excluded (2 in each group - no data; 1 in intervention group - wrong trial); follow up data available for 99.7% of women randomised before delivery and 98.6% of babies). Selective reporting: low risk of bias (outcomes pre-specified; no indication of selective reporting).

			itchy/tingling; problems at IV/IM site; pulmonary oedema; caesarean; blood loss after delivery > 500 ml; ICU admission	
Livingston 2003 [6]<br>USA, 1 hospital<br>1995-2001	222 women. Inclusions: mild PE during labour. Exclusion: chronic hypertension, severe PE.	Intervention group (n=109): 6 g IV LD over 20 mins; 2 g/hour MD, until 12 hours postpartum or a total of 12 hours for those randomised after delivery. Control group (n=113): identical saline placebo.	Toxicity (absent tendon reflexes/respiratory depression); pulmonary oedema; caesarean	Sequence generation: low risk of bias (central computer-generated simple randomisation sequence). Allocation concealment: low risk of bias (consecutively numbered, sealed opaque envelopes). Blinding: low risk of bias (investigators, women and outcome assessors blinded; if women developed severe PE after randomisation, group assignment revealed). Incomplete outcome data/losses: low risk of bias (no losses reported). Selective reporting: unclear risk of bias (outcomes not clearly pre-specified).
Ma 1992 [7]<br>CHINA, 1 hospital<br>1989-1991	65 women. Inclusions: uncomplicated PTL, 28-36 weeks GA. Exclusions: complicated PTL.	Intervention group (n=30): 5 g IV LD; 2 g/hour MD - duration unclear. Control group (n=35): barbiturates or best rest.	Cessation of therapy due to AE	Sequence generation and allocation concealment: unclear risk of bias ('they were divided into two groups randomly'). Blinding: high risk of bias (no use of placebo; unlikely in view of intervention). Incomplete outcome data/losses: low risk of bias (no losses reported). Selective reporting: unclear risk of bias (unclear; outcomes not clearly pre-specified).
Marret 2007 [8]<br>FRANCE, 18 hospitals<br>1997-2003	573 women. Inclusions: < 33 weeks GA, singleton, twins, or triplets, delivery planned or expected < 24 hours. Exclusions: severe fetal congenital/chromosomal abnormalities, contraindications to MgSO4, indication for emergency caesarean, pregnancy associated vascular disease.	Intervention group (n=286): 4 g IV LD over 30 mins, no MD or repeat treatment. Control group (n=278): equal volume of placebo solution (isotonic saline 0.9%).	Death; cardiac arrest; respiratory arrest; tendon reflex abolition; hypotension; flushing; nausea or vomiting; headache; caesarean; PPH	Sequence generation: low risk of bias (central computer-generated randomisation sequence). Allocation concealment: low risk of bias (central telephone randomisation). Blinding: unclear (obstetricians and anaesthetists not blinded; infant/child outcome assessors blinded). Incomplete outcome data/losses: unclear risk of bias (573 women randomised - 564 included in analyses; outcomes obtained until discharge for all women/infants). Selective reporting: low risk of bias (outcomes pre-specified; no indication of selective reporting).
Moodley 1994 [9]<br>SOUTH AFRICA, 1 hospital	228 women. Inclusions: severe PE, or imminent eclampsia, requiring delivery. Exclusions: prior anticonvulsant or antihypertensive.	Intervention group (n=112): (Pritchard's regimen) 4 g IV LD over 20 mins and 10 g IM (5 g each buttock); then 5 g MD 4 hourly for 24 hours (max of 6 doses). Control group (n=116): no anticonvulsant.	Pulmonary oedema; caesarean	Sequence generation: unclear risk of bias ('randomly distributed'). Allocation concealment: low risk of bias (consecutively numbered, sealed, opaque envelopes). Blinding: high risk of bias (designed as an open trial). Incomplete outcome data/losses: low risk of bias (data complete for all outcomes). Selective reporting: unclear risk of bias (outcomes not clearly pre-specified; incomplete AE reporting).
Rouse 2008 [10]<br>USA, 20 hospitals<br>1997-2004.	2241 women. Inclusions: 24-31 weeks GA, singleton or twins, high risk for spontaneous delivery, PPROM, PTL or indicated delivery within 2-24 hours. Exclusions: delivery anticipated < 2 hours, cervical dilatation > 8 cm, PPROM < 22 weeks, major fetal anomalies, IUFD, HTN/PE, contraindications to MgSO4 or received in prior 12 hours.	Intervention group (n=1096): 6 g IV LD over 20-30 mins; 2 g/hour MD stopped if delivery had not occurred in 12 hours. For repeat treatment: if < than 6 hours had transpired since cessation, MD resumed, if ≥ 6 hours had transpired, additional LD before MD. Control group (n=1145): identical appearing placebo.	Death; cardiac arrest; respiratory arrest; cessation of therapy due to AE; respiratory depression; any AE; flushing; nausea or vomiting; sweating; pain/burning at IV site; caesarean; pulmonary oedema	Sequence generation: low risk of bias (central computer-generated randomisation sequence). Allocation concealment: low risk of bias (central pharmacy randomisation). Blinding: low risk of bias (women, caregivers and outcome assessors blinded). Incomplete outcome data/losses: low risk of bias (follow up of surviving infants 95%; AE reported only for mothers who received study medications (98% each group)). Selective reporting: low risk of bias (outcomes pre-specified; no indication of selective reporting).
Witlin 1996 [11]<br>USA, 1 hospital<br>1995-1996.	135 women. Inclusions: ≥ 37 weeks GA with recent onset PE. Exclusions: severe PE, fetal malpresentation, congenital anomalies, non-reassuring fetal testing, contraindications to MgSO4 or to a trial of labour.	Intervention group (n=67): 6 g IV LD over 15-20 mins; 2 g/hour MD infusion continued until 12 hours postpartum. Control group (n=68): saline placebo solution by an identical regimen.	Cessation of therapy due to AE; feeling warm and flushed; lethargy; slurred speech; caesarean; PPH	Sequence generation: low risk of bias (computer generated table of random numbers). Allocation concealment: low risk of bias (sequentially numbered, sealed, opaque envelopes). Blinding: low risk of bias (women and caregivers blinded). Incomplete outcome data/losses: low risk of bias (no losses reported). Selective reporting: low risk of bias (outcomes pre-specified; no indication of selective reporting).

**Magnesium sulphate versus different magnesium sulphate**				
**Lower dose versus higher dose magnesium sulphate IM maintenance**				
Malapaka 2011 [12]<br>INDIA, 1 hospital<br>2007-2009	126 women. Inclusions: eclampsia/imminent eclampsia.	Intervention group (n=72): 4 g IV LD over 15-20 mins; 2 g MD every 3 hours by IM or slow IV. 2 g IM/slow IV for recurrent convulsions. Control group (n=54): (Pritchard's regimen) 4 g IV LD slowly, with 10 g IM; 5 g IM every 4 hours in alternate buttocks. Both groups: for 24 hours after last convulsion or delivery, whichever was later.	Death due to toxicity; stopped dosage due to toxicity; skipped doses due to toxicity; respiratory depression; absent knee jerk; gluteal abscess; pulmonary oedema; PPH; acute respiratory failure	Sequence generation and allocation concealment: unclear risk of bias (not detailed). Blinding: high risk of bias (not detailed - unlikely for personnel and women in view of the intervention; unclear for outcome assessors). Incomplete outcome data/losses: low risk of bias (outcomes complete). Selective reporting: unclear risk of bias (outcomes not clearly pre-specified). Unbalanced randomisation *Intervention group: 37 (51%) women with imminent eclampsia, 35 (49%) with eclampsia. Control group: 16 (30%) women with imminent eclampsia, 38 (70%) women with eclampsia.
Shilva 2007 [13]<br>INDIA, 1 hospital	50 women. Inclusions: antepartum eclampsia. Exclusions: renal failure, pulmonary oedema, received MgSO4 before coming to the hospital.	Intervention group (n=25): (Low-dose Dhaka regimen) 4 g IV and 6 g IM LD; 2.5 g IM every 4 hours for 24 hours. Control group (n=25): (High-dose regimen) 4 g IV and 8 g IM LD; 4 g IM every 4 hours for 24 hours. Additional 2 g given IV for recurrent seizures.	Requirement for calcium gluconate; deferred doses due to AE; absent knee jerk reflex	Sequence generation: low risk of bias (patients randomised using a 'Tippet table'). Allocation concealment: unclear risk of bias (not detailed). Blinding: high risk of bias (not detailed - unlikely for personnel and women in view of the intervention; unclear for outcome assessors). Incomplete outcome data/losses: low risk of bias (no losses to follow up detailed). Selective reporting: unclear risk of bias (outcomes not clearly pre-specified).
**Lower dose versus higher dose magnesium sulphate IV maintenance**				
Behrad 2003 [14]<br>IRAN, 1 hospital<br>2000-2001	100 women. Inclusions: singleton or twin pregnancy, 24-35 weeks GA, spontaneous PTL. Exclusions: higher-order multiple pregnancy, ROM, non-reassuring fetal assessment, intrauterine infection, vaginal bleeding, history of DM, MG/other neuromuscular disease, impaired renal function, maternal bradycardia, atrioventricular block.	Intervention group (n=50): 6 g IV LD; 2 g/hour MD - increased by 1 g/hour hourly until successful tocolysis or failure (max 4 g/hour). Control group (n=50): 4 g IV over 20 mins; 2 g/hour MD.	Cessation of therapy due to AE (severe hypotension, pulmonary oedema, respiratory depression); no AE; flushing; nausea/vomiting; headache; caesarean	Sequence generation: low risk of bias (computer generated random number allocation). Allocation concealment: unclear risk of bias (consecutively numbers opaque envelopes - unclear if sealed). Blinding: high risk of bias (not detailed - unlikely for personnel and women in view of the intervention; unclear for outcome assessors). Incomplete outcome data/losses: low risk of bias (outcomes assessed for all women during hospitalisation). Selective reporting: unclear risk of bias (not all outcomes reported were pre-specified).
Terrone 2000 [15]<br>USA, 1 hospital<br>1997-1998	160 women. Inclusions: singleton or twin pregnancy, between 24-34 weeks GA, spontaneous PTL. Exclusions: higher-order multiple pregnancy, ROM, non-reassuring fetal assessment, intrauterine infection, treatment with any tocolytic agent before transport, unable to tolerate high dose MgSO4.	Intervention group (n=82): 4 g IV LD over 20 mins; 5 g/hour MD. Control group (n=78): 4 g IV LD over 20 mins; 2 g/hour MD. If after 1 hour the patient continued to have contractions, further cervical dilatation or effacement, MD was increased by 1 g/hour (max 6 g/hour).	Cessation of therapy due to AE; no AE; flushing; headache; pulmonary oedema; caesarean	Sequence generation: low risk of bias (computer generated random number allocation). Allocation concealment: unclear risk of bias (consecutively numbered, opaque envelopes - not detailed if sealed). Blinding: high risk of bias (not detailed - unlikely for personnel and women in view of the intervention; unclear for outcome assessors). Incomplete outcome data/losses: unclear risk of bias (12 women who were delivered due to failed tocolysis were excluded (8 intervention (10.3%); 4 control group (4.9%)); were more likely to develop pulmonary oedema). Selective reporting: unclear risk of bias (outcomes not pre-specified).
**Magnesium sulphate IV maintenance versus IM maintenance**				
Bhattacharjee [16]<br>2011<br>INDIA, 2 hospitals<br>2007-2009	144 women. Inclusions: eclampsia. Exclusions: eclampsia with added complication(s) and referred cases who had already received initial dose of MgSO4.	Intervention group (n=72): 4 g IV slow bolus LD; 0.75 g/hour IV MD. Control group (n=72): (Pritchard's regimen) 4 g IV LD slowly and 10 g IM (5 g in each buttock); 5 g IM every 4 hours in alternate buttocks. Additional 2 g IV given for convulsions.	Death; MgSO4 toxicity; Caesarean; PPH	Sequence generation: low risk of bias (computer generated randomisation). Allocation concealment: low risk of bias (sealed, sequentially, numbered, brown envelopes). Blinding: high risk of bias (women/caregivers not blinded due to the nature of the intervention). Incomplete outcome data/losses: unclear risk of bias (5/72 women in intervention, and 2/72 women in control group were excluded from analysis due to defective record-keeping). Selective reporting: low risk of bias (outcomes pre-specified; no evidence of

				selective reporting).
Chissell 1994 [17] SOUTH AFRICA, 1 hospital	17 women. Inclusions: severe PE. Patients with hypertension and proteinuria with additional symptoms/signs - headache, visual disturbances, increased patellar reflexes that were considered to have impending eclampsia.	Intervention group (n=8): 6 g IV LD over 15 mins; 2 g/hour IV MD for 24 hours or until delivery. Control group (n=9): 4 g IV LD over 15 mins, and 5 g IM each buttock; 5 g in alternate buttocks every 4 hours for 24 hours or until delivery.	Cessation of therapy due to AE; clinical signs of toxicity (oliguria, decreased patellar reflexes); caesarean	Sequence generation: unclear risk of bias (not detailed). Allocation concealment: unclear risk of bias (not detailed). Blinding: high risk of bias (not detailed - unlikely for personnel and women in view of the intervention; unclear for outcome assessors). Incomplete outcome data/losses: low risk of bias (outcomes complete (3 intervention and 2 control group missing 19 hour serum albumin and calcium)). Selective reporting: unclear risk of bias (outcomes not clearly pre-specified).
Mundle 2009 [18] INDIA, 2 hospitals	200 women. Inclusions: BP ≥ 140/100 mmHg and proteinuria ≥1+ 930 mg/dl, and determined by clinic care team to benefit from MgSO4.	Intervention group (n=150): MgSO4 for 24 hours by Springfusor pump. Control group (n=150): MgSO4 for 24 hours by standard hospital practice (manual IV LD; IM maintenance)	Discontinuation or modification of treatment (need assessed by clinicians); pain level acceptable	Sequence generation and allocation concealment: unclear risk of bias (not detailed). Blinding: high risk of bias (not detailed - unlikely for personnel and women in view of the intervention; unclear for outcome assessors). Incomplete outcome data/losses: low risk of bias (no losses reported). Selective reporting: low risk of bias (outcomes pre-specified; no evidence of selective reporting). Published as abstract.
**Ready to use magnesium sulphate solution versus reference drug (requiring dilution)**				
Zygmunt 2003 [19] GERMANY, 3 hospitals	46 women. Inclusions: PTL, 25-36 weeks GA, single agent tocolysis indicated for ≥ 48 hours. Exclusions: dilatation of cervix > 2 cm, impaired renal function, total bilirubin > 2 mg/dl, 1st-3rd degree AV-block/other cardiac conduction disorders, MG, use of barbiturates, narcotics, hypnotics, aminoglycoside antibiotics, participation in a clinical study < 1 month prior.	Intervention group (n=23): RTU solution (24 g MgSO4 x $7H_2O$; 97.4 mmol Mg per 500 ml). Control group (n=23): reference drug (commercially available infusion solution concentrate needing dilution - 20 g MgSO4 diluted in 500 ml solution). Both groups: 4 g IV LD over 30 mins; 1-2 g/hour MD (depending on effect) for 21 days max.	Death; 'serious' AE; injection site changes; respiratory depression; poor general tolerability; local tolerability; AE of severe intensity; withdrawn from study due to AE; warmth; skin redness; burning at injection site; nausea/vomiting; headache; sweating; dizziness; dry mouth; palpitation; constipation; dyspnoea; heart pain; tiredness; agitation	Sequence generation: unclear risk of bias ('patients randomised based on randomly permuted blocks'). Allocation concealment: unclear risk of bias (investigators given set of sealed envelopes identified by patient's number containing the allocation of the individual patient number to one of two groups). Blinding: high risk of bias (not detailed - unlikely for personnel and women in view of the intervention; unclear for outcome assessors). Incomplete outcome data/losses: low risk of bias (patient questionnaire assessment (20/23 RTU) and (21/23) 89% response). Selective reporting: low risk of bias (outcomes pre-specified - assessment of 'general tolerability' by investigators unclear). Use of patient questionnaire may have influenced the high frequencies of AE.
**Short versus standard (24 hour) postpartum maintenance therapy**				
Ehrenberg 2006 [20] USA 2001-2004	200 women. Inclusions: mild PE, diagnosed antepartum, intrapartum or within 2 hours postpartum, delivering at ≥ 34 weeks GA. Exclusions: severe PE at delivery or before randomisation.	Intervention group (n=101): 2 g/hour for 12 hours after delivery. Control group (n=99): 2 g/hour for 24 hours after delivery. All women received a 4 g IV LD followed by 2 g/hour maintenance prior to delivery.	MgSO4 toxicity; intolerance	Sequence generation: low risk of bias (computer-generated random number table in blocks of 10). Allocation concealment: low risk of bias (consecutively numbered, sealed, opaque envelopes). Blinding: high risk of bias (not detailed - unlikely for personnel and women in view of the intervention; unclear for outcome assessors). Incomplete outcome data/losses: low risk of bias (4 women (2%) lost from the control group). Selective reporting: unclear risk of bias (outcomes not clearly pre-specified; AE not clearly reported).
Suneja 2008 [21] INDIA, 1 hospital	60 women. Inclusions: severe PE.	Both groups received MgSO4 from induction to delivery. In postpartum period: Intervention group (n=30): therapy until the clinical criteria were met (absence of persistent headache, visual symptoms, and epigastric pain; onset on spontaneous diuresis > 100 cc/hour for 2 hours; 50% of hourly BP < 150/100 with no reading > 160/100 in preceding 2 hours). Control group (n=30): 24 hours of MgSO4.	MgSO4 toxicity; AE	Sequence generation and allocation concealment: unclear risk of bias (not detailed). Blinding: high risk of bias (not detailed - unlikely for personnel and women in view of the intervention; unclear for outcome assessors). Incomplete outcome data/losses: unclear risk of bias (unclear in regards to missing data). Selective outcome reporting: high risk of bias (outcomes not fully reported). Meeting abstract.

Reference	Participants	Magnesium sulphate regimen(s)	Adverse effects	Quality assessment/risk of bias
**Non-randomised comparative studies with concurrent controls**				
Chowdhury [22] 2009<br>INDIA<br>2001-2005<br>NRT	630 women. Inclusions: antepartum/postpartum eclampsia, singleton/multiple pregnancies. Exclusions: convulsions due to epilepsy/other causes.	Group 1 (n=150): (low dose IV) 4 g IV LD over 2-3 mins; 0.6 g/hour MD. Group 2 (n=480): (Pritchard's regimen) 4 g IV LD over 2-3 mins, and 5 g IM in each buttock; 5 g IM MD in alternate buttocks every 4 hours. Both groups: ≥ 24 hours after delivery or last fit.	Loss of the knee jerk and oliguria; withdrawal from subsequent doses due to AE; respiratory depression; pain at injection site; major AE; caesarean section	Sequence generation and allocation concealment: high risk of bias (not a randomised trial. Allocation was to 'units within the department'). Blinding: high risk of bias (not appropriate to blind for personnel/women; no detail for outcome assessors). Incomplete data/losses to follow up: low risk of bias (no participant loss/incomplete data). Selective reporting: low risk of bias (outcomes pre-specified; no evidence of selective reporting). Baseline characteristics comparable between groups.
Mahajan 2007 [23]<br>INDIA<br>2005-2007<br>NRT	95 women. Inclusions: eclampsia with witnessed seizures. Exclusions: underlying seizure disorder, women with established complications.	Padhar regimens. Group 1 (n=37): 2 g IV and 4 g IM LD; 4 g IM every 4 hours. Group 2 (n=58): 2 g IV and 8 g IM LD; 4 g IM every 4 hours. Both groups: for 24 hours after delivery or last convulsion which ever was later.	Respiratory depression (abnormal RR); absent knee jerks, and MD omitted	Sequence generation and allocation concealment: high risk of bias (not randomised Group 1: women who came directly to the hospital; Group 2: women who had received diazepam/Phenergan at a referring hospital). Blinding: high risk of bias (not blinded). Losses to follow up: low risk of bias (no losses or exclusions; outcome data complete). Selective reporting: low risk of bias (outcomes pre-specified; no evidence of selective reporting).
Shoaib 2009 [24]<br>PAKISTAN<br>2004-2006<br>NRT	100 women. Inclusions: women with severe PE and impending eclampsia. Exclusions: women with PIH and mild-moderate PE.	Group 1 (n=50): 4 g IV and 10 g IM LD. Group 2 (n=50): (Pritchard's regimen) 4 g IV over 20 mins and 10 g IM LD; 5 g IM every 4 hours for 24 hours.	Respiratory failure/distress; cardiac arrest; nausea and vomiting; feeling warm and flushed; dizziness; irritation at the injection site; caesarean; death	Sequence generation and allocation concealment: high risk of bias (sampling technique was 'non-random purposive'). Blinding: high risk of bias (no detail - unlikely in view of the intervention). Incomplete outcome data: low risk of bias (data complete). Selective reporting: low risk of bias (no evidence of selective reporting). No detail of control for potential confounders (women in Group 2 were older, later GA at onset of PE and delivery).
Young 1977 [25]<br>USA<br>1974-1975<br>NRT	144 women. Inclusions: eclampsia or PE; diagnosis based on criteria of the American Committee on Maternal Welfare.	Group 1 (n=97): (Intravenous push) 10 g IM LD; 2 g slow intravenous 'push' every 1-2 hours (mean: 30 g). Group 2 (n=47): 10 g IM LD; continuous MD 1 g/hour (mean: 46 g)	Death; heat and flushing; respiratory effects	Sequence generation and allocation concealment: high risk of bias (not randomised). Blinding: high risk of bias (not detailed and unlikely in view of the intervention). Losses to follow up: low risk of bias (no losses detailed). Selective reporting: unclear risk of bias (outcomes/AE not clearly reported). Groups reported to be 'clinically comparable' but this was unclear.
Ales 1987 [26]<br>USA<br>1981<br>RC	393 women with hypertension complicating pregnancy, with a focus on 178 women whose hypertension was first documented in labour. MgSO4 (n= 64) v no MgSO4 (n=114).	Not detailed	Caesarean; caesarean due to failure to progress	Selection: low risk of bias (representative cohort, selected from all women who delivered during Jan-December 1981; non-exposed women selected from this same cohort; exposure ascertained from medical records). Comparability: unclear risk of bias (study controls for potential confounders in logistic regression analysis. Authors acknowledge the degree of cervical dilatation may have played a role in the failure of labour - this could not be accurately assessed; biases/practices of individual physicians represent another confounder). Outcome: unclear risk of bias (outcomes assessed by reference to medical records, no detail of whether this was independent blind assessment).
Assaley 1998 [27]<br>USA<br>BAS	18 women with PE. MgSO4 (n=15) v no MgSO4 (n=3).	4 g IV LD; MD infusion of 1-2 g/hour, until 12-24 hours postpartum (81.3 g mean total, 52.5-145 g range).	Prolonged bleeding time	Selection: unclear risk of bias (unclear). Comparability: unclear risk of bias (no baseline comparison of groups, or any control for potential confounders for bleeding time). Outcome: low risk of bias (objective outcomes unlikely to be affected by lack of blinding).
Kynczl-Leisure 1996 [28]<br>USA<br>BAS	12 women with PE. MgSO4 (n=9) v no MgSO4 (n=3).	'standard convulsion prophylaxis treatment with magnesium sulfate' (15-40 hours duration)	Prolonged bleeding time	Selection: unclear risk of bias (unclear). Comparability: unclear risk of bias (no detail or control for potential confounding factors - treated women had lower bleeding time at baseline).Outcome: high risk of bias (whilst objective outcomes unlikely to be affected by lack of blinding, bleeding time measured at different times for 2 groups; 1 woman from exposed group was excluded as post-partum measurement was taken 5 hours after discontinuation - no

				change in bleeding time was noted for this patient).
Magee 2005 [29]<br>USA<br>1997-2001<br>RC	377 women who received IV MgSO4 who had a hypertensive disorder of pregnancy but no PTL (obtained from ICD-9 computerised database). Group 1 (n=162): received nifedipine as antihypertensive agent v Group 2 (n=32): received another antihypertensive v Group 3 (n=183): received no antihypertensive.	4 g IV bolus LD, followed by infusion of 2 g/hour	Neuromuscular weakness (absent DTR, weakness, respiratory depression, neuromuscular blockage, calcium gluconate given); maternal hypotension; nausea/vomiting; drowsiness/confusion; dizziness; flushing; thirst, respiratory problems; dyspnoea; pulmonary oedema; maternal tachycardia; infusion rate decreased due AE; infusion rate stopped early due to AE	Selection: low risk of bias (all women (cohort) drawn from same time period, and exposure ascertained from medical records). Comparability: high risk of bias (study does not control for potential confounding factors identified on baseline comparisons (neurological disorders, severe hypertension)). Outcome: low risk of bias (outcomes (AE) assessed/abstracted independently by 2 reviewers, with complete outcomes for all subjects (however limited by what was in case records)).
Nassar 2006 [30]<br>LEBANON<br>1995-2003<br>RC	155 pregnant women ≥ 25 weeks GA admitted for PTL and tocolysis with MgSO4. Group 1 (n=78): received MgSO4 for > 48 hours (median time 6 days) v Group 2 (n=77): received MgSO4 for < 48 hours. Exclusions: requiring combination tocolysis, with an underlying disease.	Policy: 4 g IV LD over 20 mins; 2 g/hour MD which could be incrementally increased to achieve uterine quiescence for up to 4 g/hour.	Visual disturbances; ileus; osteopenia; chest tightness; pulmonary oedema; vulvar oedema; hypocalcaemia; ≥ 1 AE; discontinuation of therapy due to AE	Selection: low risk of bias (women from both groups drawn from same population/period; exposure obtained from secure medical records). Comparability: unclear risk of bias (study does not control for baseline imbalances - women in Group 1 more likely to have multiple gestations, be of an earlier GA). Outcomes: unclear risk of bias (2 authors collected data on a standard sheet to ensure uniformity from maternity record - i.e. not independent blind assessment of outcomes; a bias in reporting/checking for maternal AE on prolonged treatment is also possible)
Palmer 2009 [31]<br>CANADA<br>2004-2007<br>HCS*	76 women who received MgSO4 for eclampsia prophylaxis. Cases (n=29): received the new protocol (July 2006-December 2007) v controls (n=47): received the old protocol (December 2004-June 2006)	New protocol: 20% Mg (with separate bags used for LD and MD). Old protocol: 2-8% Mg (with same bag used for LD and MD).	Phlebitis; signs/symptoms of toxicity; calcium gluconate used; errors associated with failure to reset pump after LD; errors associated with a change to the order	Selection: low risk of bias (consecutive women in study period included (list generated from BC Perinatal Database Registry of obstetric patients)). Comparability: high risk of bias (no control for group imbalances - baseline characteristics, and additional aspects of the treatment received). Outcome: unclear risk of bias (Unclear who assessed outcomes in charts and whether this could be blinded; inconsistent documentation in the charts noted).
Park 2006 [32]<br>KOREA<br>2002-2003<br>RC	231 women (including 55 women with PE) who delivered requiring labour induction. Inclusions: singleton, vertex presentation, intact amniotic membranes, ≥ 32 weeks GA, absence of active labour, initial cervical exam ≤ 2 cm dilatation and ≤ 50% effacement, no previous uterine surgery, no contraindication to vaginal delivery. MgSO4 for PE (n=29) v no MgSO4 (202).	4 g IV LD over 20 mins; continuous MD infusion of 2 g/hour through labour and for a minimum of 24 hours postpartum.	Failed induction of labour	Selection: low risk of bias (representative cohort, selected from all women who delivered during the period). Comparability: unclear risk of bias (whilst study controls for potential confounding factors in logistic regression analysis, factors were not detailed). Outcome: unclear risk of bias (outcomes assessed by reference to hospital records by one reviewer, no detail of whether it was possible for assessment to be blinded).
Poggi 2003 [33]<br>USA<br>1993-2002<br>RCC	66 women with triplet pregnancies. Cases (n=15): women who developed pulmonary oedema v controls (n=50): women who did not develop pulmonary oedema.	Regimens unclear (in discussion general protocol detailed: 4 g IV LD; 2 g/hour MD; could be increased if tocolysis not achieved).	Pulmonary oedema	Selection: low risk of bias (case definition clear; based on data from medical records; of 71 women eligible, 66 had complete records (93%); controls selected from same community). Comparability: high risk of bias (did not control for PTL/PE, dose, other fluids received). Exposure: low risk of bias (ascertained from medical records; same method for cases/controls).
Ramanathan 1988 [34]	16 women in labour. Exposed (n=10): with PE who received MgSO4 v Non-	4 g LD IV over 15 mins, followed by 1 g/hour MD.	Reduction in pulmonary function	Selection: unclear risk of bias (no detail of how groups were selected for the study). Comparability: high risk of bias (study details potential confounding

USA<br>BAS	exposed (n=6): no PE; did not receive MgSO4.			factors - i.e. smoking, methods of pain relief/oxytocin use, with no control for imbalances). Outcome: unclear risk of bias (no detail of blind outcome assessment, however less likely to affect such objective outcomes)
Ramanathan 1988 [35]<br>USA<br>BAS	32 pregnant women. Group 1. (n=16): with PE undergoing labour augmentation during MgSO4 therapy v Group 2. (n=6): with PE receiving MgSO4 postpartum v Group 3. (n=10): normotensive; no MgSO4; undergoing labour induction.	6 g IV LD, followed by 2 g/hour MD.	Depression of neuromuscular transmission	Selection: unclear risk of bias (no detail of how women were selected for the study). Comparability: unclear risk of bias (study does not describe or control for any potential confounding factors). Outcome: unclear risk of bias (no detail of blind outcome assessment, however less likely to affect such objective outcomes).
Seyb 1999 [36]<br>USA<br>1996-1997<br>RC	1561 term, nulliparous women labouring with a singleton fetus in the vertex presentation ≥ 37 weeks GA. Exclusions: women undergoing caesarean delivery without labour, multiple gestations, preterm delivery, malpresentation. Compared MgSO4 for PE (n=54) v no MgSO4 (n=1507).	Unclear	Caesarean	Selection: low risk of bias (selection of large cohort - consecutive women in time period; exposure(s) ascertained from data collected at time of admission and subsequent review of hospital charts). Comparability: unclear risk of bias ('control for significant confounding variables' in final regression model to determine caesarean risks). Outcome: unclear risk of bias (whilst the objective outcome (caesarean) was assessed through hospital records, it was unclear if this was done independently with verification or blinded).
**Case series**				
Aali 2007 [37]<br>IRAN<br>PCS	50 women with severe PE or eclampsia.	4 g IV LD; 2 g/hour MD until 24 hours after delivery or last seizure.	Depressed patellar reflexes; caesarean; failed induction of labour	Selection: unclear - likely eligible women from study period approached. Confounding: no discussion of potential confounders - i.e. weight/renal function of women with high serum Mg.
Adewole 2000 [38]<br>NIGERIA<br>1997-1998<br>PCS	21 women with eclampsia.	4 g IV LD over 10 mins; 1 g/hour IV MD for 24 hours after delivery or last fit, whichever was longer. If further convulsions - additional 2 g IV given.	Serious AE; infusion stopped due to AE; AE (nausea, vomiting, dizziness); respiratory depression	Selection: all eligible women from study period (i.e. consecutive women) were included. Regular monitoring for AE.
Ahmed 2004 [39]<br>PAKISTAN<br>1999-2000<br>PCS	31 women with eclampsia.	'MgSO4 was given to eclamptic patients according to protocol.'	Respiratory depression	Selection: likely that eligible women from study period (i.e. consecutive) were included. 'Side effects…were noted.' Abstract only was able to be obtained.
Begum 2001 [40]<br>BANGLADESH<br>1998<br>PCS	65 women with eclampsia.	4 g IV LD over 15 mins, with 3 g IM in each buttock; 2.5 g IM every 4 hours in alternate buttocks for 24 hours.	Absent knee jerks (regarded as a sign of potential toxicity); respiratory depression	Selection: 90 women during the study period were receiving MgSO4; 65 were recruited due to time limitations/availability of the principal investigator. Women monitored hourly for signs of toxicity.
Bilgin 1994 [41]<br>TURKEY<br>PCS	18 women with severe PE or eclampsia.	4 g IV LD; 2 g/hour MD for 48 hours after delivery.	'Restrictive type of respiratory depression'	Selection: unclear how women were selected (i.e. if consecutive); small series. Study designed in early postpartum period to exclude effects of large pregnant uterus on respiratory function.
Cotton 1984 [42]<br>USA<br>PCS	5 women satisfying ≥ 1 criteria for severe PIH.	4 g IV LD over 15 mins; 1.5 g/hour MD.	'nausea and flushing'	Selection: unclear how women were selected 'Five patients…were chosen for the study.' Very small study population. Incomplete reporting of AE.
Dasari 2010 [43]<br>INDIA<br>2002-2007<br>RCS	49 women who died due to hypertensive disorders of pregnancy.	Not detailed.	Mg toxicity; respiratory arrest; death	Selection: likely all eligible women from study period were included (consecutive deaths).
Digre 1990 [44]<br>USA<br>1987-1989	13 women in PTL requiring IV MgSO4 tocolysis.	Protocol: 4 g IV LD over 15-20 mins; 2 g/hour MD (unclear guidelines - dosage increased for some patients).	Blurred vision; diplopia, photophobia; visual signs; abnormal visual acuity;	Selection: small series (unclear if all eligible women from study period (i.e. consecutive) included). Confounding: excluded women with PE/eclampsia (known possibility for visual disturbance). Use of careful interviewing and

PCS			headache; nausea; generalised weakness; impaired concentration-confusion; loss of DTR; respiratory depression; cardiac arrhythmias.	specific questioning in regards to symptoms may have increased the incidence of positive responses.
Donovan 1980 [45]<br>USA<br>1975-1976<br>PCS	20 women with mild to moderate PE treated with MgSO4 in labour.	According to 1 of 2 protocols: 2 g rapid IV LD over 10-15 mins; 1-1.5 g/hour IV MD for variable time postpartum, or 4 g rapid IV LD; 1-1.5 g/hour MD.	'Toxicity'; areflexia	Selection: unclear if all eligible women (i.e. consecutive) in the study period were considered for selection; very small series. AE: maternal DTR, BP and urinary output monitored hourly.
Ekele 2005 [46]<br>NIGERIA<br>2002<br>PCS	19 women with eclampsia.	Modified Prichard's regimen: 4 g IV LD over 10 mins and 5 g each buttock; 5 g IM MD every 5 hours (alternate buttocks). An additional 2 g IV given in case of seizure.	Loss of DTR; respiratory depression; skipped MD; death	Selection: small series (likely that eligible women from study period (i.e. consecutive) were included). Women monitored for signs of toxicity before each MD.
Elliot 1983 [47]<br>USA<br>1978-1983<br>RCS	355 women with PTL if MgSO4 was ordered for tocolysis.	According to a protocol of Steer and Petrie: 4 g IV bolus followed by 2 g/hour (decision to increase/decrease rate based on patient's clinical response - up to 3 g/hour).	Any AE; cessation due to AE; pulmonary oedema; chest pain; chest tightness; nausea and or flushing; drowsiness; blurred vision; burning in eyes/headache	Selection: likely all eligible women from study period were included (consecutive women for whom MgSO4 was ordered for tocolysis). Confounding: separation of results of tocolysis based on gestation, membranes intact, cervical exam; discussion of risks for 2 pulmonary oedema cases (e.g. twin gestation, PIH). Unclear assessment of AE: 'were recorded.'
Fuentes 1995 [48]<br>USA<br>1993-1994<br>PCS	24 women requiring MgSO4 (15 for tocolysis before ECV or for PTL, 9 for PE prophylaxis).	6 g IV LD over 20 mins; 2 g/hour MD.	Prolonged bleeding time	Selection: eligible women from study period (i.e. consecutive) screened for participation. Confounding: women with confounding factors (history of platelet/clotting disorders; drugs known to prolong bleeding) were excluded. Bleeding times randomly performed by 2 investigators; blind to the other bleeding time.
Getaneh 2010 [49]<br>ETHIOPIA<br>2006<br>RCS	95 women who received MgSO4 during the study period (69 for severe PE, 21 for eclampsia, 5 for other reason).	Guidelines for severe PE/E: 4 g over 20 mins IV LD; 1 g/hour MD, for 24 hours post-delivery/from last fit. Mean duration treatment: 43.6 hours (range 1-103).	Early discontinuation/dose adjustment: due to oliguria/renal failure/CNS depression	Selection: a form was placed in labour ward to register the chart numbers of women who received MgSO4 - 95/103 charts were available (92.2%); a third hospital was excluded as charts of the 3 cases could not be retrieved. AE: unclear if other AE were assessed in charts or only those leading to cessation.
Ghia 2000 [50]<br>USA<br>1998<br>PCS	15 women in PTL.	4 g IV bolus, followed by MD (unclear dosing, duration).	Reduced attention and rapid information processing ability	Selection: of 20 eligible women, 5 did not complete the full study and were excluded. 'Some patients with high Mg levels declined to participate in the study because of their inability to concentrate and an overall feeling of discomfort.' Control test used equivalent forms (same question types/different content) to limit recall bias. Questionaries explained orally to avoid selection bias. All tests administered by same examiner to limit inter-examiner variability.
Girard 2005 [51]<br>FRANCE<br>2000-2002<br>RCS	57 women with severe PE who received MgSO4..	4.5 g IV LD over 20 mins; 1.5 g/hour MD.	Overdose and therapy cessation; minor AE	Selection: unclear - likely that all eligible women from study period (i.e. consecutive) were included. Confounding: 25 women were treated with an antihypertensive in conjunction; 32 were not. AE: unclear how the data was obtained/by who. Abstract only (full-text in French).
Guzin 2010 [52]<br>TURKEY<br>PCS	50 women with PE.	6 g IV LD over 20 mins; 2 g/hour MD until 12 hours postpartum.	Prolonged bleeding time	Selection: unclear if all eligible women during period were screened for participation. Confounding: women with potential confounding factors (history of bleeding disorders; drugs known to prolong bleeding; medical disorders) were excluded.
Hales 1995 [53]<br>USA	48 women with singleton pregnancies and PTL; 24 women with twin	4-6 g IV LD over 20 mins; 1-3 g/hour MD (dose tapered 0.5 g/hour after 24	Areflexia; hypotension; RR < 12/min; chest pain/dyspnoea	Selection: eligible cases (twin pregnancies) from study period (i.e. consecutive) included. For each twin pregnancy there was a matched pair of

1989-1992<br>PCS	pregnancies and PTL.	hours of inhibiting labour) (duration therapy twins: 4-68 hours; singletons: 4-77 hours).	and need for ECG	singletons (according to cervical dilation, frequency of uterine contractions, parity, maternal age, GA) chosen without knowledge of effects of therapy. A standard form was used to record symptoms, BP etc. at 15-30 min intervals.
Harding 1997 [54]<br>UK<br>1995-1996<br>RCS	22 women who received MgSO4 for severe PE.	4 g IV LD over 20 mins; 2 g/hour MD until 24 hours post-delivery.	Flushing; drowsiness; decreased RR; cessation of treatment due to AE	Selection: 5/35 sets of notes were not available during the study period. AE: process for data extraction not detailed - difficult to determine quality of AE data from case notes.
Herpolsheimer 1991 [55]<br>USA<br>1989<br>PCS	10 women with PE in labour at term.	6 g IV LD over 30 mins; 2 g/hour MD.	Significant decrease in pulmonary function tests	Selection: eligible women from study period (i.e. consecutive) included. Confounding: all women non-smoking, nondyspneic women, with no history of underlying pulmonary, neuromuscular disease; all measurements made between uterine contractions, with women in same semi-recumbent position.
Jirapinyo 1990 [56]<br>THAILAND<br>1987-1988<br>PCS	15 women in PTL.	4 g IV LD over 15-20 mins; 1.5 h/hour MD; increased by 0.5 g/hour every 30 mins until tocolysis achieved or a max of 3.5 g/hour (after success, rate reduced to 2 g/hour; continued for 12 hours).	'nausea, vomiting, headache, flushing and palpitations'	Selection: eligible women from study period (i.e. consecutive) included. AE only reported in discussion.
Kfuri 2008 [57]<br>USA<br>2003-2005<br>RCS	146 errors associated with MgSO4 in obstetric patients.	Not detailed.	Obstetric medication error	Selection: errors from MEDMARX data base (large adverse drug event database in the US; internet accessible, anonymous) - limitations associated with reporting errors (voluntary; possibility of underreporting). AE: unable to determine regimen/specific AE associated with errors.
Little 2001 [58]<br>USA<br>1996-2001<br>RCS	10 class 2 hospital errors in the inpatient obstetric unit.	'Excess dosing with intravenous magnesium sulfate.'	Class 2 hospital errors: need for additional treatment or hospitalisation, but causing only temporary harm	Selection: errors from obstetric unit - limitations associated with reporting errors (voluntary; possibility of underreporting). AE: cases manifesting a known side effects of a drug were only reported if incorrect dose was given.
Moghadas 2007 [59]<br>IRAN<br>2002-2003<br>PCS	40 pregnant women admitted for threatened PTL.	4 g IV LD over 20 mins; 2 g/hour MD	Prolonged bleeding time	Selection: likely that all eligible women from study period (i.e. consecutive) were screened for participation. Confounding: exclusion criteria sought to eliminate potential sources (i.e. history of medications, personal/familial history of platelet/clotting disorders).
Mojadidi 1969 [60]<br>USA<br>1965-1969<br>RCS	20 eclamptic women who received MgSO4.	'Usually' 2-5 g IV then 10 g in 1L 5% dextrose and water as IV drip; maintained for ≤ 10 hours; then given IM (25 g max over 6 hours).	Respiratory depression, toxicity	Selection: likely all eligible women from study period were included (consecutive cases). Confounding: women were sedated. AE: incomplete/unclear reporting and assessment.
Omu 2008 [61]<br>KUWAIT<br>2002-2004<br>PCS	450 women with severe PE or eclampsia.	4 g IV LD over 20 mins; 1 g/hour MD for ≥ 24 hours postpartum.	Toxicity manifested by reduced tendon reflexes; other AE (flushing, nausea, vomiting, blocked nostrils, headaches)	Selection: eligible women from study period (i.e. consecutive) included. No potential confounding factors discussed. AE: 'Magnesium sulphate therapy was aggressively monitored.'
Pritchard 1984 [62]<br>USA<br>1955-1983<br>RCS	245 women with eclampsia who received MgSO4.	Protocol: 4 g IV no faster than 1 g/hour, and 10 g IM; 5 g IM 4 hourly in alternate buttocks until 24 hours after delivery/last convulsion. Detail of 4 cases:<br>1: Nearly 20 g injected IV; 2: 20 g over ~ 40 mins; 3: Postpartum IM MgSO4; 4: Unclear.	Intoxication; respiratory arrest; death; loss of PR; respiratory depression, caesarean; failed induction of labour	Selection: likely all eligible women from study period were included (consecutive cases); large series. AE: reported only 'intoxication.'
Raman 1995 [63]	736 women with eclampsia and	20 ml 20% IV over 3 mins and 20 ml	Transient depression of knee	Selection: eligible women from study period (i.e. consecutive) included. No

INDIA<br>1987-1989<br>RCS	imminent eclampsia who received MgSO4.	50% IM; 10 ml 50% IM every 4 hours; until 24 hours after delivery or last convulsion. Additional 10 ml 20% IV if convulsion not controlled < 30 mins.	jerk; acute respiratory arrest; respiratory depression; delayed recovery from anaesthesia; calcium gluconate	potential confounding factors discussed. AE: case records reviewed by a single reviewer; difficult to determine the quality of the AE data reported in case notes.
Sass 2007[64]<br>BRAZIL<br>2007<br>PCS	138 women with PE.	4 g IV LD; 2 g/hour IV MD.	Reduced muscular reflexes; drowsiness or confusion; headache; respiratory depression	Selection: unclear - likely eligible women (i.e. consecutive) from study period were approached. AE: unclear how assessed/by who. Abstract only.
Thapa 2008 [65]<br>NEPAL<br>2006-2007<br>RCS	68 women with eclampsia and 9 with severe PE who received MgSO4.	'Pritchart's regime of magnesium sulphate.'	Cessation of therapy; toxicity	Selection: unclear - likely that all eligible women from study period (i.e. consecutive) were included. AE: unclear how the data was obtained/by who.
Tukur 2010 [66]<br>NIGERIA<br>2002-2007<br>RCS	93 women with eclampsia who received MgSO4.	Unclear - 'The Zuspan regimen (in which the maintenance doses are given intravenously) is used.'	Toxicity	Selection: likely all eligible women from study period were included (consecutive cases) - of 163 eclamptic patients during period, 131 case files were retrieved (80%), of these 93 received MgSO4. AE: incomplete/unclear reporting and assessment.
Yazdani 2004 [67]<br>IRAN<br>1999<br>PCS	30 women regarded as having PTL.	4 g IV LD over 20 mins, followed by 2 g/hour MD.	Prolonged bleeding time	Selection: women 'randomly selected' however no detail of methods of selection. Confounding: exclusion criteria sought to eliminate potential sources of confounding (i.e. history of medications, personal/familial history of bleeding disorders).
Yeast 1993 [68]<br>USA<br>1989-1990<br>PCS	294 women with PTL (193 courses of MgSO4 therapy) or PE (124 courses of MgSO4 therapy).	PE: 4 g IV bolus; 2 g/hour MD, for minimum 24 hours (usually 48 hours after delivery). Tocolysis: 6 g IV bolus; 3 g/hour MD, usually for 48 hours (often longer).	Symptomatic pulmonary oedema	Selection: likely that all eligible women from study period (i.e. consecutive) were included. Of 120 women with PE, 8 were excluded due to insufficient data and multiple courses of therapy - of 174 women with PTL, 6 were excluded. AE: somewhat unclear how outcome data were obtain/by who: 'COP values obtained for these patients served as the database.'

~Adverse effects, or other relevant outcomes, reported in the study
Abbreviations: AE: adverse effect(s); BAS: before and after study BP: blood pressure; DBP: diastolic blood pressure; DTR: deep tendon reflexes; g: gram(s); GA: gestational age; HCS: historical control study; HTN: hypertension; ICU: intensive care unit; IM: intramuscular; IV: intravenous; IUFD: intrauterine fetal death; LD: loading dose; MD: maintenance dose; MG: myasthenia gravis; MgSO4: magnesium sulphate Mg: magnesium; NRT: non-randomised trial; PCS: prospective case series; PE; pre-eclampsia; PIH: pregnancy-induced hypertension; PPH: postpartum haemorrhage; PPROM: preterm prelabour rupture of membranes; PTL: preterm/premature labour; RC: retrospective cohort study; RCC: retrospective case control study; RCS: retrospective case series; ROM: rupture of membranes; RR: respiratory rate; RTU: ready to use; SBP: systolic blood pressure
