# Supplementary material for: Maternal adverse effects of different antenatal magnesium sulphate regimens for improving maternal and infant outcomes: a systematic review
Source: BMC Pregnancy Childbirth. 2013 Oct 21;13:195. doi: 10.1186/1471-2393-13-195 (PMC4015216; doi:10.1186/1471-2393-13-195)
Supplement: Additional file 5 — Forest plots of Comparisons 1–5. [file 1471-2393-13-195-S5.pdf]

**Forest plots of Comparisons 1-5.** **Please note: Forest plots have been presented only for outcomes that were reported by more than one study*

| | Magnesium sulphate | | Placebo or no treatment | | | Risk Ratio |
|---|---|---|---|---|---|---|
| **Study or Subgroup** | **Events** | **Total** | **Events** | **Total** | **Weight** | **M-H, Fixed, 95% CI** |
| **1.1.1 Indication: pre-eclampsia or eclampsia** | | | | | | |
| Coetzee 1998 | 0 | 345 | 1 | 340 | 7.0% | 0.33 [0.01, 8.04] |
| Duley 2002 | 11 | 5055 | 20 | 5055 | 93.0% | 0.55 [0.26, 1.15] |
| **Subtotal (95% CI)** | | **5400** | | **5395** | **100.0%** | **0.53 [0.26, 1.09]** |
| Total events | 11 | | 21 | | | |
| Heterogeneity: Chi² = 0.09, df = 1 (P = 0.76); I² = 0% | | | | | | |
| Test for overall effect: Z = 1.72 (P = 0.09) | | | | | | |
| **1.1.2 Indication: fetal neuroprotection** | | | | | | |
| Crowther 2003 | 0 | 535 | 0 | 527 | | Not estimable |
| Marret 2007 | 0 | 286 | 0 | 278 | | Not estimable |
| Rouse 2008 | 0 | 1096 | 0 | 1145 | | Not estimable |
| **Subtotal (95% CI)** | | **1917** | | **1950** | | **Not estimable** |
| Total events | 0 | | 0 | | | |
| Heterogeneity: Not applicable | | | | | | |
| Test for overall effect: Not applicable | | | | | | |
| **Total (95% CI)** | | **7317** | | **7345** | **100.0%** | **0.53 [0.26, 1.09]** |
| Total events | 11 | | 21 | | | |
| Heterogeneity: Chi² = 0.09, df = 1 (P = 0.76); I² = 0% | | | | | | |
| Test for overall effect: Z = 1.72 (P = 0.09) | | | | | | |
| Test for subgroup differences: Not applicable | | | | | | |

Risk Ratio
M-H, Fixed, 95% CI

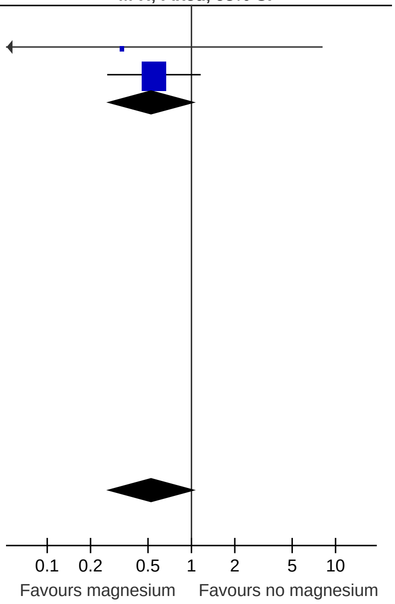


**Figure A1. Forest plot of Comparison: 1 Magnesium sulphate vs. placebo/no treatment, outcome: 1.1 Death**

| | Magnesium sulphate | | Placebo or no treatment | | | Risk Ratio |
|---|---|---|---|---|---|---|
| **Study or Subgroup** | **Events** | **Total** | **Events** | **Total** | **Weight** | **M-H, Fixed, 95% CI** |
| **1.2.1 Indication: pre-eclampsia or eclampsia** | | | | | | |
| Duley 2002 | 4 | 5055 | 5 | 5055 | 100.0% | 0.80 [0.21, 2.98] |
| **Subtotal (95% CI)** | | **5055** | | **5055** | **100.0%** | **0.80 [0.21, 2.98]** |
| Total events | 4 | | 5 | | | |
| Heterogeneity: Not applicable | | | | | | |
| Test for overall effect: Z = 0.33 (P = 0.74) | | | | | | |
| **1.2.2 Indication: fetal neuroprotection** | | | | | | |
| Crowther 2003 | 0 | 535 | 0 | 527 | | Not estimable |
| Marret 2007 | 0 | 286 | 0 | 278 | | Not estimable |
| Rouse 2008 | 0 | 1096 | 0 | 1145 | | Not estimable |
| **Subtotal (95% CI)** | | **1917** | | **1950** | | **Not estimable** |
| Total events | 0 | | 0 | | | |
| Heterogeneity: Not applicable | | | | | | |
| Test for overall effect: Not applicable | | | | | | |
| **Total (95% CI)** | | **6972** | | **7005** | **100.0%** | **0.80 [0.21, 2.98]** |
| Total events | 4 | | 5 | | | |
| Heterogeneity: Not applicable | | | | | | |
| Test for overall effect: Z = 0.33 (P = 0.74) | | | | | | |
| Test for subgroup differences: Not applicable | | | | | | |

Risk Ratio
M-H, Fixed, 95% CI

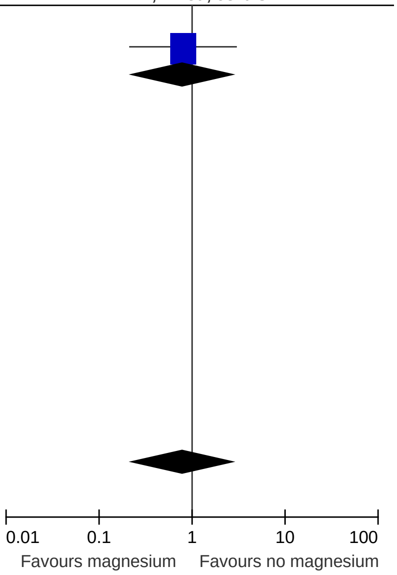


**Figure A2. Forest plot of Comparison: 1 Magnesium sulphate vs. placebo/no treatment, outcome: 1.2 Cardiac arrest**

| | Magnesium sulphate | | Placebo or no treatment | | | Risk Ratio |
|---|---|---|---|---|---|---|
| **Study or Subgroup** | **Events** | **Total** | **Events** | **Total** | **Weight** | **M-H, Fixed, 95% CI** |
| **1.3.1 Indication: pre-eclampsia or eclampsia** | | | | | | |
| Duley 2002 | 5 | 5055 | 2 | 5055 | 100.0% | 2.50 [0.49, 12.88] |
| **Subtotal (95% CI)** | | **5055** | | **5055** | **100.0%** | **2.50 [0.49, 12.88]** |
| Total events | 5 | | 2 | | | |
| Heterogeneity: Not applicable | | | | | | |
| Test for overall effect: Z = 1.10 (P = 0.27) | | | | | | |
| **1.3.2 Indication: fetal neuroprotection** | | | | | | |
| Crowther 2003 | 0 | 535 | 0 | 527 | | Not estimable |
| Marret 2007 | 0 | 286 | 0 | 278 | | Not estimable |
| Rouse 2008 | 0 | 1096 | 0 | 1145 | | Not estimable |
| **Subtotal (95% CI)** | | **1917** | | **1950** | | **Not estimable** |
| Total events | 0 | | 0 | | | |
| Heterogeneity: Not applicable | | | | | | |
| Test for overall effect: Not applicable | | | | | | |
| **Total (95% CI)** | | **6972** | | **7005** | **100.0%** | **2.50 [0.49, 12.88]** |
| Total events | 5 | | 2 | | | |
| Heterogeneity: Not applicable | | | | | | |
| Test for overall effect: Z = 1.10 (P = 0.27) | | | | | | |
| Test for subgroup differences: Not applicable | | | | | | |

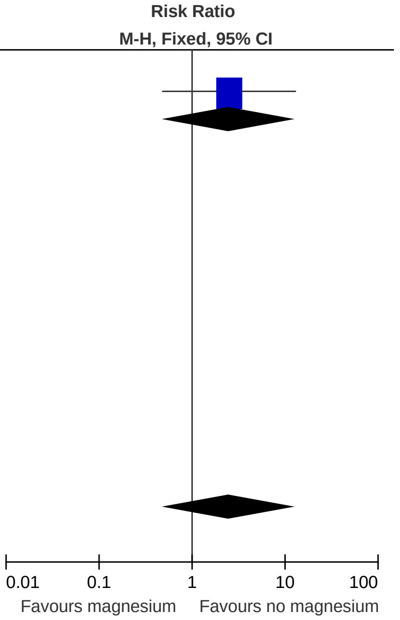


**Figure A3. Forest plot of Comparison: 1 Magnesium sulphate vs. placebo/no treatment, outcome: 1.3 Respiratory arrest**

| | Magnesium sulphate | | Placebo or no treatment | | | Risk Ratio |
|---|---|---|---|---|---|---|
| **Study or Subgroup** | **Events** | **Total** | **Events** | **Total** | **Weight** | **M-H, Fixed, 95% CI** |
| **1.4.1 Indication: pre-eclampsia or eclampsia** | | | | | | |
| Duley 2002 | 317 | 5055 | 118 | 5055 | 72.5% | 2.69 [2.18, 3.31] |
| Witlin 1996 | 1 | 67 | 0 | 68 | 0.3% | 3.04 [0.13, 73.42] |
| **Subtotal (95% CI)** | | **5122** | | **5123** | **72.8%** | **2.69 [2.18, 3.31]** |
| Total events | 318 | | 118 | | | |
| Heterogeneity: Chi² = 0.01, df = 1 (P = 0.94); I² = 0% | | | | | | |
| Test for overall effect: Z = 9.35 (P < 0.00001) | | | | | | |
| **1.4.2 Indication: fetal neuroprotection** | | | | | | |
| Crowther 2003 | 78 | 535 | 28 | 527 | 17.3% | 2.74 [1.81, 4.15] |
| Rouse 2008 | 45 | 1078 | 16 | 1125 | 9.6% | 2.94 [1.67, 5.16] |
| **Subtotal (95% CI)** | | **1613** | | **1652** | **26.9%** | **2.81 [2.01, 3.93]** |
| Total events | 123 | | 44 | | | |
| Heterogeneity: Chi² = 0.04, df = 1 (P = 0.85); I² = 0% | | | | | | |
| Test for overall effect: Z = 6.06 (P < 0.00001) | | | | | | |
| **1.4.3 Indication: tocolysis** | | | | | | |
| Cox 1990 | 8 | 76 | 0 | 80 | 0.3% | 17.88 [1.05, 304.57] |
| **Subtotal (95% CI)** | | **76** | | **80** | **0.3%** | **17.88 [1.05, 304.57]** |
| Total events | 8 | | 0 | | | |
| Heterogeneity: Not applicable | | | | | | |
| Test for overall effect: Z = 1.99 (P = 0.05) | | | | | | |
| **Total (95% CI)** | | **6811** | | **6855** | **100.0%** | **2.77 [2.32, 3.30]** |
| Total events | 449 | | 162 | | | |
| Heterogeneity: Chi² = 1.79, df = 4 (P = 0.77); I² = 0% | | | | | | |
| Test for overall effect: Z = 11.36 (P < 0.00001) | | | | | | |
| Test for subgroup differences: Chi² = 1.74, df = 2 (P = 0.42), I² = 0% | | | | | | |

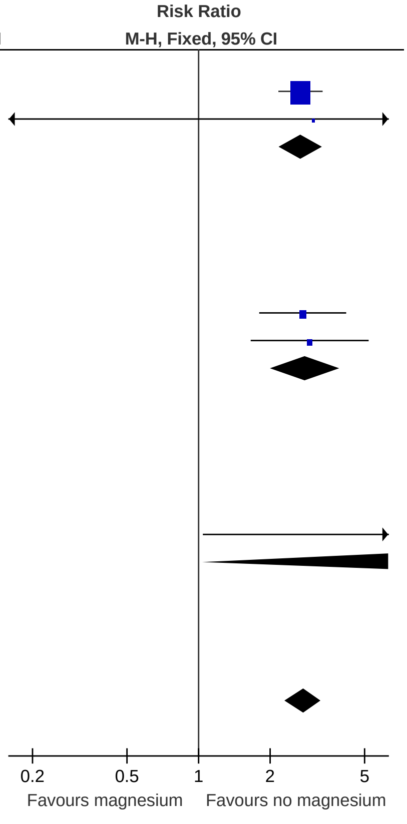


**Figure A4. Forest plot of Comparison: 1 Magnesium sulphate vs. placebo/no treatment, outcome: 1.4 Discontinuation due to adverse effects**

| | Magnesium sulphate | | Placebo or no treatment | | | Risk Ratio | Risk Ratio |
|---|---|---|---|---|---|---|---|
| Study or Subgroup | Events | Total | Events | Total | Weight | M-H, Fixed, 95% CI | M-H, Fixed, 95% CI |
| **1.5.1 Indication: pre-eclampsia or eclampsia** | | | | | | | |
| Coetzee 1998 | 1 | 345 | 0 | 340 | 4.4% | 2.96 [0.12, 72.32] | |
| Duley 2002 | 14 | 5055 | 11 | 5055 | 95.6% | 1.27 [0.58, 2.80] | |
| **Subtotal (95% CI)** | | **5400** | | **5395** | **100.0%** | **1.35 [0.63, 2.88]** | |
| Total events | 15 | | 11 | | | | |
| Heterogeneity: Chi² = 0.25, df = 1 (P = 0.62); I² = 0% | | | | | | | |
| Test for overall effect: Z = 0.77 (P = 0.44) | | | | | | | |
| **Total (95% CI)** | | **5400** | | **5395** | **100.0%** | **1.35 [0.63, 2.88]** | |
| Total events | 15 | | 11 | | | | |
| Heterogeneity: Chi² = 0.25, df = 1 (P = 0.62); I² = 0% | | | | | | | |
| Test for overall effect: Z = 0.77 (P = 0.44) | | | | | | | |
| Test for subgroup differences: Not applicable | | | | | | | |

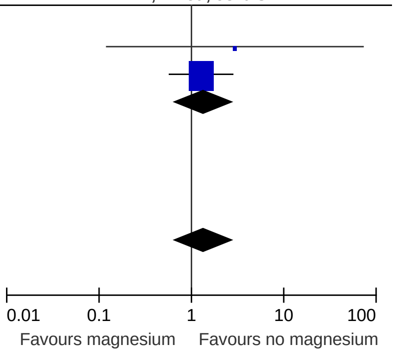


**Figure A5. Forest plot of Comparison: 1 Magnesium sulphate vs. placebo/no treatment, outcome: 1.5 Given calcium gluconate**

| | Magnesium sulphate | | Placebo or no treatment | | | Risk Ratio | Risk Ratio |
|---|---|---|---|---|---|---|---|
| Study or Subgroup | Events | Total | Events | Total | Weight | M-H, Fixed, 95% CI | M-H, Fixed, 95% CI |
| **1.6.1 Indication: pre-eclampsia or eclampsia** | | | | | | | |
| Duley 2002 | 83 | 5055 | 86 | 5055 | 100.0% | 0.97 [0.72, 1.30] | |
| **Subtotal (95% CI)** | | **5055** | | **5055** | **100.0%** | **0.97 [0.72, 1.30]** | |
| Total events | 83 | | 86 | | | | |
| Heterogeneity: Not applicable | | | | | | | |
| Test for overall effect: Z = 0.23 (P = 0.82) | | | | | | | |
| **1.6.2 Indication: fetal neuroprotection** | | | | | | | |
| Crowther 2003 | 0 | 535 | 0 | 527 | | Not estimable | |
| **Subtotal (95% CI)** | | **535** | | **527** | | **Not estimable** | |
| Total events | 0 | | 0 | | | | |
| Heterogeneity: Not applicable | | | | | | | |
| Test for overall effect: Not applicable | | | | | | | |
| **Total (95% CI)** | | **5590** | | **5582** | **100.0%** | **0.97 [0.72, 1.30]** | |
| Total events | 83 | | 86 | | | | |
| Heterogeneity: Not applicable | | | | | | | |
| Test for overall effect: Z = 0.23 (P = 0.82) | | | | | | | |
| Test for subgroup differences: Not applicable | | | | | | | |

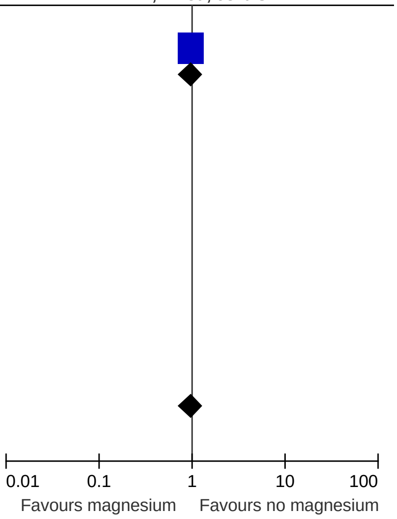


**Figure A6. Forest plot of Comparison: 1 Magnesium sulphate vs. placebo/no treatment, outcome: 1.6 ICU admission**

| | Magnesium sulphate | | Placebo or no treatment | | | Risk Ratio | Risk Ratio |
|---|---|---|---|---|---|---|---|
| Study or Subgroup | Events | Total | Events | Total | Weight | M-H, Random, 95% CI | M-H, Random, 95% CI |
| **1.7.1 Indication: pre-eclampsia or eclampsia** | | | | | | | |
| Duley 2002 | 1201 | 4999 | 228 | 4993 | 31.8% | 5.26 [4.59, 6.03] | |
| **Subtotal (95% CI)** | | **4999** | | **4993** | **31.8%** | **5.26 [4.59, 6.03]** | |
| Total events | 1201 | | 228 | | | | |
| Heterogeneity: Not applicable | | | | | | | |
| Test for overall effect: Z = 23.92 (P < 0.00001) | | | | | | | |
| **1.7.2 Indication: fetal neuroprotection** | | | | | | | |
| Crowther 2003 | 476 | 535 | 199 | 527 | 31.9% | 2.36 [2.10, 2.64] | |
| Rouse 2008 | 833 | 1078 | 140 | 1125 | 31.6% | 6.21 [5.30, 7.27] | |
| **Subtotal (95% CI)** | | **1613** | | **1652** | **63.6%** | **3.82 [1.38, 10.59]** | |
| Total events | 1309 | | 339 | | | | |
| Heterogeneity: Tau² = 0.54; Chi² = 109.57, df = 1 (P < 0.00001); I² = 99% | | | | | | | |
| Test for overall effect: Z = 2.58 (P = 0.01) | | | | | | | |
| **1.7.3 Indication: tocolysis** | | | | | | | |
| Ma 1992 | 11 | 30 | 0 | 35 | 4.6% | 26.71 [1.64, 435.03] | |
| **Subtotal (95% CI)** | | **30** | | **35** | **4.6%** | **26.71 [1.64, 435.03]** | |
| Total events | 11 | | 0 | | | | |
| Heterogeneity: Not applicable | | | | | | | |
| Test for overall effect: Z = 2.31 (P = 0.02) | | | | | | | |
| **Total (95% CI)** | | **6642** | | **6680** | **100.0%** | **4.62 [2.42, 8.83]** | |
| Total events | 2521 | | 567 | | | | |
| Heterogeneity: Tau² = 0.34; Chi² = 146.15, df = 3 (P < 0.00001); I² = 98% | | | | | | | |
| Test for overall effect: Z = 4.64 (P < 0.00001) | | | | | | | |
| Test for subgroup differences: Chi² = 1.68, df = 2 (P = 0.43), I² = 0% | | | | | | | |

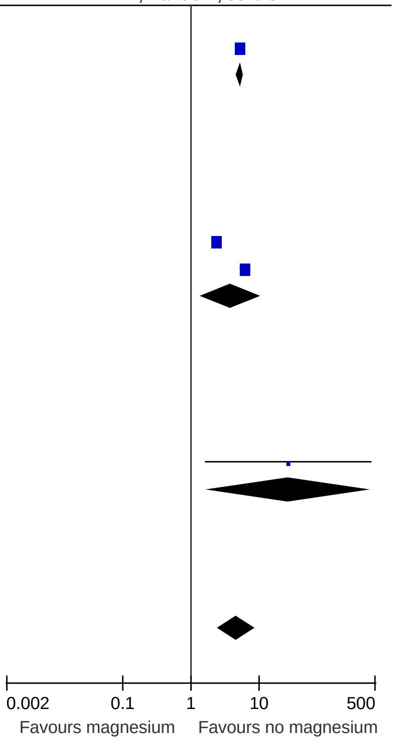


**Figure A7. Forest plot of Comparison: 1 Magnesium sulphate vs. placebo/no treatment, outcome: 1.7 Any adverse effects**

| Study or Subgroup | Magnesium sulphate Events | Magnesium sulphate Total | Placebo or no treatment Events | Placebo or no treatment Total | Weight | Risk Ratio M-H, Fixed, 95% CI |
|---|---|---|---|---|---|---|
| **1.8.1 Indication: pre-eclampsia or eclampsia** | | | | | | |
| Coetzee 1998 | 1 | 345 | 0 | 340 | 0.6% | 2.96 [0.12, 72.32] |
| Duley 2002 | 51 | 4999 | 26 | 4993 | 32.0% | 1.96 [1.22, 3.14] |
| **Subtotal (95% CI)** | | **5344** | | **5333** | **32.6%** | **1.98 [1.24, 3.15]** |
| Total events | 52 | | 26 | | | |
| Heterogeneity: Chi² = 0.06, df = 1 (P = 0.80); I² = 0% | | | | | | |
| Test for overall effect: Z = 2.87 (P = 0.004) | | | | | | |
| **1.8.2 Indication: fetal neuroprotection** | | | | | | |
| Crowther 2003 | 54 | 535 | 51 | 527 | 63.2% | 1.04 [0.73, 1.50] |
| Rouse 2008 | 7 | 1078 | 3 | 1125 | 3.6% | 2.44 [0.63, 9.39] |
| **Subtotal (95% CI)** | | **1613** | | **1652** | **66.8%** | **1.12 [0.79, 1.59]** |
| Total events | 61 | | 54 | | | |
| Heterogeneity: Chi² = 1.42, df = 1 (P = 0.23); I² = 29% | | | | | | |
| Test for overall effect: Z = 0.63 (P = 0.53) | | | | | | |
| **1.8.3 Indication: tocolysis** | | | | | | |
| Cox 1990 | 1 | 76 | 0 | 80 | 0.6% | 3.16 [0.13, 76.30] |
| **Subtotal (95% CI)** | | **76** | | **80** | **0.6%** | **3.16 [0.13, 76.30]** |
| Total events | 1 | | 0 | | | |
| Heterogeneity: Not applicable | | | | | | |
| Test for overall effect: Z = 0.71 (P = 0.48) | | | | | | |
| **Total (95% CI)** | | **7033** | | **7065** | **100.0%** | **1.41 [1.07, 1.86]** |
| Total events | 114 | | 80 | | | |
| Heterogeneity: Chi² = 5.60, df = 4 (P = 0.23); I² = 29% | | | | | | |
| Test for overall effect: Z = 2.44 (P = 0.01) | | | | | | |
| Test for subgroup differences: Chi² = 3.95, df = 2 (P = 0.14), I² = 49.4% | | | | | | |

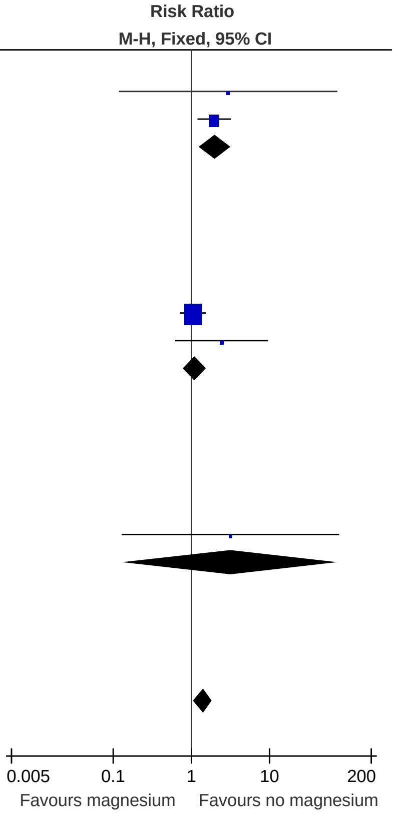


**Figure A8. Forest plot of Comparison: 1 Magnesium sulphate vs. placebo/no treatment, outcome: 1.8 Respiratory depression or other respiratory problems**

| Study or Subgroup | Magnesium sulphate Events | Magnesium sulphate Total | Placebo or no treatment Events | Placebo or no treatment Total | Weight | Risk Ratio M-H, Fixed, 95% CI |
|---|---|---|---|---|---|---|
| **1.9.1 Indication: pre-eclampsia or eclampsia** | | | | | | |
| Coetzee 1998 | 1 | 345 | 0 | 340 | 0.8% | 2.96 [0.12, 72.32] |
| Duley 2002 | 59 | 4999 | 60 | 4993 | 97.5% | 0.98 [0.69, 1.40] |
| **Subtotal (95% CI)** | | **5344** | | **5333** | **98.4%** | **1.00 [0.70, 1.42]** |
| Total events | 60 | | 60 | | | |
| Heterogeneity: Chi² = 0.45, df = 1 (P = 0.50); I² = 0% | | | | | | |
| Test for overall effect: Z = 0.01 (P = 0.99) | | | | | | |
| **1.9.2 Indication: fetal neuroprotection** | | | | | | |
| Marret 2007 | 2 | 286 | 1 | 278 | 1.6% | 1.94 [0.18, 21.32] |
| **Subtotal (95% CI)** | | **286** | | **278** | **1.6%** | **1.94 [0.18, 21.32]** |
| Total events | 2 | | 1 | | | |
| Heterogeneity: Not applicable | | | | | | |
| Test for overall effect: Z = 0.54 (P = 0.59) | | | | | | |
| **Total (95% CI)** | | **5630** | | **5611** | **100.0%** | **1.01 [0.71, 1.44]** |
| Total events | 62 | | 61 | | | |
| Heterogeneity: Chi² = 0.74, df = 2 (P = 0.69); I² = 0% | | | | | | |
| Test for overall effect: Z = 0.08 (P = 0.94) | | | | | | |
| Test for subgroup differences: Chi² = 0.29, df = 1 (P = 0.59), I² = 0% | | | | | | |

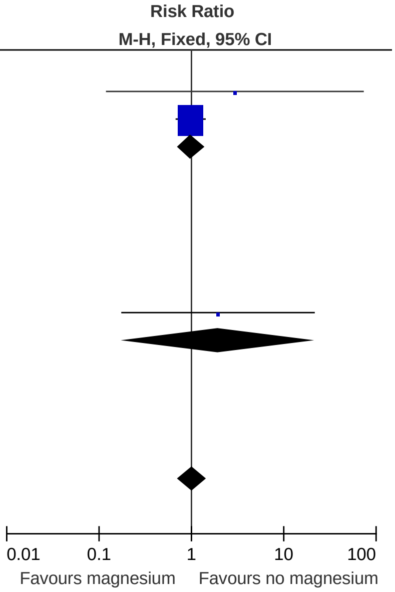


**Figure A9. Forest plot of Comparison: 1 Magnesium sulphate vs. placebo/no treatment, outcome: 1.9 Absent or reduced tendon reflexes**

| Study or Subgroup | Magnesium sulphate Events | Magnesium sulphate Total | Placebo or no treatment Events | Placebo or no treatment Total | Weight | Risk Ratio M-H, Fixed, 95% CI |
|---|---|---|---|---|---|---|
| **1.10.1 Indication: pre-eclampsia or eclampsia** | | | | | | |
| Coetzee 1998 | 1 | 345 | 0 | 340 | 50.2% | 2.96 [0.12, 72.32] |
| Duley 2002 | 4 | 4999 | 0 | 4993 | 49.8% | 8.99 [0.48, 166.92] |
| Livingston 2003 | 0 | 109 | 0 | 113 | | Not estimable |
| **Subtotal (95% CI)** | | **5453** | | **5446** | **100.0%** | **5.96 [0.72, 49.40]** |
| Total events | 5 | | 0 | | | |
| Heterogeneity: Chi² = 0.26, df = 1 (P = 0.61); I² = 0% | | | | | | |
| Test for overall effect: Z = 1.66 (P = 0.10) | | | | | | |
| **Total (95% CI)** | | **5453** | | **5446** | **100.0%** | **5.96 [0.72, 49.40]** |
| Total events | 5 | | 0 | | | |
| Heterogeneity: Chi² = 0.26, df = 1 (P = 0.61); I² = 0% | | | | | | |
| Test for overall effect: Z = 1.66 (P = 0.10) | | | | | | |
| Test for subgroup differences: Not applicable | | | | | | |

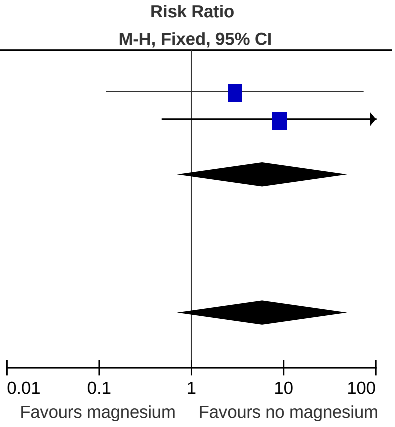


**Figure A10. Forest plot of Comparison: 1 Magnesium sulphate vs. placebo/no treatment, outcome: 1.10 Respiratory depression and absent reflexes**

| Study or Subgroup | Magnesium sulphate Events | Magnesium sulphate Total | Placebo or no treatment Events | Placebo or no treatment Total | Weight | Risk Ratio M-H, Fixed, 95% CI |
|---|---|---|---|---|---|---|
| **1.11.1 Indication: fetal neuroprotection** | | | | | | |
| Crowther 2003 | 77 | 535 | 52 | 527 | 98.1% | 1.46 [1.05, 2.03] |
| Marret 2007 | 3 | 286 | 0 | 278 | 0.9% | 6.80 [0.35, 131.14] |
| **Subtotal (95% CI)** | | **821** | | **805** | **99.1%** | **1.51 [1.09, 2.09]** |
| Total events | 80 | | 52 | | | |
| Heterogeneity: Chi² = 1.04, df = 1 (P = 0.31); I² = 4% | | | | | | |
| Test for overall effect: Z = 2.47 (P = 0.01) | | | | | | |
| **1.11.2 Indication: tocolysis** | | | | | | |
| Cox 1990 | 1 | 76 | 0 | 80 | 0.9% | 3.16 [0.13, 76.30] |
| **Subtotal (95% CI)** | | **76** | | **80** | **0.9%** | **3.16 [0.13, 76.30]** |
| Total events | 1 | | 0 | | | |
| Heterogeneity: Not applicable | | | | | | |
| Test for overall effect: Z = 0.71 (P = 0.48) | | | | | | |
| **Total (95% CI)** | | **897** | | **885** | **100.0%** | **1.52 [1.10, 2.11]** |
| Total events | 81 | | 52 | | | |
| Heterogeneity: Chi² = 1.25, df = 2 (P = 0.53); I² = 0% | | | | | | |
| Test for overall effect: Z = 2.54 (P = 0.01) | | | | | | |
| Test for subgroup differences: Chi² = 0.20, df = 1 (P = 0.65), I² = 0% | | | | | | |

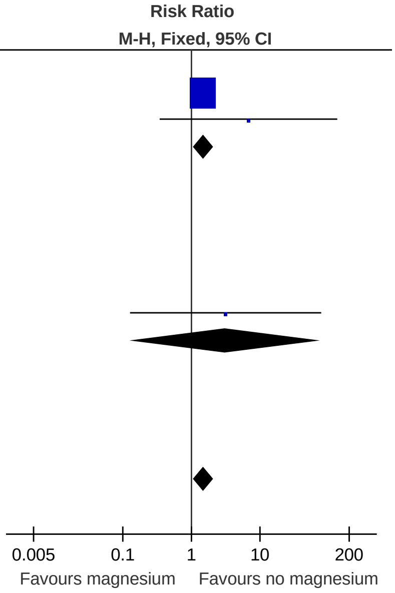


**Figure A11. Forest plot of Comparison: 1 Magnesium sulphate vs. placebo/no treatment, outcome: 1.11 Hypotension**

| Study or Subgroup | Magnesium sulphate Events | Magnesium sulphate Total | Placebo or no treatment Events | Placebo or no treatment Total | Weight | Risk Ratio M-H, Random, 95% CI |
|---|---|---|---|---|---|---|
| **1.13.1 Indication: pre-eclampsia or eclampsia** | | | | | | |
| Duley 2002 | 987 | 4999 | 98 | 4993 | 25.6% | 10.06 [8.20, 12.33] |
| Witlin 1996 | 45 | 67 | 12 | 68 | 20.4% | 3.81 [2.22, 6.53] |
| **Subtotal (95% CI)** | | **5066** | | **5061** | **46.1%** | **6.39 [2.44, 16.74]** |
| Total events | 1032 | | 110 | | | |
| Heterogeneity: Tau² = 0.44; Chi² = 11.17, df = 1 (P = 0.0008); I² = 91% | | | | | | |
| Test for overall effect: Z = 3.78 (P = 0.0002) | | | | | | |
| **1.13.2 Indication: fetal neuroprotection** | | | | | | |
| Crowther 2003 | 393 | 535 | 88 | 527 | 25.7% | 4.40 [3.61, 5.36] |
| Marret 2007 | 23 | 286 | 0 | 278 | 2.9% | 45.69 [2.79, 748.57] |
| Rouse 2008 | 703 | 1078 | 74 | 1125 | 25.4% | 9.91 [7.92, 12.41] |
| **Subtotal (95% CI)** | | **1899** | | **1930** | **53.9%** | **7.55 [3.39, 16.85]** |
| Total events | 1119 | | 162 | | | |
| Heterogeneity: Tau² = 0.35; Chi² = 32.24, df = 2 (P < 0.00001); I² = 94% | | | | | | |
| Test for overall effect: Z = 4.94 (P < 0.00001) | | | | | | |
| **Total (95% CI)** | | **6965** | | **6991** | **100.0%** | **6.94 [4.19, 11.49]** |
| Total events | 2151 | | 272 | | | |
| Heterogeneity: Tau² = 0.25; Chi² = 52.12, df = 4 (P < 0.00001); I² = 92% | | | | | | |
| Test for overall effect: Z = 7.54 (P < 0.00001) | | | | | | |
| Test for subgroup differences: Chi² = 0.07, df = 1 (P = 0.79), I² = 0% | | | | | | |

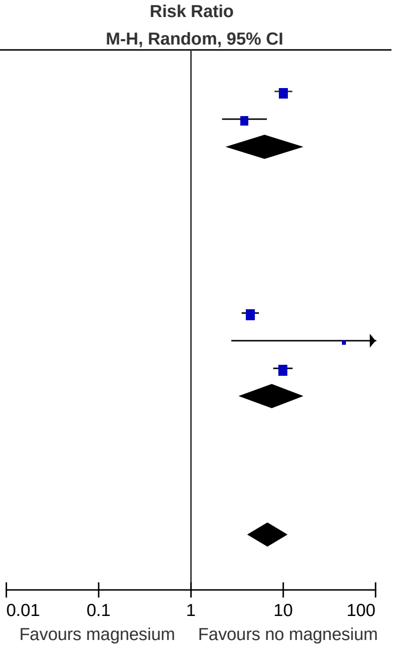


**Figure A12. Forest plot of Comparison: 1 Magnesium sulphate vs. placebo/no treatment, outcome: 1.13 Flushing or warmth**

| Study or Subgroup | Magnesium sulphate Events | Magnesium sulphate Total | Placebo or no treatment Events | Placebo or no treatment Total | Weight | Risk Ratio M-H, Random, 95% CI |
|---|---|---|---|---|---|---|
| **1.14.1 Indication: pre-eclampsia or eclampsia** | | | | | | |
| Duley 2002 | 160 | 4999 | 18 | 4993 | 27.5% | 8.88 [5.46, 14.43] |
| **Subtotal (95% CI)** | | **4999** | | **4993** | **27.5%** | **8.88 [5.46, 14.43]** |
| Total events | 160 | | 18 | | | |
| Heterogeneity: Not applicable | | | | | | |
| Test for overall effect: Z = 8.81 (P < 0.00001) | | | | | | |
| **1.14.2 Indication: fetal neuroprotection** | | | | | | |
| Crowther 2003 | 137 | 535 | 55 | 527 | 29.1% | 2.45 [1.84, 3.28] |
| Marret 2007 | 9 | 286 | 2 | 278 | 15.7% | 4.37 [0.95, 20.06] |
| Rouse 2008 | 166 | 1078 | 19 | 1125 | 27.7% | 9.12 [5.71, 14.55] |
| **Subtotal (95% CI)** | | **1899** | | **1930** | **72.5%** | **4.60 [1.54, 13.73]** |
| Total events | 312 | | 76 | | | |
| Heterogeneity: $Tau^2 = 0.77$; $Chi^2 = 23.55$, df = 2 (P < 0.00001); $I^2 = 92\%$ | | | | | | |
| Test for overall effect: Z = 2.73 (P = 0.006) | | | | | | |
| **Total (95% CI)** | | **6898** | | **6923** | **100.0%** | **5.50 [2.29, 13.22]** |
| Total events | 472 | | 94 | | | |
| Heterogeneity: $Tau^2 = 0.67$; $Chi^2 = 35.32$, df = 3 (P < 0.00001); $I^2 = 92\%$ | | | | | | |
| Test for overall effect: Z = 3.81 (P = 0.0001) | | | | | | |
| Test for subgroup differences: $Chi^2 = 1.16$, df = 1 (P = 0.28), $I^2 = 13.9\%$ | | | | | | |

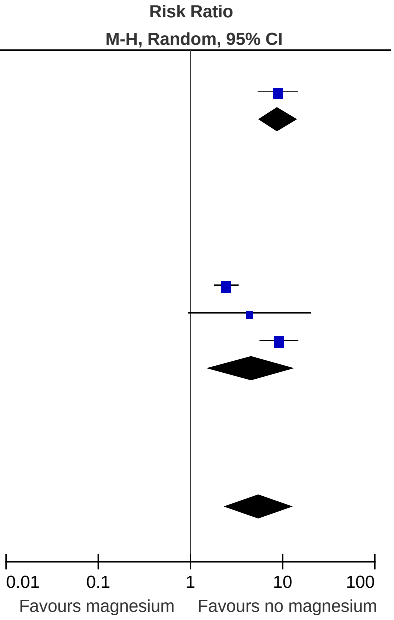


**Figure A13. Forest plot of Comparison: 1 Magnesium sulphate vs. placebo/no treatment, outcome: 1.14 Nausea and/or vomiting**

| Study or Subgroup | Magnesium sulphate Events | Magnesium sulphate Total | Placebo or no treatment Events | Placebo or no treatment Total | Weight | Risk Ratio M-H, Fixed, 95% CI |
|---|---|---|---|---|---|---|
| **1.15.1 Indication: pre-eclampsia or eclampsia** | | | | | | |
| Chen 1995 | 34 | 34 | 0 | 30 | 7.6% | 61.11 [3.91, 955.66] |
| Duley 2002 | 72 | 4999 | 6 | 4993 | 85.5% | 11.99 [5.22, 27.54] |
| **Subtotal (95% CI)** | | **5033** | | **5023** | **93.1%** | **15.97 [7.23, 35.30]** |
| Total events | 106 | | 6 | | | |
| Heterogeneity: $Chi^2 = 1.37$, df = 1 (P = 0.24); $I^2 = 27\%$ | | | | | | |
| Test for overall effect: Z = 6.85 (P < 0.00001) | | | | | | |
| **1.15.2 Indication: tocolysis** | | | | | | |
| Cox 1990 | 6 | 76 | 0 | 80 | 6.9% | 13.68 [0.78, 238.67] |
| **Subtotal (95% CI)** | | **76** | | **80** | **6.9%** | **13.68 [0.78, 238.67]** |
| Total events | 6 | | 0 | | | |
| Heterogeneity: Not applicable | | | | | | |
| Test for overall effect: Z = 1.79 (P = 0.07) | | | | | | |
| **Total (95% CI)** | | **5109** | | **5103** | **100.0%** | **15.81 [7.36, 33.96]** |
| Total events | 112 | | 6 | | | |
| Heterogeneity: $Chi^2 = 1.36$, df = 2 (P = 0.51); $I^2 = 0\%$ | | | | | | |
| Test for overall effect: Z = 7.08 (P < 0.00001) | | | | | | |
| Test for subgroup differences: $Chi^2 = 0.01$, df = 1 (P = 0.92), $I^2 = 0\%$ | | | | | | |

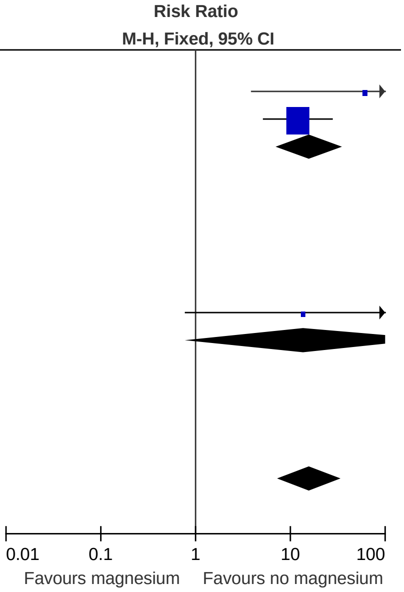


**Figure A14. Forest plot of Comparison: 1 Magnesium sulphate vs. placebo/no treatment, outcome: 1.15 Muscle weakness**

| Study or Subgroup | Magnesium sulphate Events | Magnesium sulphate Total | Placebo or no treatment Events | Placebo or no treatment Total | Weight | Risk Ratio M-H, Fixed, 95% CI |
|---|---|---|---|---|---|---|
| **1.16.1 Indication: pre-eclampsia or eclampsia** | | | | | | |
| Duley 2002 | 20 | 4999 | 9 | 4993 | 15.8% | 2.22 [1.01, 4.87] |
| Witlin 1996 | 1 | 67 | 0 | 68 | 0.9% | 3.04 [0.13, 73.42] |
| **Subtotal (95% CI)** | | **5066** | | **5061** | **16.7%** | **2.26 [1.06, 4.85]** |
| Total events | 21 | | 9 | | | |
| Heterogeneity: Chi² = 0.04, df = 1 (P = 0.85); I² = 0% | | | | | | |
| Test for overall effect: Z = 2.10 (P = 0.04) | | | | | | |
| **1.16.2 Indication: fetal neuroprotection** | | | | | | |
| Crowther 2003 | 119 | 535 | 47 | 527 | 83.3% | 2.49 [1.82, 3.42] |
| **Subtotal (95% CI)** | | **535** | | **527** | **83.3%** | **2.49 [1.82, 3.42]** |
| Total events | 119 | | 47 | | | |
| Heterogeneity: Not applicable | | | | | | |
| Test for overall effect: Z = 5.68 (P < 0.00001) | | | | | | |
| **Total (95% CI)** | | **5601** | | **5588** | **100.0%** | **2.46 [1.83, 3.29]** |
| Total events | 140 | | 56 | | | |
| Heterogeneity: Chi² = 0.09, df = 2 (P = 0.96); I² = 0% | | | | | | |
| Test for overall effect: Z = 6.04 (P < 0.00001) | | | | | | |
| Test for subgroup differences: Chi² = 0.05, df = 1 (P = 0.82), I² = 0% | | | | | | |

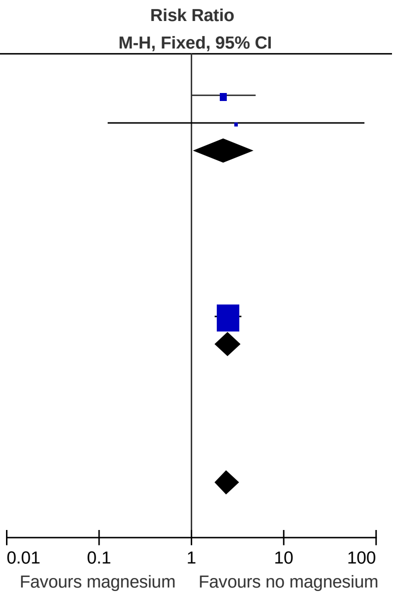


**Figure A15. Forest plot of Comparison: 1 Magnesium sulphate vs. placebo/no treatment, outcome: 1.16 Drowsiness or confusion**

| Study or Subgroup | Magnesium sulphate Events | Magnesium sulphate Total | Placebo or no treatment Events | Placebo or no treatment Total | Weight | Risk Ratio M-H, Fixed, 95% CI |
|---|---|---|---|---|---|---|
| **1.17.1 Indication: pre-eclampsia or eclampsia** | | | | | | |
| Duley 2002 | 36 | 4999 | 17 | 4993 | 94.4% | 2.12 [1.19, 3.76] |
| **Subtotal (95% CI)** | | **4999** | | **4993** | **94.4%** | **2.12 [1.19, 3.76]** |
| Total events | 36 | | 17 | | | |
| Heterogeneity: Not applicable | | | | | | |
| Test for overall effect: Z = 2.55 (P = 0.01) | | | | | | |
| **1.17.2 Indication: fetal neuroprotection** | | | | | | |
| Marret 2007 | 4 | 286 | 1 | 278 | 5.6% | 3.89 [0.44, 34.57] |
| **Subtotal (95% CI)** | | **286** | | **278** | **5.6%** | **3.89 [0.44, 34.57]** |
| Total events | 4 | | 1 | | | |
| Heterogeneity: Not applicable | | | | | | |
| Test for overall effect: Z = 1.22 (P = 0.22) | | | | | | |
| **Total (95% CI)** | | **5285** | | **5271** | **100.0%** | **2.21 [1.27, 3.86]** |
| Total events | 40 | | 18 | | | |
| Heterogeneity: Chi² = 0.28, df = 1 (P = 0.60); I² = 0% | | | | | | |
| Test for overall effect: Z = 2.81 (P = 0.005) | | | | | | |
| Test for subgroup differences: Chi² = 0.28, df = 1 (P = 0.60), I² = 0% | | | | | | |

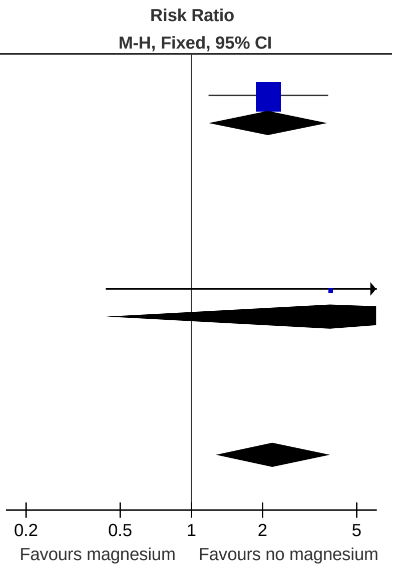


**Figure A16. Forest plot of Comparison: 1 Magnesium sulphate vs. placebo/no treatment, outcome: 1.17 Headache.**

| Study or Subgroup | Magnesium sulphate Events | Magnesium sulphate Total | Placebo or no treatment Events | Placebo or no treatment Total | Weight | Risk Ratio M-H, Random, 95% CI |
|---|---|---|---|---|---|---|
| **1.18.1 Indication: pre-eclampsia or eclampsia** | | | | | | |
| Duley 2002 | 37 | 4999 | 11 | 4993 | 25.8% | 3.36 [1.72, 6.58] |
| **Subtotal (95% CI)** | | **4999** | | **4993** | **25.8%** | **3.36 [1.72, 6.58]** |
| Total events | 37 | | 11 | | | |
| Heterogeneity: Not applicable | | | | | | |
| Test for overall effect: Z = 3.53 (P = 0.0004) | | | | | | |
| **1.18.2 Indication: fetal neuroprotection** | | | | | | |
| Crowther 2003 | 212 | 535 | 99 | 527 | 74.2% | 2.11 [1.72, 2.59] |
| **Subtotal (95% CI)** | | **535** | | **527** | **74.2%** | **2.11 [1.72, 2.59]** |
| Total events | 212 | | 99 | | | |
| Heterogeneity: Not applicable | | | | | | |
| Test for overall effect: Z = 7.10 (P < 0.00001) | | | | | | |
| **Total (95% CI)** | | **5534** | | **5520** | **100.0%** | **2.38 [1.59, 3.56]** |
| Total events | 249 | | 110 | | | |
| Heterogeneity: Tau² = 0.05; Chi² = 1.71, df = 1 (P = 0.19); I² = 42% | | | | | | |
| Test for overall effect: Z = 4.22 (P < 0.0001) | | | | | | |
| Test for subgroup differences: Chi² = 1.68, df = 1 (P = 0.19), I² = 40.6% | | | | | | |

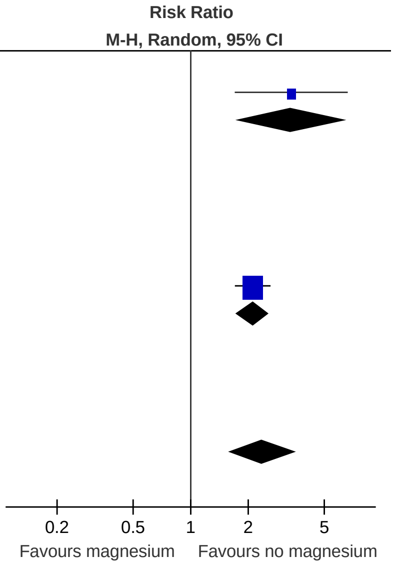


**Figure A17. Forest plot of Comparison: 1 Magnesium sulphate vs. placebo/no treatment, outcome: 1.18 Thirst/mouth dryness.**

| Study or Subgroup | Magnesium sulphate Events | Magnesium sulphate Total | Placebo or no treatment Events | Placebo or no treatment Total | Weight | Risk Ratio M-H, Random, 95% CI |
|---|---|---|---|---|---|---|
| **1.19.1 Indication: pre-eclampsia or eclampsia** | | | | | | |
| Duley 2002 | 37 | 4999 | 10 | 4993 | 32.9% | 3.70 [1.84, 7.42] |
| **Subtotal (95% CI)** | | **4999** | | **4993** | **32.9%** | **3.70 [1.84, 7.42]** |
| Total events | 37 | | 10 | | | |
| Heterogeneity: Not applicable | | | | | | |
| Test for overall effect: Z = 3.67 (P = 0.0002) | | | | | | |
| **1.19.2 Indication: fetal neuroprotection** | | | | | | |
| Crowther 2003 | 83 | 535 | 37 | 527 | 67.1% | 2.21 [1.53, 3.19] |
| **Subtotal (95% CI)** | | **535** | | **527** | **67.1%** | **2.21 [1.53, 3.19]** |
| Total events | 83 | | 37 | | | |
| Heterogeneity: Not applicable | | | | | | |
| Test for overall effect: Z = 4.22 (P < 0.0001) | | | | | | |
| **Total (95% CI)** | | **5534** | | **5520** | **100.0%** | **2.62 [1.63, 4.21]** |
| Total events | 120 | | 47 | | | |
| Heterogeneity: Tau² = 0.05; Chi² = 1.65, df = 1 (P = 0.20); I² = 39% | | | | | | |
| Test for overall effect: Z = 3.96 (P < 0.0001) | | | | | | |
| Test for subgroup differences: Chi² = 1.63, df = 1 (P = 0.20), I² = 38.8% | | | | | | |

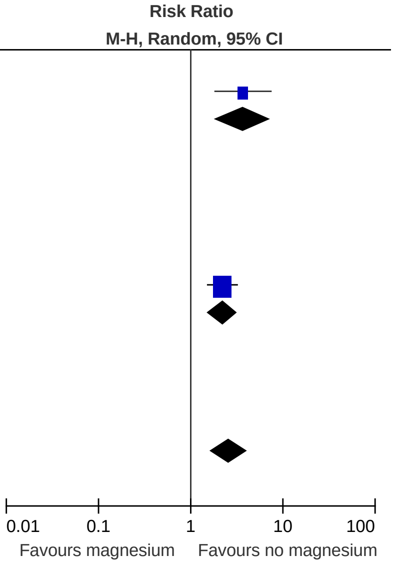


**Figure A18. Forest plot of Comparison: 1 Magnesium sulphate vs. placebo/no treatment, outcome: 1.19 Dizziness**

| Study or Subgroup | Magnesium sulphate Events | Magnesium sulphate Total | Placebo or no treatment Events | Placebo or no treatment Total | Weight | Risk Ratio M-H, Random, 95% CI |
|---|---|---|---|---|---|---|
| **1.20.1 Indication: fetal neuroprotection** | | | | | | |
| Crowther 2003 | 104 | 535 | 29 | 527 | 49.9% | 3.53 [2.38, 5.24] |
| Rouse 2008 | 307 | 1078 | 28 | 1125 | 50.1% | 11.44 [7.84, 16.70] |
| **Subtotal (95% CI)** | | **1613** | | **1652** | **100.0%** | **6.37 [1.96, 20.65]** |
| Total events | 411 | | 57 | | | |
| Heterogeneity: Tau² = 0.68; Chi² = 18.60, df = 1 (P < 0.0001); I² = 95% | | | | | | |
| Test for overall effect: Z = 3.08 (P = 0.002) | | | | | | |
| **Total (95% CI)** | | **1613** | | **1652** | **100.0%** | **6.37 [1.96, 20.65]** |
| Total events | 411 | | 57 | | | |
| Heterogeneity: Tau² = 0.68; Chi² = 18.60, df = 1 (P < 0.0001); I² = 95% | | | | | | |
| Test for overall effect: Z = 3.08 (P = 0.002) | | | | | | |
| Test for subgroup differences: Not applicable | | | | | | |

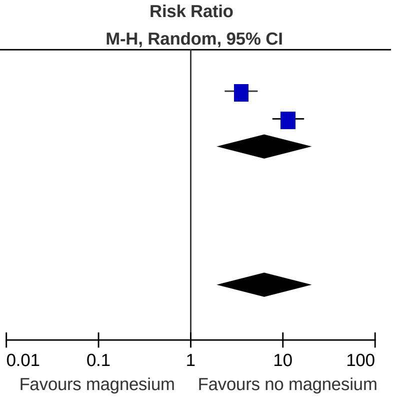


**Figure A19. Forest plot of Comparison: 1 Magnesium sulphate vs. placebo/no treatment, outcome: 1.20 Sweating**

| Study or Subgroup | Magnesium sulphate Events | Magnesium sulphate Total | Placebo or no treatment Events | Placebo or no treatment Total | Weight | Risk Ratio M-H, Random, 95% CI |
|---|---|---|---|---|---|---|
| **1.24.1 Indication: pre-eclampsia or eclampsia** | | | | | | |
| Duley 2002 | 125 | 2719 | 41 | 2720 | 33.3% | 3.05 [2.15, 4.32] |
| **Subtotal (95% CI)** | | **2719** | | **2720** | **33.3%** | **3.05 [2.15, 4.32]** |
| Total events | 125 | | 41 | | | |
| Heterogeneity: Not applicable | | | | | | |
| Test for overall effect: Z = 6.27 (P < 0.00001) | | | | | | |
| **1.24.2 Indication: fetal neuroprotection** | | | | | | |
| Crowther 2003 | 355 | 535 | 39 | 527 | 33.9% | 8.97 [6.59, 12.20] |
| Rouse 2008 | 259 | 1078 | 29 | 1125 | 32.9% | 9.32 [6.41, 13.56] |
| **Subtotal (95% CI)** | | **1613** | | **1652** | **66.7%** | **9.11 [7.18, 11.55]** |
| Total events | 614 | | 68 | | | |
| Heterogeneity: Tau² = 0.00; Chi² = 0.02, df = 1 (P = 0.88); I² = 0% | | | | | | |
| Test for overall effect: Z = 18.20 (P < 0.00001) | | | | | | |
| **Total (95% CI)** | | **4332** | | **4372** | **100.0%** | **6.34 [3.10, 12.98]** |
| Total events | 739 | | 109 | | | |
| Heterogeneity: Tau² = 0.37; Chi² = 26.17, df = 2 (P < 0.00001); I² = 92% | | | | | | |
| Test for overall effect: Z = 5.06 (P < 0.00001) | | | | | | |
| Test for subgroup differences: Chi² = 25.80, df = 1 (P < 0.00001), I² = 96.1% | | | | | | |

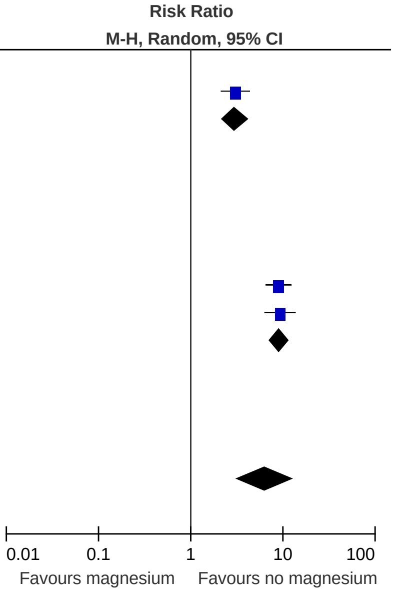


**Figure A20. Forest plot of Comparison: 1 Magnesium sulphate vs. placebo/no treatment, outcome: 1.24 Problems at IV site/arm discomfort.**

| Study or Subgroup | Magnesium sulphate Events | Magnesium sulphate Total | Placebo or no treatment Events | Placebo or no treatment Total | Weight | Risk Ratio M-H, Fixed, 95% CI |
|---|---|---|---|---|---|---|
| **1.26.1 Indication: pre-eclampsia or eclampsia** | | | | | | |
| Chen 1995 | 28 | 34 | 24 | 30 | 0.8% | 1.03 [0.81, 1.30] |
| Coetzee 1998 | 173 | 345 | 165 | 340 | 5.2% | 1.03 [0.89, 1.20] |
| Duley 2002 | 2224 | 4415 | 2082 | 4359 | 65.0% | 1.05 [1.01, 1.10] |
| Livingston 2003 | 30 | 100 | 27 | 110 | 0.8% | 1.22 [0.78, 1.91] |
| Moodley 1994 | 59 | 112 | 56 | 116 | 1.7% | 1.09 [0.84, 1.41] |
| Witlin 1996 | 11 | 67 | 14 | 68 | 0.4% | 0.80 [0.39, 1.63] |
| **Subtotal (95% CI)** | | **5073** | | **5023** | **73.9%** | **1.05 [1.01, 1.10]** |
| Total events | 2525 | | 2368 | | | |
| Heterogeneity: Chi² = 1.19, df = 5 (P = 0.95); I² = 0% | | | | | | |
| Test for overall effect: Z = 2.58 (P = 0.010) | | | | | | |
| **1.26.2 Indication: fetal neuroprotection** | | | | | | |
| Crowther 2003 | 289 | 535 | 290 | 527 | 9.1% | 0.98 [0.88, 1.10] |
| Marret 2007 | 116 | 286 | 96 | 278 | 3.0% | 1.17 [0.95, 1.46] |
| Rouse 2008 | 417 | 1086 | 448 | 1141 | 13.6% | 0.98 [0.88, 1.09] |
| **Subtotal (95% CI)** | | **1907** | | **1946** | **25.7%** | **1.00 [0.93, 1.08]** |
| Total events | 822 | | 834 | | | |
| Heterogeneity: Chi² = 2.46, df = 2 (P = 0.29); I² = 19% | | | | | | |
| Test for overall effect: Z = 0.07 (P = 0.95) | | | | | | |
| **1.26.3 Indication: tocolysis** | | | | | | |
| Cox 1990 | 12 | 76 | 14 | 80 | 0.4% | 0.90 [0.45, 1.82] |
| **Subtotal (95% CI)** | | **76** | | **80** | **0.4%** | **0.90 [0.45, 1.82]** |
| Total events | 12 | | 14 | | | |
| Heterogeneity: Not applicable | | | | | | |
| Test for overall effect: Z = 0.29 (P = 0.77) | | | | | | |
| **Total (95% CI)** | | **7056** | | **7049** | **100.0%** | **1.04 [1.00, 1.08]** |
| Total events | 3359 | | 3216 | | | |
| Heterogeneity: Chi² = 5.39, df = 9 (P = 0.80); I² = 0% | | | | | | |
| Test for overall effect: Z = 2.21 (P = 0.03) | | | | | | |
| Test for subgroup differences: Chi² = 1.59, df = 2 (P = 0.45), I² = 0% | | | | | | |

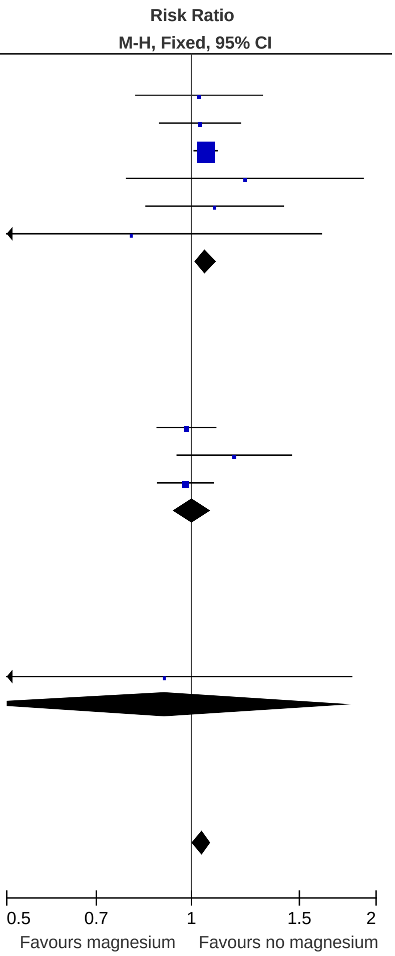


**Figure A21. Forest plot of Comparison: 1 Magnesium sulphate vs. placebo/no treatment, outcome: 1.26 Caesarean**

| Study or Subgroup | Magnesium sulphate Events | Magnesium sulphate Total | Placebo or no treatment Events | Placebo or no treatment Total | Weight | Risk Ratio M-H, Random, 95% CI |
|---|---|---|---|---|---|---|
| **1.27.1 Indication: pre-eclampsia or eclampsia** | | | | | | |
| Duley 2002 | 750 | 4415 | 774 | 4359 | 89.0% | 0.96 [0.87, 1.05] |
| Witlin 1996 | 4 | 67 | 1 | 68 | 0.2% | 4.06 [0.47, 35.38] |
| **Subtotal (95% CI)** | | **4482** | | **4427** | **89.1%** | **1.29 [0.41, 4.09]** |
| Total events | 754 | | 775 | | | |
| Heterogeneity: Tau² = 0.43; Chi² = 1.71, df = 1 (P = 0.19); I² = 42% | | | | | | |
| Test for overall effect: Z = 0.44 (P = 0.66) | | | | | | |
| **1.27.2 Indication: fetal neuroprotection** | | | | | | |
| Crowther 2003 | 86 | 535 | 99 | 527 | 10.7% | 0.86 [0.66, 1.11] |
| Marret 2007 | 2 | 286 | 1 | 278 | 0.1% | 1.94 [0.18, 21.32] |
| **Subtotal (95% CI)** | | **821** | | **805** | **10.9%** | **0.86 [0.67, 1.12]** |
| Total events | 88 | | 100 | | | |
| Heterogeneity: Tau² = 0.00; Chi² = 0.45, df = 1 (P = 0.50); I² = 0% | | | | | | |
| Test for overall effect: Z = 1.10 (P = 0.27) | | | | | | |
| **Total (95% CI)** | | **5303** | | **5232** | **100.0%** | **0.95 [0.87, 1.03]** |
| Total events | 842 | | 875 | | | |
| Heterogeneity: Tau² = 0.00; Chi² = 2.70, df = 3 (P = 0.44); I² = 0% | | | | | | |
| Test for overall effect: Z = 1.21 (P = 0.23) | | | | | | |
| Test for subgroup differences: Chi² = 0.45, df = 1 (P = 0.50), I² = 0% | | | | | | |

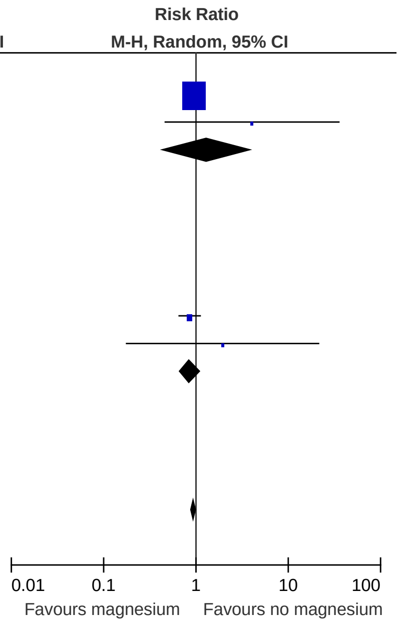


**Figure A22. Forest plot of Comparison: 1 Magnesium sulphate vs. placebo/no treatment, outcome: 1.27 Postpartum haemorrhage**

| | Magnesium sulphate | | Placebo or no treatment | | | Risk Ratio |
|---|---|---|---|---|---|---|
| **Study or Subgroup** | **Events** | **Total** | **Events** | **Total** | **Weight** | **M-H, Fixed, 95% CI** |
| **1.28.1 Indication: pre-eclampsia or eclampsia** | | | | | | |
| Duley 2002 | 32 | 5055 | 33 | 5055 | 89.4% | 0.97 [0.60, 1.57] |
| Livingston 2003 | 0 | 109 | 0 | 113 | | Not estimable |
| Moodley 1994 | 1 | 112 | 1 | 116 | 2.7% | 1.04 [0.07, 16.36] |
| **Subtotal (95% CI)** | | **5276** | | **5284** | **92.1%** | **0.97 [0.60, 1.57]** |
| Total events | 33 | | 34 | | | |
| Heterogeneity: Chi² = 0.00, df = 1 (P = 0.96); I² = 0% | | | | | | |
| Test for overall effect: Z = 0.12 (P = 0.91) | | | | | | |
| **1.28.2 Indication: fetal neuroprotection** | | | | | | |
| Rouse 2008 | 8 | 1086 | 3 | 1141 | 7.9% | 2.80 [0.75, 10.53] |
| **Subtotal (95% CI)** | | **1086** | | **1141** | **7.9%** | **2.80 [0.75, 10.53]** |
| Total events | 8 | | 3 | | | |
| Heterogeneity: Not applicable | | | | | | |
| Test for overall effect: Z = 1.52 (P = 0.13) | | | | | | |
| **Total (95% CI)** | | **6362** | | **6425** | **100.0%** | **1.12 [0.72, 1.74]** |
| Total events | 41 | | 37 | | | |
| Heterogeneity: Chi² = 2.18, df = 2 (P = 0.34); I² = 8% | | | | | | |
| Test for overall effect: Z = 0.49 (P = 0.62) | | | | | | |
| Test for subgroup differences: Chi² = 2.17, df = 1 (P = 0.14), I² = 54.0% | | | | | | |

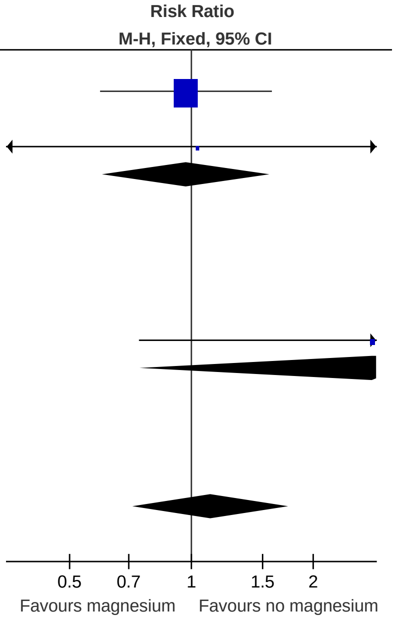


**Figure A23. Forest plot of Comparison: 1 Magnesium sulphate vs. placebo/no treatment, outcome: 1.28 Pulmonary oedema**

| Study or Subgroup | Magnesium sulphate Events | Magnesium sulphate Total | Placebo or no treatment Events | Placebo or no treatment Total | Weight | Risk Ratio M-H, Fixed, 95% CI |
|---|---|---|---|---|---|---|
| **1.29.1 Loading dose only** | | | | | | |
| Marret 2007 | 0 | 286 | 0 | 278 | | Not estimable |
| **Subtotal (95% CI)** | | **286** | | **278** | | **Not estimable** |
| Total events | 0 | | 0 | | | |
| Heterogeneity: Not applicable | | | | | | |
| Test for overall effect: Not applicable | | | | | | |
| **1.29.2 Loading dose and maintenance dose** | | | | | | |
| Coetzee 1998 | 0 | 345 | 1 | 340 | 7.0% | 0.33 [0.01, 8.04] |
| Crowther 2003 | 0 | 535 | 0 | 527 | | Not estimable |
| Duley 2002 | 11 | 5055 | 20 | 5055 | 93.0% | 0.55 [0.26, 1.15] |
| Rouse 2008 | 0 | 1096 | 0 | 1145 | | Not estimable |
| **Subtotal (95% CI)** | | **7031** | | **7067** | **100.0%** | **0.53 [0.26, 1.09]** |
| Total events | 11 | | 21 | | | |
| Heterogeneity: $Chi^2 = 0.09$, df = 1 (P = 0.76); $I^2 = 0\%$ | | | | | | |
| Test for overall effect: Z = 1.72 (P = 0.09) | | | | | | |
| **1.29.3 4 g loading dose and maintenance dose** | | | | | | |
| Coetzee 1998 | 0 | 345 | 1 | 340 | 7.0% | 0.33 [0.01, 8.04] |
| Crowther 2003 | 0 | 535 | 0 | 527 | | Not estimable |
| Duley 2002 | 11 | 5055 | 20 | 5055 | 93.0% | 0.55 [0.26, 1.15] |
| **Subtotal (95% CI)** | | **5935** | | **5922** | **100.0%** | **0.53 [0.26, 1.09]** |
| Total events | 11 | | 21 | | | |
| Heterogeneity: $Chi^2 = 0.09$, df = 1 (P = 0.76); $I^2 = 0\%$ | | | | | | |
| Test for overall effect: Z = 1.72 (P = 0.09) | | | | | | |
| **1.29.4 5-6 g loading dose and maintenance dose** | | | | | | |
| Rouse 2008 | 0 | 1096 | 0 | 1145 | | Not estimable |
| **Subtotal (95% CI)** | | **1096** | | **1145** | | **Not estimable** |
| Total events | 0 | | 0 | | | |
| Heterogeneity: Not applicable | | | | | | |
| Test for overall effect: Not applicable | | | | | | |
| **1.29.5 1 g/hour IV maintenance dose** | | | | | | |
| Coetzee 1998 | 0 | 345 | 1 | 340 | 17.8% | 0.33 [0.01, 8.04] |
| Crowther 2003 | 0 | 535 | 0 | 527 | | Not estimable |
| Duley 2002 | 3 | 2754 | 7 | 2763 | 82.2% | 0.43 [0.11, 1.66] |
| **Subtotal (95% CI)** | | **3634** | | **3630** | **100.0%** | **0.41 [0.12, 1.43]** |
| Total events | 3 | | 8 | | | |
| Heterogeneity: $Chi^2 = 0.02$, df = 1 (P = 0.88); $I^2 = 0\%$ | | | | | | |
| Test for overall effect: Z = 1.40 (P = 0.16) | | | | | | |
| **1.29.6 2-3 g/hour IV maintenance dose** | | | | | | |
| Rouse 2008 | 0 | 1096 | 0 | 1145 | | Not estimable |
| **Subtotal (95% CI)** | | **1096** | | **1145** | | **Not estimable** |
| Total events | 0 | | 0 | | | |
| Heterogeneity: Not applicable | | | | | | |
| Test for overall effect: Not applicable | | | | | | |
| **1.29.7 IM maintenance dose** | | | | | | |
| Duley 2002 | 8 | 2301 | 13 | 2292 | 100.0% | 0.61 [0.25, 1.48] |
| **Subtotal (95% CI)** | | **2301** | | **2292** | **100.0%** | **0.61 [0.25, 1.48]** |
| Total events | 8 | | 13 | | | |
| Heterogeneity: Not applicable | | | | | | |
| Test for overall effect: Z = 1.09 (P = 0.28) | | | | | | |

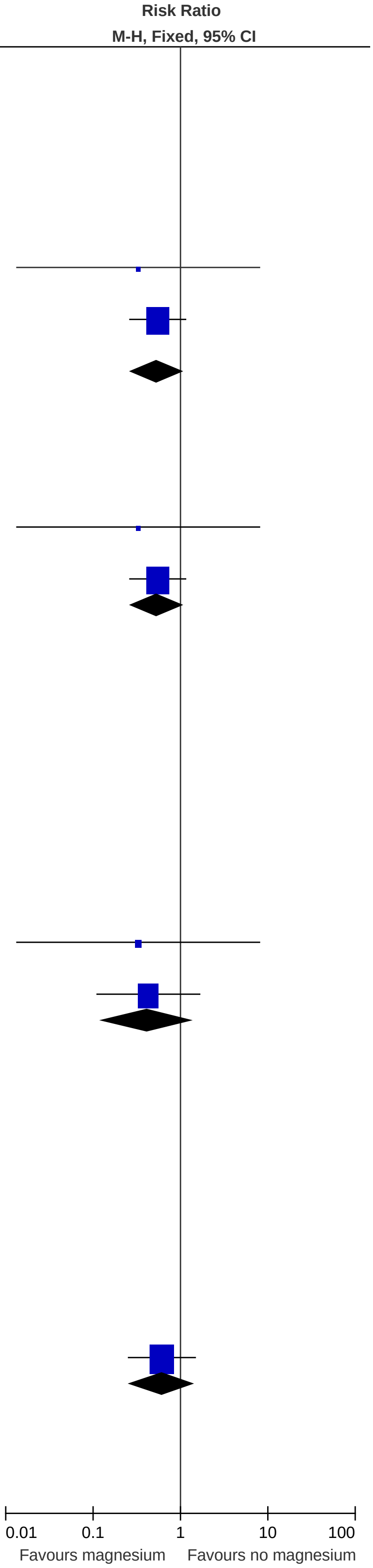


Test for subgroup differences: $Chi^2 = 0.26$, df = 3 (P = 0.97), $I^2 = 0\%$

**Figure A24. Forest plot of Comparison: 1 By dose and route of administration, outcome: 1.29 Death**

| Study or Subgroup | Magnesium sulphate Events | Magnesium sulphate Total | Placebo or no treatment Events | Placebo or no treatment Total | Weight | Risk Ratio M-H, Fixed, 95% CI |
|---|---|---|---|---|---|---|
| **1.30.1 Loading dose only** | | | | | | |
| Marret 2007 | 0 | 286 | 0 | 278 | | Not estimable |
| **Subtotal (95% CI)** | | **286** | | **278** | | **Not estimable** |
| Total events | 0 | | 0 | | | |
| Heterogeneity: Not applicable | | | | | | |
| Test for overall effect: Not applicable | | | | | | |
| **1.30.2 Loading dose and maintenance dose** | | | | | | |
| Crowther 2003 | 0 | 535 | 0 | 527 | | Not estimable |
| Duley 2002 | 4 | 5055 | 5 | 5055 | 100.0% | 0.80 [0.21, 2.98] |
| Rouse 2008 | 0 | 1096 | 0 | 1145 | | Not estimable |
| **Subtotal (95% CI)** | | **6686** | | **6727** | **100.0%** | **0.80 [0.21, 2.98]** |
| Total events | 4 | | 5 | | | |
| Heterogeneity: Not applicable | | | | | | |
| Test for overall effect: Z = 0.33 (P = 0.74) | | | | | | |
| **Total (95% CI)** | | **6972** | | **7005** | **100.0%** | **0.80 [0.21, 2.98]** |
| Total events | 4 | | 5 | | | |
| Heterogeneity: Not applicable | | | | | | |
| Test for overall effect: Z = 0.33 (P = 0.74) | | | | | | |
| Test for subgroup differences: Not applicable | | | | | | |

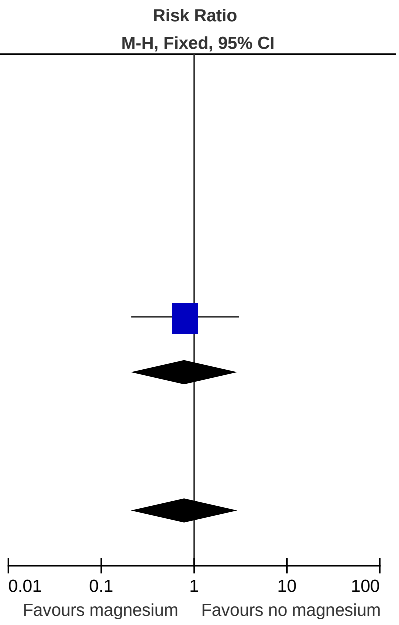


**Figure A25. Forest plot of Comparison: 1 By dose and route of administration, outcome: 1.30 Cardiac arrest**

| Study or Subgroup | Magnesium sulphate Events | Magnesium sulphate Total | Placebo or no treatment Events | Placebo or no treatment Total | Weight | Risk Ratio M-H, Fixed, 95% CI |
|---|---|---|---|---|---|---|
| **1.31.1 Loading dose only** | | | | | | |
| Marret 2007 | 0 | 286 | 0 | 278 | | Not estimable |
| **Subtotal (95% CI)** | | **286** | | **278** | | **Not estimable** |
| Total events | 0 | | 0 | | | |
| Heterogeneity: Not applicable | | | | | | |
| Test for overall effect: Not applicable | | | | | | |
| **1.31.2 Loading dose and maintenance dose** | | | | | | |
| Crowther 2003 | 0 | 535 | 0 | 527 | | Not estimable |
| Duley 2002 | 5 | 5055 | 2 | 5055 | 100.0% | 2.50 [0.49, 12.88] |
| Rouse 2008 | 0 | 1096 | 0 | 1145 | | Not estimable |
| **Subtotal (95% CI)** | | **6686** | | **6727** | **100.0%** | **2.50 [0.49, 12.88]** |
| Total events | 5 | | 2 | | | |
| Heterogeneity: Not applicable | | | | | | |
| Test for overall effect: Z = 1.10 (P = 0.27) | | | | | | |
| **Total (95% CI)** | | **6972** | | **7005** | **100.0%** | **2.50 [0.49, 12.88]** |
| Total events | 5 | | 2 | | | |
| Heterogeneity: Not applicable | | | | | | |
| Test for overall effect: Z = 1.10 (P = 0.27) | | | | | | |
| Test for subgroup differences: Not applicable | | | | | | |

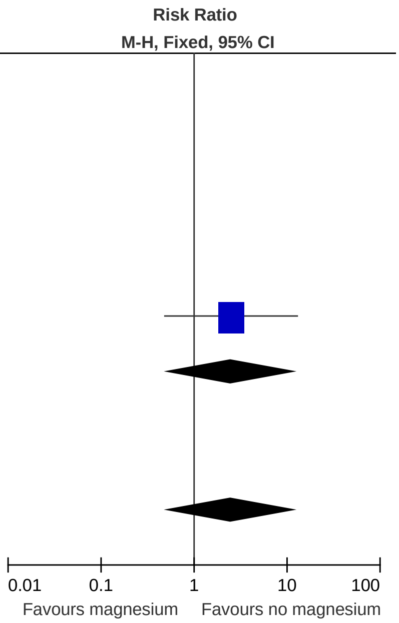


**Figure A26. Forest plot of Comparison: 1 By dose and route of administration, outcome: 1.31 Respiratory arrest**

| Study or Subgroup | Magnesium sulphate Events | Total | Placebo or no treatment Events | Total | Weight | Risk Ratio M-H, Fixed, 95% CI |
|---|---|---|---|---|---|---|
| **1.32.1 Loading dose and maintenance dose** | | | | | | |
| Cox 1990 | 8 | 76 | 0 | 80 | 0.3% | 17.88 [1.05, 304.57] |
| Crowther 2003 | 78 | 535 | 28 | 527 | 17.3% | 2.74 [1.81, 4.15] |
| Duley 2002 | 317 | 5055 | 118 | 5055 | 72.5% | 2.69 [2.18, 3.31] |
| Rouse 2008 | 45 | 1078 | 16 | 1125 | 9.6% | 2.94 [1.67, 5.16] |
| Witlin 1996 | 1 | 67 | 0 | 68 | 0.3% | 3.04 [0.13, 73.42] |
| **Subtotal (95% CI)** | | **6811** | | **6855** | **100.0%** | **2.77 [2.32, 3.30]** |
| Total events | 449 | | 162 | | | |
| Heterogeneity: Chi² = 1.79, df = 4 (P = 0.77); I² = 0% | | | | | | |
| Test for overall effect: Z = 11.36 (P < 0.00001) | | | | | | |
| **1.32.2 4 g loading dose and maintenance dose** | | | | | | |
| Cox 1990 | 8 | 76 | 0 | 80 | 0.3% | 17.88 [1.05, 304.57] |
| Crowther 2003 | 78 | 535 | 28 | 527 | 19.2% | 2.74 [1.81, 4.15] |
| Duley 2002 | 317 | 5055 | 118 | 5055 | 80.4% | 2.69 [2.18, 3.31] |
| **Subtotal (95% CI)** | | **5666** | | **5662** | **100.0%** | **2.75 [2.28, 3.31]** |
| Total events | 403 | | 146 | | | |
| Heterogeneity: Chi² = 1.72, df = 2 (P = 0.42); I² = 0% | | | | | | |
| Test for overall effect: Z = 10.71 (P < 0.00001) | | | | | | |
| **1.32.3 5-6 g loading dose and maintenance dose** | | | | | | |
| Rouse 2008 | 45 | 1078 | 16 | 1125 | 96.9% | 2.94 [1.67, 5.16] |
| Witlin 1996 | 1 | 67 | 0 | 68 | 3.1% | 3.04 [0.13, 73.42] |
| **Subtotal (95% CI)** | | **1145** | | **1193** | **100.0%** | **2.94 [1.69, 5.12]** |
| Total events | 46 | | 16 | | | |
| Heterogeneity: Chi² = 0.00, df = 1 (P = 0.98); I² = 0% | | | | | | |
| Test for overall effect: Z = 3.80 (P = 0.0001) | | | | | | |
| **1.32.4 1 g/hour IV maintenance dose** | | | | | | |
| Crowther 2003 | 78 | 535 | 28 | 527 | 100.0% | 2.74 [1.81, 4.15] |
| **Subtotal (95% CI)** | | **535** | | **527** | **100.0%** | **2.74 [1.81, 4.15]** |
| Total events | 78 | | 28 | | | |
| Heterogeneity: Not applicable | | | | | | |
| Test for overall effect: Z = 4.77 (P < 0.00001) | | | | | | |
| **1.32.5 2-3 g/hour IV maintenance dose** | | | | | | |
| Cox 1990 | 8 | 76 | 0 | 80 | 2.9% | 17.88 [1.05, 304.57] |
| Rouse 2008 | 45 | 1078 | 16 | 1125 | 94.1% | 2.94 [1.67, 5.16] |
| Witlin 1996 | 1 | 67 | 0 | 68 | 3.0% | 3.04 [0.13, 73.42] |
| **Subtotal (95% CI)** | | **1221** | | **1273** | **100.0%** | **3.38 [1.97, 5.78]** |
| Total events | 54 | | 16 | | | |
| Heterogeneity: Chi² = 1.57, df = 2 (P = 0.46); I² = 0% | | | | | | |
| Test for overall effect: Z = 4.43 (P < 0.00001) | | | | | | |

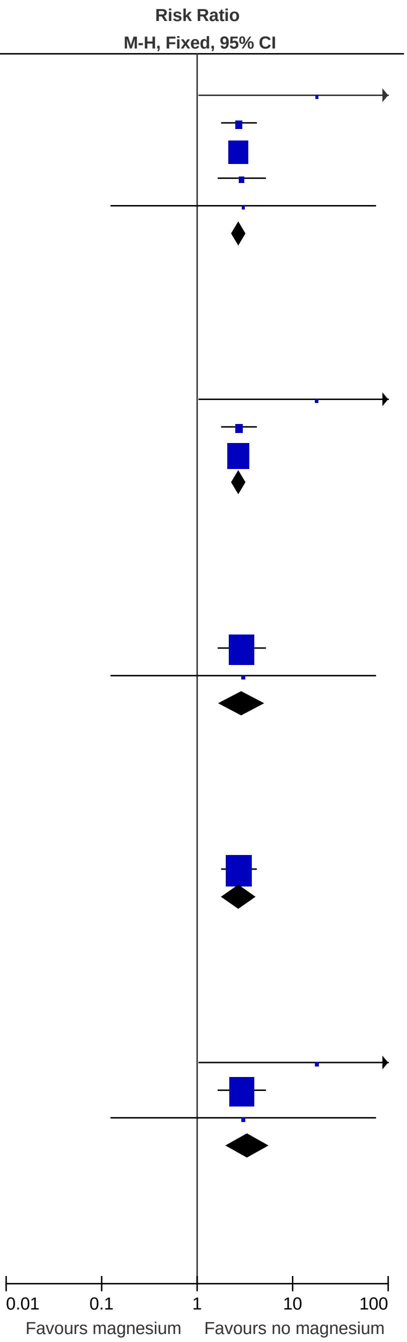


Test for subgroup differences: Chi² = 0.56, df = 4 (P = 0.97), I² = 0%

**Figure A27. Forest plot of Comparison: 1 By dose and route of administration, outcome: 1.32 Discontinuation due to adverse effects**

| Study or Subgroup | Magnesium sulphate Events | Total | Placebo or no treatment Events | Total | Weight | Risk Ratio M-H, Fixed, 95% CI |
|---|---|---|---|---|---|---|
| **1.33.1 4 g loading dose and maintenance dose** | | | | | | |
| Coetzee 1998 | 1 | 345 | 0 | 340 | 4.4% | 2.96 [0.12, 72.32] |
| Duley 2002 | 14 | 5055 | 11 | 5055 | 95.6% | 1.27 [0.58, 2.80] |
| **Subtotal (95% CI)** | | **5400** | | **5395** | **100.0%** | **1.35 [0.63, 2.88]** |
| Total events | 15 | | 11 | | | |
| Heterogeneity: Chi² = 0.25, df = 1 (P = 0.62); I² = 0% | | | | | | |
| Test for overall effect: Z = 0.77 (P = 0.44) | | | | | | |
| **Total (95% CI)** | | **5400** | | **5395** | **100.0%** | **1.35 [0.63, 2.88]** |
| Total events | 15 | | 11 | | | |
| Heterogeneity: Chi² = 0.25, df = 1 (P = 0.62); I² = 0% | | | | | | |
| Test for overall effect: Z = 0.77 (P = 0.44) | | | | | | |
| Test for subgroup differences: Not applicable | | | | | | |

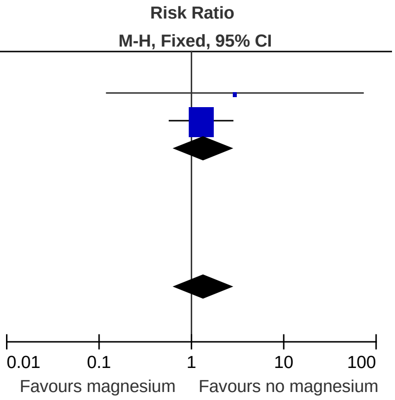


**Figure A28. Forest plot of Comparison: 1 By dose and route of administration, outcome: 1.33 Given calcium gluconate**

| | Magnesium sulphate | | Placebo or no treatment | | | Risk Ratio |
|---|---|---|---|---|---|---|
| Study or Subgroup | Events | Total | Events | Total | Weight | M-H, Random, 95% CI |
| **1.34.1 Loading dose and maintenance dose** | | | | | | |
| Crowther 2003 | 476 | 535 | 199 | 527 | 31.9% | 2.36 [2.10, 2.64] |
| Duley 2002 | 1201 | 4999 | 228 | 4993 | 31.8% | 5.26 [4.59, 6.03] |
| Ma 1992 | 11 | 30 | 0 | 35 | 4.6% | 26.71 [1.64, 435.03] |
| Rouse 2008 | 833 | 1078 | 140 | 1125 | 31.6% | 6.21 [5.30, 7.27] |
| **Subtotal (95% CI)** | | **6642** | | **6680** | **100.0%** | **4.62 [2.42, 8.83]** |
| Total events | 2521 | | 567 | | | |
| Heterogeneity: Tau² = 0.34; Chi² = 146.15, df = 3 (P < 0.00001); I² = 98% | | | | | | |
| Test for overall effect: Z = 4.64 (P < 0.00001) | | | | | | |
| **1.34.2 4 g loading dose and maintenance dose** | | | | | | |
| Crowther 2003 | 476 | 535 | 199 | 527 | 50.1% | 2.36 [2.10, 2.64] |
| Duley 2002 | 1201 | 4999 | 228 | 4993 | 49.9% | 5.26 [4.59, 6.03] |
| **Subtotal (95% CI)** | | **5534** | | **5520** | **100.0%** | **3.52 [1.49, 8.32]** |
| Total events | 1677 | | 427 | | | |
| Heterogeneity: Tau² = 0.38; Chi² = 94.30, df = 1 (P < 0.00001); I² = 99% | | | | | | |
| Test for overall effect: Z = 2.86 (P = 0.004) | | | | | | |
| **1.34.3 5-6 g loading dose and maintenance dose** | | | | | | |
| Ma 1992 | 11 | 30 | 0 | 35 | 2.8% | 26.71 [1.64, 435.03] |
| Rouse 2008 | 833 | 1078 | 140 | 1125 | 97.2% | 6.21 [5.30, 7.27] |
| **Subtotal (95% CI)** | | **1108** | | **1160** | **100.0%** | **6.47 [4.02, 10.40]** |
| Total events | 844 | | 140 | | | |
| Heterogeneity: Tau² = 0.05; Chi² = 1.05, df = 1 (P = 0.30); I² = 5% | | | | | | |
| Test for overall effect: Z = 7.71 (P < 0.00001) | | | | | | |
| **1.34.4 1 g/hour IV maintenance dose** | | | | | | |
| Crowther 2003 | 476 | 535 | 199 | 527 | 50.5% | 2.36 [2.10, 2.64] |
| Duley 2002 | 556 | 2719 | 119 | 2720 | 49.5% | 4.67 [3.86, 5.66] |
| **Subtotal (95% CI)** | | **3254** | | **3247** | **100.0%** | **3.31 [1.59, 6.88]** |
| Total events | 1032 | | 318 | | | |
| Heterogeneity: Tau² = 0.27; Chi² = 43.69, df = 1 (P < 0.00001); I² = 98% | | | | | | |
| Test for overall effect: Z = 3.20 (P = 0.001) | | | | | | |
| **1.34.5 2-3 g/hour IV maintenance dose** | | | | | | |
| Ma 1992 | 11 | 30 | 0 | 35 | 2.8% | 26.71 [1.64, 435.03] |
| Rouse 2008 | 833 | 1078 | 140 | 1125 | 97.2% | 6.21 [5.30, 7.27] |
| **Subtotal (95% CI)** | | **1108** | | **1160** | **100.0%** | **6.47 [4.02, 10.40]** |
| Total events | 844 | | 140 | | | |
| Heterogeneity: Tau² = 0.05; Chi² = 1.05, df = 1 (P = 0.30); I² = 5% | | | | | | |
| Test for overall effect: Z = 7.71 (P < 0.00001) | | | | | | |
| **1.34.6 IM maintenance dose** | | | | | | |
| Duley 2002 | 638 | 2280 | 109 | 2273 | 100.0% | 5.84 [4.80, 7.09] |
| **Subtotal (95% CI)** | | **2280** | | **2273** | **100.0%** | **5.84 [4.80, 7.09]** |
| Total events | 638 | | 109 | | | |
| Heterogeneity: Not applicable | | | | | | |
| Test for overall effect: Z = 17.76 (P < 0.00001) | | | | | | |

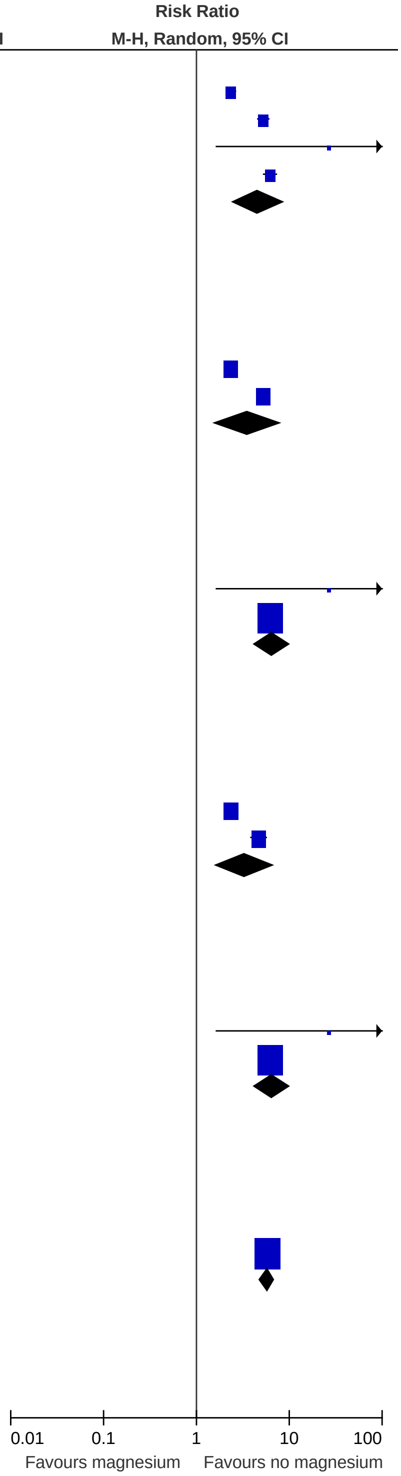


Test for subgroup differences: Chi² = 4.31, df = 5 (P = 0.51), I² = 0%

**Figure A29. Forest plot of Comparison: 1 By dose and route of administration, outcome: 1.34 Any side effects**

| Study or Subgroup | Lower dose Events | Lower dose Total | Higher dose Events | Higher dose Total | Weight | Risk Ratio M-H, Fixed, 95% CI |
|---|---|---|---|---|---|---|
| **2.3.1 (4 g IV LD followed by 2 g/3 h IM MD) versus 'Prichard's' regimen** | | | | | | |
| Malapaka 2011 | 11 | 72 | 19 | 54 | 62.6% | 0.43 [0.23, 0.83] |
| **Subtotal (95% CI)** | | **72** | | **54** | **62.6%** | **0.43 [0.23, 0.83]** |
| Total events | 11 | | 19 | | | |
| Heterogeneity: Not applicable | | | | | | |
| Test for overall effect: Z = 2.50 (P = 0.01) | | | | | | |
| **2.3.2 'Dhaka' regimen versus 'Bhalla' regimen** | | | | | | |
| Shilva 2007 | 3 | 25 | 13 | 25 | 37.4% | 0.23 [0.07, 0.71] |
| **Subtotal (95% CI)** | | **25** | | **25** | **37.4%** | **0.23 [0.07, 0.71]** |
| Total events | 3 | | 13 | | | |
| Heterogeneity: Not applicable | | | | | | |
| Test for overall effect: Z = 2.55 (P = 0.01) | | | | | | |
| **Total (95% CI)** | | **97** | | **79** | **100.0%** | **0.36 [0.20, 0.63]** |
| Total events | 14 | | 32 | | | |
| Heterogeneity: Chi² = 0.92, df = 1 (P = 0.34); I² = 0% | | | | | | |
| Test for overall effect: Z = 3.57 (P = 0.0004) | | | | | | |
| Test for subgroup differences: Chi² = 0.91, df = 1 (P = 0.34), I² = 0% | | | | | | |

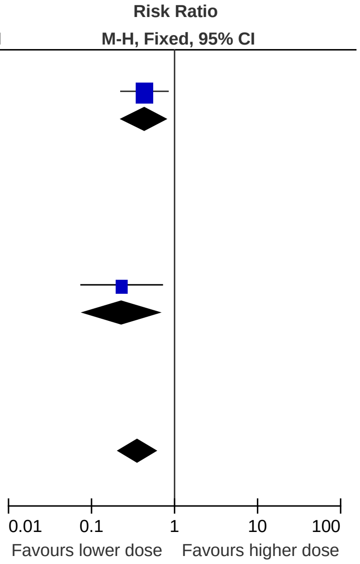


**Figure A30. Forest plot of Comparison: 2 Lower dose vs. higher dose IM regimen, outcome: 2.3 Deferred or skipped doses**

| Study or Subgroup | Lower dose Events | Lower dose Total | Higher dose Events | Higher dose Total | Weight | Risk Ratio M-H, Fixed, 95% CI |
|---|---|---|---|---|---|---|
| **2.6.1 (4 g IV LD followed by 2 g/3 h IM MD) versus 'Prichard's' regimen** | | | | | | |
| Malapaka 2011 | 5 | 72 | 19 | 54 | 73.1% | 0.20 [0.08, 0.50] |
| **Subtotal (95% CI)** | | **72** | | **54** | **73.1%** | **0.20 [0.08, 0.50]** |
| Total events | 5 | | 19 | | | |
| Heterogeneity: Not applicable | | | | | | |
| Test for overall effect: Z = 3.46 (P = 0.0005) | | | | | | |
| **2.6.2 'Dhaka' regimen versus 'Bhalla' regimen** | | | | | | |
| Shilva 2007 | 2 | 25 | 8 | 25 | 26.9% | 0.25 [0.06, 1.06] |
| **Subtotal (95% CI)** | | **25** | | **25** | **26.9%** | **0.25 [0.06, 1.06]** |
| Total events | 2 | | 8 | | | |
| Heterogeneity: Not applicable | | | | | | |
| Test for overall effect: Z = 1.88 (P = 0.06) | | | | | | |
| **Total (95% CI)** | | **97** | | **79** | **100.0%** | **0.21 [0.10, 0.46]** |
| Total events | 7 | | 27 | | | |
| Heterogeneity: Chi² = 0.07, df = 1 (P = 0.79); I² = 0% | | | | | | |
| Test for overall effect: Z = 3.93 (P < 0.0001) | | | | | | |
| Test for subgroup differences: Chi² = 0.07, df = 1 (P = 0.79), I² = 0% | | | | | | |

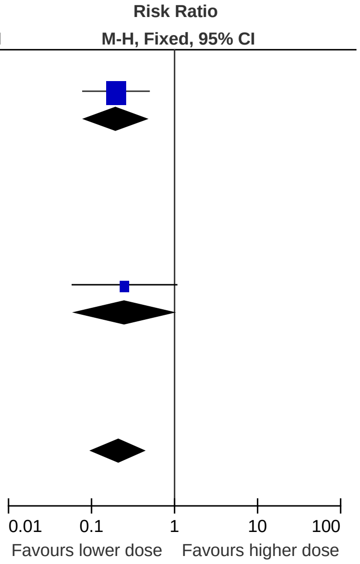


**Figure A31. Forest plot of Comparison: 2 Lower dose vs. higher dose IM regimen, outcome: 2.6 Absent tendon reflexes**

| Study or Subgroup | Intravenous Events | Intravenous Total | Intramuscular Events | Intramuscular Total | Weight | Risk Ratio M-H, Fixed, 95% CI |
|---|---|---|---|---|---|---|
| **3.2.1 (6 g IV LD followed by 2 g/hour MD) versus 'Pritchard's' regimen** | | | | | | |
| Chissell 1994 | 1 | 8 | 0 | 9 | 2.7% | 3.33 [0.15, 71.90] |
| **Subtotal (95% CI)** | | **8** | | **9** | **2.7%** | **3.33 [0.15, 71.90]** |
| Total events | 1 | | 0 | | | |
| Heterogeneity: Not applicable | | | | | | |
| Test for overall effect: Z = 0.77 (P = 0.44) | | | | | | |
| **3.2.2 'Springfusor pump' IV versus 'Standard' IM regimen** | | | | | | |
| Mundle 2009 | 24 | 150 | 17 | 150 | 97.3% | 1.41 [0.79, 2.52] |
| **Subtotal (95% CI)** | | **150** | | **150** | **97.3%** | **1.41 [0.79, 2.52]** |
| Total events | 24 | | 17 | | | |
| Heterogeneity: Not applicable | | | | | | |
| Test for overall effect: Z = 1.17 (P = 0.24) | | | | | | |
| **Total (95% CI)** | | **158** | | **159** | **100.0%** | **1.46 [0.83, 2.58]** |
| Total events | 25 | | 17 | | | |
| Heterogeneity: Chi² = 0.29, df = 1 (P = 0.59); I² = 0% | | | | | | |
| Test for overall effect: Z = 1.32 (P = 0.19) | | | | | | |
| Test for subgroup differences: Chi² = 0.29, df = 1 (P = 0.59), I² = 0% | | | | | | |

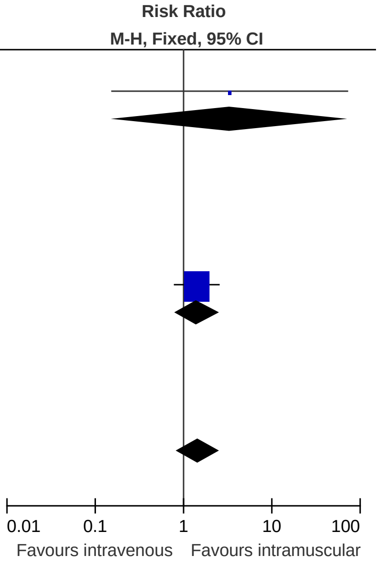


**Figure A32. Forest plot of Comparison: 3 IV maintenance vs. IM maintenance, outcome: 3.2 Discontinuation or modification of treatment**

| Study or Subgroup | Intravenous Events | Intravenous Total | Intramuscular Events | Intramuscular Total | Weight | Risk Ratio M-H, Random, 95% CI |
|---|---|---|---|---|---|---|
| **3.3.1 (4 g IV LD followed by 0.75 g/hour IV MD) versus 'Pritchard's' regimen** | | | | | | |
| Bhattacharjee 2011 | 0 | 67 | 2 | 70 | 50.5% | 0.21 [0.01, 4.27] |
| **Subtotal (95% CI)** | | **67** | | **70** | **50.5%** | **0.21 [0.01, 4.27]** |
| Total events | 0 | | 2 | | | |
| Heterogeneity: Not applicable | | | | | | |
| Test for overall effect: Z = 1.02 (P = 0.31) | | | | | | |
| **3.3.2 (6 g IV LD followed by 2 g/hour MD) versus 'Pritchard's' regimen** | | | | | | |
| Chissell 1994 | 1 | 8 | 0 | 9 | 49.5% | 3.33 [0.15, 71.90] |
| **Subtotal (95% CI)** | | **8** | | **9** | **49.5%** | **3.33 [0.15, 71.90]** |
| Total events | 1 | | 0 | | | |
| Heterogeneity: Not applicable | | | | | | |
| Test for overall effect: Z = 0.77 (P = 0.44) | | | | | | |
| **Total (95% CI)** | | **75** | | **79** | **100.0%** | **0.82 [0.05, 12.56]** |
| Total events | 1 | | 2 | | | |
| Heterogeneity: Tau² = 1.46; Chi² = 1.60, df = 1 (P = 0.21); I² = 38% | | | | | | |
| Test for overall effect: Z = 0.14 (P = 0.89) | | | | | | |
| Test for subgroup differences: Chi² = 1.59, df = 1 (P = 0.21), I² = 37.1% | | | | | | |

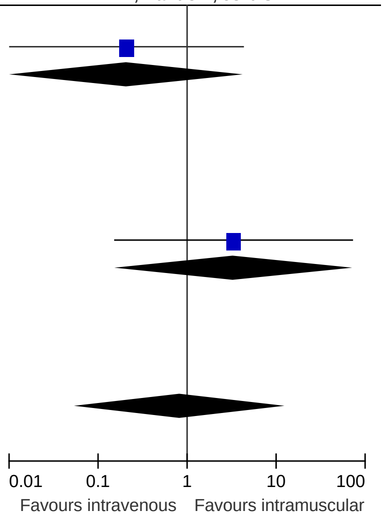


**Figure A33. Forest plot of Comparison: 3 IV maintenance vs. IM maintenance regimen, outcome: 3.3 Clinical signs of toxicity**

| Study or Subgroup | Intravenous Events | Intravenous Total | Intramuscular Events | Intramuscular Total | Weight | Risk Ratio M-H, Fixed, 95% CI |
|---|---|---|---|---|---|---|
| **3.5.1 (4 g IV LD followed by 0.75 g/hour IV MD) versus 'Pritchard's' regimen** | | | | | | |
| Bhattacharjee 2011 | 39 | 67 | 41 | 70 | 93.4% | 0.99 [0.75, 1.32] |
| **Subtotal (95% CI)** | | **67** | | **70** | **93.4%** | **0.99 [0.75, 1.32]** |
| Total events | 39 | | 41 | | | |
| Heterogeneity: Not applicable | | | | | | |
| Test for overall effect: Z = 0.04 (P = 0.97) | | | | | | |
| **3.5.2 (6 g IV LD followed by 2 g/hour MD) versus 'Pritchard's' regimen** | | | | | | |
| Chissell 1994 | 4 | 8 | 3 | 9 | 6.6% | 1.50 [0.47, 4.76] |
| **Subtotal (95% CI)** | | **8** | | **9** | **6.6%** | **1.50 [0.47, 4.76]** |
| Total events | 4 | | 3 | | | |
| Heterogeneity: Not applicable | | | | | | |
| Test for overall effect: Z = 0.69 (P = 0.49) | | | | | | |
| **Total (95% CI)** | | **75** | | **79** | **100.0%** | **1.03 [0.78, 1.35]** |
| Total events | 43 | | 44 | | | |
| Heterogeneity: Chi² = 0.47, df = 1 (P = 0.50); I² = 0% | | | | | | |
| Test for overall effect: Z = 0.19 (P = 0.85) | | | | | | |
| Test for subgroup differences: Chi² = 0.46, df = 1 (P = 0.50), I² = 0% | | | | | | |

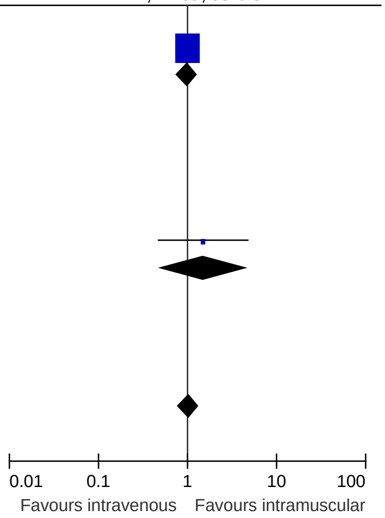


**Figure A34. Forest plot of Comparison: 3 IV maintenance vs. IM maintenance regimen, outcome: 3.5 Caesarean**

| Study or Subgroup | Short Events | Short Total | Standard Events | Standard Total | Weight | Risk Ratio M-H, Fixed, 95% CI |
|---|---|---|---|---|---|---|
| **4.1.1 Short: 12 hours** | | | | | | |
| Ehrenberg 2006 | 0 | 101 | 0 | 95 | | Not estimable |
| **Subtotal (95% CI)** | | **101** | | **95** | | **Not estimable** |
| Total events | 0 | | 0 | | | |
| Heterogeneity: Not applicable | | | | | | |
| Test for overall effect: Not applicable | | | | | | |
| **4.1.2 Short: based on clinical criteria** | | | | | | |
| Suneja 2008 | 2 | 30 | 8 | 30 | 100.0% | 0.25 [0.06, 1.08] |
| **Subtotal (95% CI)** | | **30** | | **30** | **100.0%** | **0.25 [0.06, 1.08]** |
| Total events | 2 | | 8 | | | |
| Heterogeneity: Not applicable | | | | | | |
| Test for overall effect: Z = 1.86 (P = 0.06) | | | | | | |
| **Total (95% CI)** | | **131** | | **125** | **100.0%** | **0.25 [0.06, 1.08]** |
| Total events | 2 | | 8 | | | |
| Heterogeneity: Not applicable | | | | | | |
| Test for overall effect: Z = 1.86 (P = 0.06) | | | | | | |
| Test for subgroup differences: Not applicable | | | | | | |

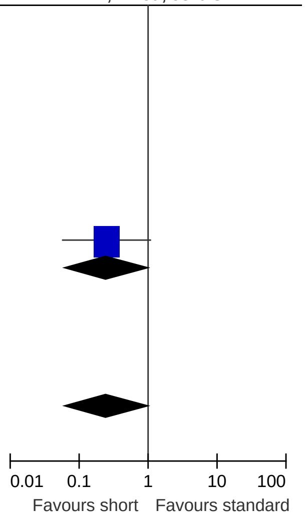


**Figure A35. Forest plot of Comparison: 4 Short vs. Standard (24 hour) postpartum maintenance therapy, outcome: 6.1 Toxicity**

| Study or Subgroup | Lower dose Events | Lower dose Total | Higher dose Events | Higher dose Total | Weight | Risk Ratio M-H, Random, 95% CI |
|---|---|---|---|---|---|---|
| **5.2.1 (4 g LD followed by 2 g/hour MD) versus (6 g LD followed by at least 2 g/hour MD)** | | | | | | |
| Behrad 2003 | 21 | 50 | 18 | 50 | 44.9% | 1.17 [0.71, 1.91] |
| **Subtotal (95% CI)** | | **50** | | **50** | **44.9%** | **1.17 [0.71, 1.91]** |
| Total events | 21 | | 18 | | | |
| Heterogeneity: Not applicable | | | | | | |
| Test for overall effect: Z = 0.61 (P = 0.54) | | | | | | |
| **5.2.2 (4 g LD followed by 2 g/hour MD) versus (4 g LD followed by 5 g/hour MD)** | | | | | | |
| Terrone 2000 | 44 | 70 | 25 | 78 | 55.1% | 1.96 [1.35, 2.84] |
| **Subtotal (95% CI)** | | **70** | | **78** | **55.1%** | **1.96 [1.35, 2.84]** |
| Total events | 44 | | 25 | | | |
| Heterogeneity: Not applicable | | | | | | |
| Test for overall effect: Z = 3.57 (P = 0.0004) | | | | | | |
| **Total (95% CI)** | | **120** | | **128** | **100.0%** | **1.55 [0.94, 2.58]** |
| Total events | 65 | | 43 | | | |
| Heterogeneity: Tau² = 0.09; Chi² = 2.73, df = 1 (P = 0.10); I² = 63% | | | | | | |
| Test for overall effect: Z = 1.70 (P = 0.09) | | | | | | |
| Test for subgroup differences: Chi² = 2.73, df = 1 (P = 0.10), I² = 63.4% | | | | | | |

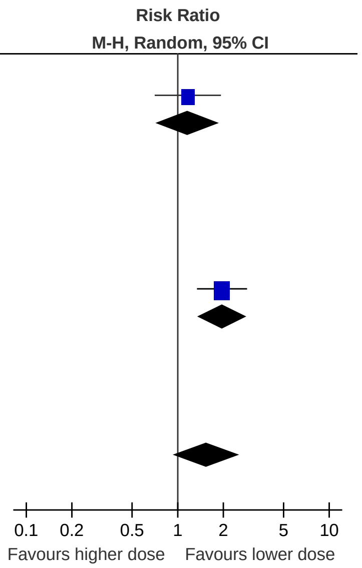


**Figure A36. Forest plot of Comparison: 5 Lower dose vs. higher dose IV regimen, outcome: 5.2 No side effects**

| Study or Subgroup | Lower dose Events | Lower dose Total | Higher dose Events | Higher dose Total | Weight | Risk Ratio M-H, Random, 95% CI |
|---|---|---|---|---|---|---|
| **5.3.1 (4 g LD followed by 2 g/hour MD) versus (6 g LD followed by at least 2 g/hour MD)** | | | | | | |
| Behrad 2003 | 13 | 50 | 15 | 50 | 44.0% | 0.87 [0.46, 1.63] |
| **Subtotal (95% CI)** | | **50** | | **50** | **44.0%** | **0.87 [0.46, 1.63]** |
| Total events | 13 | | 15 | | | |
| Heterogeneity: Not applicable | | | | | | |
| Test for overall effect: Z = 0.44 (P = 0.66) | | | | | | |
| **5.3.2 (4 g LD followed by 2 g/hour MD) versus (4 g LD followed by 5 g/hour MD)** | | | | | | |
| Terrone 2000 | 17 | 70 | 41 | 78 | 56.0% | 0.46 [0.29, 0.73] |
| **Subtotal (95% CI)** | | **70** | | **78** | **56.0%** | **0.46 [0.29, 0.73]** |
| Total events | 17 | | 41 | | | |
| Heterogeneity: Not applicable | | | | | | |
| Test for overall effect: Z = 3.26 (P = 0.001) | | | | | | |
| **Total (95% CI)** | | **120** | | **128** | **100.0%** | **0.61 [0.33, 1.12]** |
| Total events | 30 | | 56 | | | |
| Heterogeneity: Tau² = 0.12; Chi² = 2.48, df = 1 (P = 0.12); I² = 60% | | | | | | |
| Test for overall effect: Z = 1.59 (P = 0.11) | | | | | | |
| Test for subgroup differences: Chi² = 2.48, df = 1 (P = 0.12), I² = 59.6% | | | | | | |

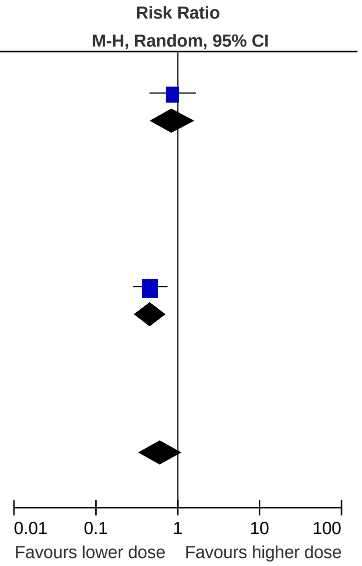


**Figure A36. Forest plot of Comparison: 5 Lower dose vs. higher dose IV regimen, outcome: 5.3 Flushing**

| Study or Subgroup | Lower dose Events | Lower dose Total | Higher dose Events | Higher dose Total | Weight | Risk Ratio M-H, Fixed, 95% CI |
|---|---|---|---|---|---|---|
| **5.4.1 (4 g LD followed by 2 g/hour MD) versus (6 g LD followed by at least 2 g/hour MD)** | | | | | | |
| Behrad 2003 | 4 | 50 | 5 | 50 | 20.9% | 0.80 [0.23, 2.81] |
| **Subtotal (95% CI)** | | **50** | | **50** | **20.9%** | **0.80 [0.23, 2.81]** |
| Total events | 4 | | 5 | | | |
| Heterogeneity: Not applicable | | | | | | |
| Test for overall effect: Z = 0.35 (P = 0.73) | | | | | | |
| **5.4.2 (4 g LD followed by 2 g/hour MD) versus (4 g LD followed by 5 g/hour MD)** | | | | | | |
| Terrone 2000 | 9 | 70 | 20 | 78 | 79.1% | 0.50 [0.24, 1.03] |
| **Subtotal (95% CI)** | | **70** | | **78** | **79.1%** | **0.50 [0.24, 1.03]** |
| Total events | 9 | | 20 | | | |
| Heterogeneity: Not applicable | | | | | | |
| Test for overall effect: Z = 1.89 (P = 0.06) | | | | | | |
| **Total (95% CI)** | | **120** | | **128** | **100.0%** | **0.56 [0.30, 1.05]** |
| Total events | 13 | | 25 | | | |
| Heterogeneity: Chi² = 0.40, df = 1 (P = 0.53); I² = 0% | | | | | | |
| Test for overall effect: Z = 1.81 (P = 0.07) | | | | | | |
| Test for subgroup differences: Chi² = 0.40, df = 1 (P = 0.53), I² = 0% | | | | | | |

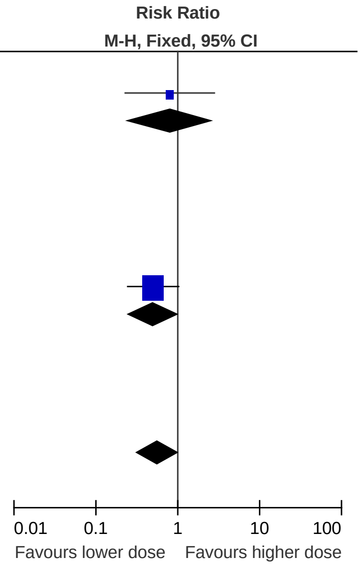


**Figure A38. Forest plot of Comparison: 5 Lower dose vs. higher dose IV regimen, outcome: 5.5 Headache**

| Study or Subgroup | Lower dose Events | Lower dose Total | Higher dose Events | Higher dose Total | Weight | Risk Ratio M-H, Fixed, 95% CI |
|---|---|---|---|---|---|---|
| **5.6.1 (4 g LD followed by 2 g/hour MD) versus (6 g LD followed by at least 2 g/hour MD)** | | | | | | |
| Behrad 2003 | 21 | 50 | 16 | 50 | 54.7% | 1.31 [0.78, 2.21] |
| **Subtotal (95% CI)** | | **50** | | **50** | **54.7%** | **1.31 [0.78, 2.21]** |
| Total events | 21 | | 16 | | | |
| Heterogeneity: Not applicable | | | | | | |
| Test for overall effect: Z = 1.03 (P = 0.30) | | | | | | |
| **5.6.2 (4 g LD followed by 2 g/hour MD) versus (4 g LD followed by 5 g/hour MD)** | | | | | | |
| Terrone 2000 | 11 | 70 | 14 | 78 | 45.3% | 0.88 [0.43, 1.80] |
| **Subtotal (95% CI)** | | **70** | | **78** | **45.3%** | **0.88 [0.43, 1.80]** |
| Total events | 11 | | 14 | | | |
| Heterogeneity: Not applicable | | | | | | |
| Test for overall effect: Z = 0.36 (P = 0.72) | | | | | | |
| **Total (95% CI)** | | **120** | | **128** | **100.0%** | **1.11 [0.73, 1.70]** |
| Total events | 32 | | 30 | | | |
| Heterogeneity: Chi² = 0.81, df = 1 (P = 0.37); I² = 0% | | | | | | |
| Test for overall effect: Z = 0.50 (P = 0.61) | | | | | | |
| Test for subgroup differences: Chi² = 0.80, df = 1 (P = 0.37), I² = 0% | | | | | | |

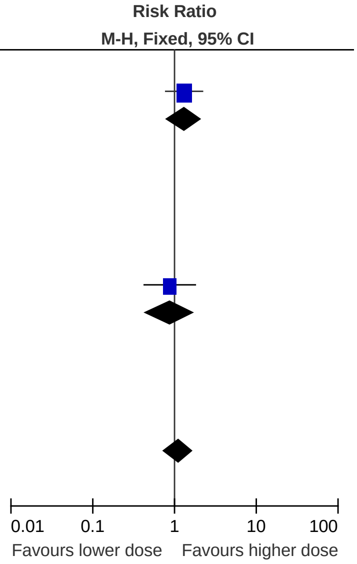


**Figure A39. Forest plot of Comparison: 5 Lower dose vs. higher dose IV regimen, outcome: 5.6 Caesarean**

| Study or Subgroup | Lower dose Events | Lower dose Total | Higher dose Events | Higher dose Total | Weight | Risk Ratio M-H, Fixed, 95% CI |
|---|---|---|---|---|---|---|
| **5.7.1 (4 g LD followed by 2 g/hour MD) versus (6 g LD followed by at least 2 g/hour MD)** | | | | | | |
| Behrad 2003 | 0 | 50 | 0 | 50 | | Not estimable |
| **Subtotal (95% CI)** | | **50** | | **50** | | **Not estimable** |
| Total events | 0 | | 0 | | | |
| Heterogeneity: Not applicable | | | | | | |
| Test for overall effect: Not applicable | | | | | | |
| **5.7.2 (4 g LD followed by 2 g/hour MD) versus (4 g LD followed by 5 g/hour MD)** | | | | | | |
| Terrone 2000 | 1 | 78 | 5 | 82 | 100.0% | 0.21 [0.03, 1.76] |
| **Subtotal (95% CI)** | | **78** | | **82** | **100.0%** | **0.21 [0.03, 1.76]** |
| Total events | 1 | | 5 | | | |
| Heterogeneity: Not applicable | | | | | | |
| Test for overall effect: Z = 1.44 (P = 0.15) | | | | | | |
| **Total (95% CI)** | | **128** | | **132** | **100.0%** | **0.21 [0.03, 1.76]** |
| Total events | 1 | | 5 | | | |
| Heterogeneity: Not applicable | | | | | | |
| Test for overall effect: Z = 1.44 (P = 0.15) | | | | | | |
| Test for subgroup differences: Not applicable | | | | | | |

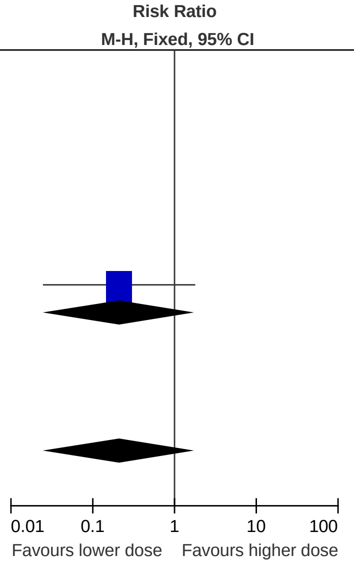


**Figure A40. Forest plot of Comparison: 5 Lower dose vs. higher dose IV regimen, outcome: 5.7 Pulmonary oedema**
