# Supplementary material for: Maternal adverse effects of different antenatal magnesium sulphate regimens for improving maternal and infant outcomes: a systematic review
Source: BMC Pregnancy Childbirth. 2013 Oct 21;13:195. doi: 10.1186/1471-2393-13-195 (PMC4015216; doi:10.1186/1471-2393-13-195)
Supplement: Additional file 6 — Comparison of case series and randomised trial adverse effect estimates. [file 1471-2393-13-195-S6.pdf]

Comparison of case series and randomised trial adverse effect estimates

	Case series				Randomised controlled trials			
**Outcome**	**Mean; Median***	**Range (%)**	**Participants**	**Studies**	**Mean; Median***	**Range (%)**	**Participants**	**Studies**
Death	0.14%; 0	(0-0.14)	285	3 [38,46,62]	0.04%; 0	(0-0.22)	7317	5 [2,4,5,8,10]
Cardiac arrest	0		21	1 [38]	0.02%; 0	(0-0.08)	6972	4 [4,5,8,10]
Respiratory arrest	0.41%; 0.42%	(0-0.82)	983	3 [38,62,63]	0.03%; 0	(0-0.10)	6972	4 [4,5,8,10]
Discontinuation due to adverse effects	9.53%; 9.52%	(1.75-20.78)	532	5 [38,47,51,54,65]	7.41%; 6.27%	(1.49-14.58)	6811	5 [3-5,10,11]
Calcium gluconate	0.70%		717	1 [63]	0.29%; 0.29%	(0.28-0.29)	5400	2 [2,5]
Any adverse effects	13.39%; 14.29%	(6.76-19.11)	826	3 [38,47,61]	56.73%; 56.97%	(24.02-88.97)	6642	4 [4,5,7,10]
Respiratory depression	1.67%; 0.72%	(0-4.76)	1363	11 [38-40,44,46,53,54,60,62-64]	2.67%; 1.02%	(0.29-10.09)	7033	5 [2-4,8,10]
Reduced/absent deep tendon reflexes	4.75%; 2.55%	(0-18.05)	1789	10 [37,40,44-46,53,61-64]	0.72%; 0.70%	(0.29-1.18)	5630	3 [2,5,8]
Hypotension	30.56%		72	1 [53]	5.59%; 1.32%	(1.05-14.39)	897	3 [3,4,8]
Flushing or warmth	52.28%; 52.28%	(4.55-100)	27	2 [42,54]	46.72%; 65.21%	(8.04-73.46)	6965	5 [4,5,8,10,11]
Nausea and/or vomiting	47.37%; 38.46%	(3.66-100)	373	3 [42,44,47]	11.84%; 9.30%	(3.15-25.61)	6898	4 [4,5,8,10]
Muscle weakness	23.08%		13	1 [44]	36.44%; 7.89%	(1.44-100)	5109	3 [1,3,5]
Drowsiness/confusion/ sleepiness/lethargy	2.58%; 2.90%	(0.28-4.55)	515	3 [47,54,64]	8.04%; 1.49%	(0.40-22.24)	5601	3 [4,5,11]
Headache	2.90%; 0.72%	(0.28-7.69)	506	3 [44,47,64]	1.06%; 1.06%	(0.72-1.40)	5285	2 [5,8]
Blurred vision	46.30%; 46.30%	(0.28-92.31)	368	2 [44,47]	7.10%		535	1 [4]
Pulmonary oedema	1.25%; 1.25%	(1.13-1.36)	649	2 [47,68]	0.57%; 0.69%	(0-0.89)	6362	4 [5,6,9,10]
Caesarean	49.68%; 56.52%	(32.53-60.00)	225	3 [37,49,62]	43.07%; 45.35%	(15.79-82.35)	7056	10 [1-6,8-11]

*Values are presented as mean and median percentage estimate from case series and from randomised trials from Comparison 1, with the range of percentages reported in the studies also presented
