# Supplementary material for: Maternal adverse effects of different antenatal magnesium sulphate regimens for improving maternal and infant outcomes: a systematic review
Source: BMC Pregnancy Childbirth. 2013 Oct 21;13:195. doi: 10.1186/1471-2393-13-195 (PMC4015216; doi:10.1186/1471-2393-13-195)
Supplement: Additional file 7 — Detail of adverse effects from case reports. [file 1471-2393-13-195-S7.pdf]

Detail of adverse effects from case reports

Adverse effect	Indication	Magnesium sulphate regimen	(RF (If APL))^	Reference
**Iatrogenic overdose (16 studies)**				
Death	Unclear<br>T<br>PE	IV ‘Overdose’<br>Free flow of IV solution<br>4 g IV bolus LD over ~ 2 mins		Anon 1990 [69]<br>Cohen 1992 [70]<br>Richards 1985 [71]
Death or persistent vegetative state	Unclear	‘Overdose’ (7 cases)		Simpson 2004 [72]
Cardiopulmonary arrest	PE<br>E<br>PE<br>PE<br>E	20 g LD<br>~ 16 g over ~ 10 mins<br>Up to 1 g/min for less than 10 mins<br>All contents of infusion bag rapidly infused<br>25 g LD		McCubbin 1981 [73]<br>McDonnell 2009 [74]<br>Morisaki 2000 [75]<br>Rabinerson 1994 [76]<br>Swartjes 1992 [77]
Cardiac arrest	T	Free flow of IV solution		Cohen 1992 [70]
Respiratory arrest	PE<br>T<br>E<br>PE	20 g over ~ 15 mins<br>~ 73 mmol in 31 mins<br>Immediate dose of 25 g<br>20 g LD		Bohman 1990 [78]<br>Cao 1999 [79]<br>McKenna 2006 [80]<br>Wax 1995 [81]
‘Life-threatening situation’	Unclear	‘Severe accidental magnesium poisoning’		Bruhwiler 1994 [82]
Coma	E	IV ‘Overdose’		Hayashi 2003 [83]
Ventilatory impairment; failure to rouse from general anaesthesia	PE	13 g over 40 mins		McDonnell 2010 [84]
Need for additional monitoring	PE<br>E	25 g over ~ 40 mins<br>~ 13 g immediate dose		Buettner 2010 [85]
Variety of adverse effects (not death/persistent vegetative state)	Unclear	‘Overdose’ (45 cases)		Simpson 2004 [72]
**Rapid administration (1 study)**				
Cardiac arrest	PE	4 g IV bolus LD over ~ 2 mins		Richards 1985 [71]
**Unintended epidural/intrathecal administration (4 studies)**				
Bilateral periumbilical pain	NA	~ 3 g ‘piggybacked’ into epidural tubing		Dror 1987 [86]
Inadequate pain relief	NA<br>NA	8.7 g over ~ 1 hour epidurally<br>9.6 g over ~ 6.5 hours epidurally		Goodman 2006 [87]
Paralysis lower extremities	NA<br>NA	2 ml 50% intrathecally<br>10 g epidurally		Lejuste 1985 [88]<br>Lewis-Younger 2004 [89]
**Increased risk of adverse effects – neuromuscular junction disorders, myopathies and neuropathologies (8 studies)**				
Weakness and/or temporary paralysis	PE<br><br>T<br>T	4 g IV LD over 20 mins; 5 g IM every 4 hours (5 doses)<br>4 g LD over ~ 30 mins<br>2 g of LD	Undiagnosed MG<br><br>Friedreich ataxia<br>Queried myotonic dystrophy	Bashuk 1990 [90]<br><br>Bruner 1990 [91]<br>Catanzarite 2008 [92]
Muscle pain; damage	T	Unclear - ceased shortly after initiation	A3243G point mutation mtDNA	Hosono 2001 [93]
Acute respiratory insufficiently	PE	10 g IM LD (5 g each buttock)	MG	Cohen 1976 [94]
Ventilatory failure	E	IV for at least 24 hours	Undiagnosed MG	Mueksch 2007 [95]
Respiratory depression	PE	5 g LD over 25 mins; 1 g/hour MD	Rare myopathy	Robins 2007 [96]
‘Magnesium toxicity’	PE	4 g IV LD; 1 g/hour MD for ~ 7 hours	A3243G point mutation mtDNA	Moriarty 2008 [97]
**Increased risk of adverse effects – renal failure (3)**				
Decreased deep tendon reflexes; prolonged QT interval	PE	4 g IV LD		Archer 2010 [98]
Muscle weakness	E	Not detailed		Chan 2008 [99]
Progressive quadriparesis	E	‘Pritchard regime’		Nethravathi 2007 [100]
**Drug interactions - agents used in general anaesthesia (10 studies)**				
Cardiac arrest	T; PE	Not detailed	Thiopental	Saitoh 1994 [101]
Respiratory arrest	PE	4 g IV ‘bolus’; 1 g/hour for 2 hours	Succinylcholine; pancuronium	Baraka 1984 [102]
Failure to achieve adequate ventilation	PE	Not detailed	Thiopentone; succinylcholine; rocuronium	Nguyen 2001 [103]
Numb; difficultly moving upper extremities	PE	Not detailed	Bupivacaine; fentanyl	Fay 1996 [104]
Prolonged neuromuscular blockade	T<br>T<br>PE	1 g/hour IV<br>Not detailed<br>4 g slow IV bolus; 1 g/hour MD for ~ 13 hours	Rocuronium<br>Vecuronium<br>Vecuronium	Funai 2010 [105]<br>Hino 1997 [106]<br>Kwan 1996 [107]
	PE	4 g IV twice; 2 g/hour MD	Vecuronium	Sinatra 1985 [108]
	PE, T	Unclear (2 cases)	Vecuronium	Yoshida 2006 [109]

	PE	2 g/hour IV	Rapacuronium	Sloan 2001 [110]
**Drug interactions - other (6)**				
Neuromuscular blockade - muscle weakness/paralysis	PE<br>T<br>PE	2 g/hour IV<br>500 mg<br>2 g IV LD; 10 g/day for ~ 3 days	Nifedipine<br>Nifedipine<br>Amlodipine	Ben-Ami 1994 [111]<br>Snyder 1989 [112]<br>Wu 2010 [113]
Severe bradycardia	PE	4 g IV LD over 30 mins (stopped at 20)	Labetalol	Pittman 2000 [114]
Severe hypotension	PE<br>PE	Unclear<br>20 g daily IV (2 cases)	Nifedipine<br>Nifedipine	Scardo 1997 [115]<br>Waisman 1998 [116]
**Unusual adverse effects (11 studies)**				
Bilateral, progressive labial swelling (need for caesarean)	T	IV for ~ 9 days		Awwad 1994 [117]
Worsened clinical picture of appendicitis and cholecystitis	T	Unclear		Basaran 2007 [118]
Impaired lactogenesis	PE	6 g IV LD over 20 mins; 2 g/hour MD until 2 days postpartum (total 102 g)		Haldeman 1993 [119]
Severe paralytic ileus	T	4 g IV LD; 1-2 g/hour MD ceased on day 3		Hill 1985 [120]
Marked osteoporotic change (hips, knees, ankles)	T	2 g IV LD over 30 mins; 1 g/hour for 101 days		Hung 2005 [121]
Breast engorgement and galactorrhea	T	4 g IV LD; 2 g/hour MD ceased on day 4		Lurie 2002 [122]
Development of central pontine myelinolysis	PE	4 g IV LD; 2 g/hour MD for 13 hours		Riggs 2000 [123]
Urinary tract stone (magnesium ammonium phosphate)	T	4 g IV LD; 1-2 g/hour for 21 days		Sameshima 1997 [124]
Hyperkalaemia and hyponatremia (hyporeninemic hypoaldosteronism)	PE<br>T	Continuous IV for ~ 6-7 days<br>IV for ~ 4 days		Spital 1991 [125]
Left retinal detachment; partial right detachment	E	2.5 g LD, 5 g over 15 mins; 2.5 g/hour MD for 48 hours		Roberts 1998 [126]
Extensive urticarial rash	T; T	4 g IV LD; 4 g IV LD, 2 g/hour MD		Thorp 1989 [127]
**Adverse effects (16 studies)**				
Severe hypotension	PE<br>PE	Unclear (serum Mg 4.5 mEq/l) (2 cases)<br>4 g IV LD; 2-3 g/hour MD for ~ 12 hours		Bourgeois 1986 [128]<br>Rodis 1987 [129]
Hypothermia	T<br>T	6 g IV LD; 2 g/hour (4 hours) 3 g/hour (4 hours) 2 g/hour (3 ½ hours)<br>4 g IV LD; 2-3 g/hour MD for ~ 12 hours		Cardosi 1998 [130]<br>Rodis 1987 [129]
Bradycardia (39-44/min)	PE	1 g/hour IV (reduced to 0.75 g/hour later)		Hennessy 1999 [131]
Atrial fibrillation (100-150/min)	PE	4 g IV LD; 2 g/hour MD ~ 2 hours		Oettinger 1993 [132]
Absent deep tendon reflexes	E	4 g IV and 10 g IM LD; additional 4 g IV		Pritchard 1979 [133]
Marked weakness; difficulty breathing	E	Unclear		Pritchard 1979 [133]
Sleepiness/fatigue; depressed/absent deep tendon reflexes	T<br>PE	4 g IV LD; 'mostly' 2.5 g/hour MD for ~ 1 day (51.4 g total)<br>5 g IV LD over 30 mins; 1.7-2 g/hour for 14 ½ hours		Herschel 2001 [134]<br>Tang 2010 [135]
Chest pain; inverted T waves (ECG) (transient subendocardial ischemia)	T	4 g IV LD; 3 g/hour MD (10 hours) reduced to 2 g/hour (total 30 hours)		Sherer 1992 [136]
Bilateral hand contractures/tetany (serum hypocalcaemia)	T<br>T	6 g IV LD; 3 g/hour MD reduced to 1.5 g/hour (132 g over 55 hours)<br>2 g IV LD; 2-3 g/hour MD (53 g over 55 hours)		Koontz 2004 [137]
Diplopia; malaise; paresthesia (serum hypocalcaemia)	T	5 g IV LD; 2 g/hour MD		Mayan 1999 [138]
Hoarseness; tetany (serum hypocalcaemia)	T	5 g IV LD; 2 g/hour MD		Mayan 1999 [138]
Hypotension; cyanosis; tetany (serum hypocalcaemia)	E	Unclear at local hospital; additional 5 g IM and 2 g IV		Monif 1972 [139]
Chest tightness and pain; prolonged QT interval (serum hypocalcaemia)	T	4 g IV LD; 3 g/hour MD ~ 20 hours, decreased 2 g/hour for ~ 1 day		Nassar 2007 [140]
Delirium with myoclonus (serum hypocalcaemia)	PE	Unclear		Ganzenvoort 2002 [141]
Pulmonary oedema	T; PE<br>T	Regimen as described by Steer and Petrie (2 cases)<br>Unclear - IV		Elliot 1979 [142]<br>Worrell 1992 [143]

^Factor that the author(s) of the original study associated with the adverse effect (i.e. general anaesthetic agent, antihypertensive agent, or neuromuscular junction disorder).

Abbreviations: APL: applicable; E: eclampsia; g: grams; IM: intramuscular; IV: intravenous; LD: loading dose; MD: maintenance dose; MG: myasthenia gravis; mtDNA: mitochondrial DNA; NA: not applicable; PE: pre-eclampsia; RF: risk factor; T: tocolysis; ~: approximately
