# Supplementary material for: Maternal adverse effects of different antenatal magnesium sulphate regimens for improving maternal and infant outcomes: a systematic review
Source: BMC Pregnancy Childbirth. 2013 Oct 21;13:195. doi: 10.1186/1471-2393-13-195 (PMC4015216; doi:10.1186/1471-2393-13-195)
Supplement: Additional file 8 — Blog and discussion forum thread sources. [file 1471-2393-13-195-S8.pdf]

Blog and discussion forum thread sources

Blog title; post title	Posted	URL
**Baby Dust Diaries;** My Big Fat Preterm Labor, Pre-eclampsia, Gestational Diabetes Pregnancy Update	Feb 2011	http://babydustdiaries.com/2011/02/13/my-big-fat-preterm-labor-pre-eclampsia-gestational-diabetes-pregnancy-update/
BIRD ON A LINE Trying, in my way, to be free; My Labor and Delivery Experience 6 Days Ago	Apr 2011	http://www.bird-on-a-line.com/2011/04/my-labor-and-delivery-experience-6-days.html
Blessings in Disguise; Finally...26 Weeks!!	Jul 2011	http://bigbrotherx3.blogspot.com/2011/07/july-3-2011-finally26-weeks.html
Butterflies and Rainbows. Remembering our butterfly baby, while hoping for our rainbow baby; Hospital admission #2	Mar 2011	http://butterflies-and-rainbows.blogspot.com/2011/03/hospital-admission-2.html
DOUBLE THE TROUBLE, DOUBLE THE FUN; The down and dirty - My Labor and Birthing Story	Mar 2011	http://ndsemanchik.blogspot.com/2011/03/down-and-dirty-my-labor-and-birthing.html
Ebonyforever.com. Random Thoughts & Ramblings; Contractions?	Feb 2010	http://www.ebonyforever.com/?p=48
Everyday Childhood with...Kelly Loubet; Pregnancy Hospitalizations: A Walk Down Memory Lane	May 2011	http://everydaychildhood.com/2011/05/pregnancy-hospitalizations-a-walk-down-memory-lane/
From the Heart; Part II: My C-Section Woes	Jul 2011	http://eafromtheheart.blogspot.com/2011/07/part-ii-my-c-section-woes.html
Learning a new life; Contractions, Contractions, go away!	May 2011	http://mamaoflydia.blogspot.com/2011/05/contractions-contractions-go-away.html
My Birth Class; Pregnancy# 1 Giana's Birth	Jun 2011	http://mybirthclass.blogspot.com/2011/06/pregnancy-1-gianas-birth.html
Our Er-Lee Arrival (Preemie Mom); Magnesium Sulfate - A miracle drug, or perfectly legal torture? :-)	Jun 2011	http://ourerleearrivals.wordpress.com/2011/06/13/magnesium-sulfate-a-miracle-drug-or-perfectly-legal-torture/
OUR OWN KIND OF PERFECT; 154/106 And it went downhill from there...	Feb 2010	http://homykhappenings.blogspot.com/2010/04/154106.html
Some Youngs; 5 Years	Apr 2011	http://someyoungs.blogspot.com/2011/04/5-years.html
Steece's Pieces. Prayed for one, blessed with four!; Baby Week on DHC! The Birth Story of the Steece Quadruplets	Jun 2009	http://thelifeofsuz.blogspot.com/2009/06/baby-week-on-dhc-birth-story-of-steece.html
The Birth Teacher. Informed choices. Positive births.; Preterm labor	Mar 2009	http://thebirthteacher.blogspot.com/2009/03/preterm-labor.html
The Morris Family. Life is Beautiful; Magnesium Sulfate	Apr 2008	http://themorrisfamily-blog.blogspot.com/2008/04/magnesium-sulfate.html
The Pajama Mama; Birth Story Aftermath: Re-Hospitalization	Mar 2011	http://thepajamamama.wordpress.com/2011/03/16/birth-story-aftermath-re-hospitalization/
This Crazy Life; Pre-eclampsia. May is Preeclampsia Month	May 2011	http://thiscrazylife-amy.blogspot.com/2011/05/pre-eclampsia.html
Twin Talk; Preterm labor. Preeclampsia?	Feb 2010	http://www.twin-talk.com/2010/02/preeclampsia.html
Writing Roads. Write where you want to go; Tomato soup of the leg	Sept 2010	http://writingroads.com/blog/2010/09/tomato-soup-of-the-leg/

Discussion forum or group; thread	Dated	URL
pPROM Support Group; Magnesium Sulfate	Jul 2010	http://community.babycenter.com/
April 2010 Birth Club; Magnesium Sulfate	Mar 2010	
Bedrest Club; Magnesium sulfate long term	Nov - Dec 2010	
Large Families; Magnesium Sulfate - any experiences?	Feb 2011	
November 2009 Turkey Babies EDD 11/21-11/30; Magnesium Sulfate during labor for pre-eclampsia?	Oct 2009	
Pregnancy Issues; Magnesium sulfate treatment and pre-term labour???	Apr – year unknown	http://forum.baby-gaga.com/
Pregnancy Issues; magnesium sulfate??	Mar - Apr 2009	
Pregnancy Issues; Moms who have been on Magnesium Sulfate?	Aug 2009	
Pregnancy Issues; Magnesium Sulfate Question.	Sept 2010	
Labor and Birth; Magnesium for stopping labor	Jan 2010	
Fit Moms Pregnancy and Postpartum; Breast milk and magnesium sulfate?	Jun 2008	http://cathe.com/forum/
Pregnancy Support Group; Should I refuse magnesium sulfate?	Nov 2007	http://www.dailystrength.org/c/Pregnancy/forum
Pregnancy Support Group; Magnesium	Nov 2008	

Expectant Moms; Experience w/ magnesium sulfate?	Mar 2010	http://www.dcurbanmom.com/
Expectant Moms; blood pressure inconsistency at Doctor's office - no other pre-e symptoms	Jul 2009	
Expectant Moms; Preeclampsia	Sept 2010	
Expectant Moms; Induction for signs of preeclampsia at 37.5 weeks?	Mar 2010	
Expectant Moms; high blood pressure - how bad is this news?	Aug 2011	
Babies and Kids With Disabilities & Special Needs; Preeclampsia Second Pregnancy	Sept 2010	http://www.essentialbaby.com.au/forums/
Babies Born Early (and beyond); Has anyone been given magnesium Sulfate therapy?	Jan 2008	
Pregnancy: Tips, Questions and Information; Eclampsia - study has found...Epsom salts could halve the risk of developing it!	Aug 2010	
Specialised Pregnancy Support Groups; Pre-Eclampsia Support BG - #11	Jul 2008	
Babies Born Early (and beyond); Mag Sulfate???	Mar 2005	
Breastfeeding Support: Ask the Lactation Consultant; Magnesium sulfate...anyone BTDT? (long)	Aug 2005	http://forums.ivillage.com/
High Risk & Pregnancy Complications; magnesium sulfate	Apr 2003	
April 2007 Expecting Club; Magnesium sulfate - preterm labor - ??	Jan 2007	
May 2008 Expecting Club; Anyone have magnesium sulfate?	May 2008	
January 2006 Expecting Club; Premie BTDT? Magnesium? very scared!	Mar 2005	
Pregnancy; pre eclampsia with 1st baby	Jan 2006	http://www.mumsnet.com/
Labor; Please pray for us *update*	Jul - year unknown	http://www.pregnancythisweek.com/
Pregnant; Preterm labor	Jul - year unknown	
High risk pregnancy; Im in labor at 27 weeks :'(	Jul - year unknown	
High risk pregnancy; So scared...21 weeks in l&d update #2 update #3	Aug - year unknown	
May 2011 Babies; Magnesium Sulfate	Apr 2011	http://www.whattoexpect.com/
July 2011 Babies; magnesium sulfate sucks!	May 2011	
Multiples and Twins; magnesium sulfate	Dec - year unknown	
April 2011 Babies; Joined the magnesium sulfate club...	Feb 2011	
August 2011 Babies; went to the doctor and the doctor said	Feb 2011	
